# Transient Receptor Potential V Channels Are Essential for Glucose Sensing by Aldolase and AMPK

**DOI:** 10.1016/j.cmet.2019.05.018

**Published:** 2019-09-03

**Authors:** Mengqi Li, Chen-Song Zhang, Yue Zong, Jin-Wei Feng, Teng Ma, Meiqin Hu, Zhizhong Lin, Xiaotong Li, Changchuan Xie, Yaying Wu, Dong Jiang, Ying Li, Cixiong Zhang, Xiao Tian, Wen Wang, Yanyan Yang, Jie Chen, Jiwen Cui, Yu-Qing Wu, Xin Chen, Qing-Feng Liu, Jianfeng Wu, Shu-Yong Lin, Zhiyun Ye, Ying Liu, Hai-Long Piao, Li Yu, Zhuan Zhou, Xiao-Song Xie, D. Grahame Hardie, Sheng-Cai Lin

**Affiliations:** 1 State Key Laboratory for Cellular Stress Biology, Innovation Center for Cell Signaling Network, School of Life Sciences, Xiamen University, 361102 Fujian, China; 2 State Key Laboratory of Membrane Biology, Institute of Molecular Medicine, PKU-IDG/McGovern Institute for Brain Research, Peking University, 100871 Beijing, China; 3 State Key Laboratory of Membrane Biology, School of Life Sciences, Tsinghua University, 100084 Beijing, China; 4 CAS Key Laboratory of Separation Science for Analytical Chemistry, Scientific Research Center for Translational Medicine, Dalian Institute of Chemical Physics, Chinese Academy of Sciences, 116023 Dalian, China; 5 State Key Laboratory of Membrane Biology, Institute of Molecular Medicine, Peking-Tsinghua Center for Life Sciences, Peking University, 100871 Beijing, China; 6 McDermott Center of Human Growth and Development MC8591, University of Texas Southwestern Medical Center, Dallas, TX 75390, USA; 7 Division of Cell Signalling and Immunology, College of Life Sciences, University of Dundee, Dundee DD1 5EH, Scotland

**Keywords:** glucose sensing, AMP-activated protein kinase, AMPK, transient receptor potential channels, TRPV, aldolase, v-ATPase

## Abstract

Fructose-1,6-bisphosphate (FBP) aldolase links sensing of declining glucose availability to AMPK activation via the lysosomal pathway. However, how aldolase transmits lack of occupancy by FBP to AMPK activation remains unclear. Here, we show that FBP-unoccupied aldolase interacts with and inhibits endoplasmic reticulum (ER)-localized transient receptor potential channel subfamily V, inhibiting calcium release in low glucose. The decrease of calcium at contact sites between ER and lysosome renders the inhibited TRPV accessible to bind the lysosomal v-ATPase that then recruits AXIN:LKB1 to activate AMPK independently of AMP. Genetic depletion of TRPVs blocks glucose starvation-induced AMPK activation in cells and liver of mice, and in nematodes, indicative of physical requirement of TRPVs. Pharmacological inhibition of TRPVs activates AMPK and elevates NAD^+^ levels in aged muscles, rejuvenating the animals’ running capacity. Our study elucidates that TRPVs relay the FBP-free status of aldolase to the reconfiguration of v-ATPase, leading to AMPK activation in low glucose.

## Highlights

• TRPV cation channels are required for glucose starvation-induced AMPK activation
• FBP-unoccupied aldolase inhibits Ca^2+^ release of TRPVs at the ER-lysosome contact
• The decrease of the local Ca^2+^ allow TRPV proteins to interact with v-ATPase
• TRPVs interact with and alter v-ATPase to initiate activating complex for AMPK

## Graphical Abstract

**Figure:**
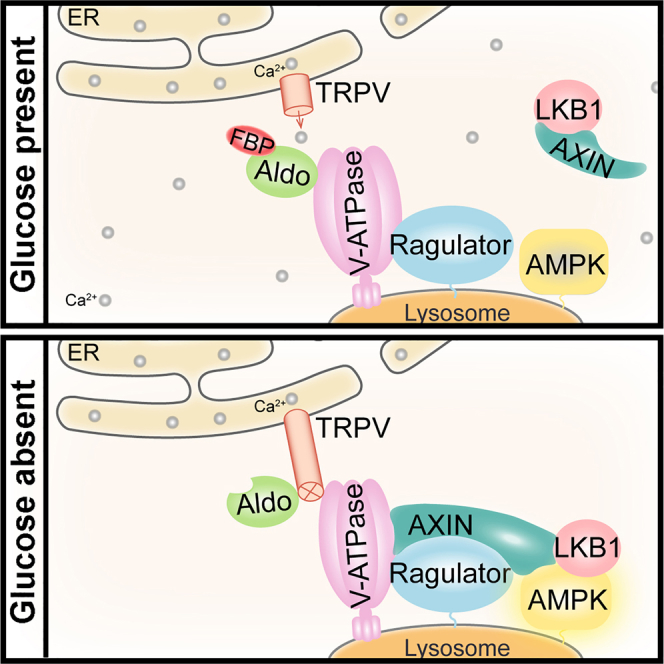

## Teaser

Falling levels of glucose, and consequentially fructose-1,6-bisphosphate (FBP), are sensed by glycolytic enzyme aldolase. Li et al. demonstrate that FBP-unoccupied aldolase binds to and inhibits ER-localized TRPV channels, with the decreased calcium at the ER-lysosome contact enabling the channel proteins to interact with lysosomal v-ATPase to allow for AMPK activation.

## Context and Significance

**Cells contain an internal energy sensor called AMP-activated protein kinase (AMPK), which responds to decreased nutrient and energy levels by activating cellular pathways to replenish energy levels. Researchers at Xiamen University in China dissect the molecular pathways underlying AMPK activation in response to glucose deprivation and link calcium signaling, another important cellular pathway, through transient receptor potential channels (TRPV) to AMPK responses. This pathway is evolutionarily conserved, and TRPV seems to act as an electric capacitor to provide graded energy responses. Given that aging and decreased muscular fitness is associated with decreased AMPK, the investigators were able to pharmacologically increase AMPK levels in aged muscles, something that has previously proved quite challenging, and show that the treated aged mice doubled their running capacity.**

## Introduction

AMP-activated protein kinase (AMPK) is a pivotal sensor for monitoring cellular energy state and nutrient supply (Carling et al., 2012, Herzig and Shaw, 2018, Lin and Hardie, 2018, Steinberg and Kemp, 2009). It occurs universally as heterotrimeric complexes containing catalytic α subunits and regulatory β, and γ subunits, with the γ subunit providing the binding sites for the regulatory adenine nucleotides, AMP, ADP, and ATP. When cells encounter metabolic stress, an increase of AMP:ATP and ADP:ATP ratios occurs. Binding of AMP to AMPK causes allosteric activation. Moreover, binding of AMP or ADP, also enhances phosphorylation of Thr172 on the α-subunit by the upstream kinase liver kinase B1 (LKB1), and inhibits Thr172 dephosphorylation by protein phosphatases; all three effects are opposed by binding of ATP (Ross et al., 2016). Thr172 can also be phosphorylated by the alternative upstream kinase Ca^2+^/calmodulin-dependent protein kinase kinase-2 (CaMKK2/CaMKKβ) in response to increases in cytosolic Ca^2+^ concentration (Hawley et al., 2005, Hurley et al., 2005, Woods et al., 2005). Once activated, AMPK phosphorylates a wide range of downstream targets to maintain energy homeostasis, switching on catabolic pathways that generate ATP while switching off ATP-consuming processes (Herzig and Shaw, 2018). We have recently shown that glucose deprivation activates AMPK in an AMP/ADP-independent manner through a mechanism involving the scaffold protein axis inhibitor protein (AXIN) (Zhang et al., 2017). AXIN binds LKB1 constitutively, but glucose deprivation promotes formation of a ternary complex that includes the downstream kinase AMPK (Zhang et al., 2013). This occurs on the surface of the lysosome and requires both the vacuolar H^+^-ATPase (v-ATPase, which acidifies the lumen of lysosomes) and the pentameric Ragulator complex containing LAMTOR1-LAMTOR5 (Zhang et al., 2014). The v-ATPase-Ragulator complex is also required in a reciprocal manner for activation of mTORC1 when nutrient levels are high, thus exerting a reversible switch between catabolism and anabolism (Zhang et al., 2014). Upon glucose deprivation, a proportion of aldolase becomes unoccupied by fructose-1,6-bisphosphate (FBP) and acts as a sensor of glucose availability to promote docking of AXIN:LKB1 to the v-ATPase:Ragulator complex, which in turn leads to the formation of a “super-complex” comprised of the v-ATPase, Ragulator, AXIN, LKB1, and AMPK on the lysosomal surface, referred to below as the AXIN-based complex, and thereby allows LKB1 to phosphorylate and activate AMPK (Zhang et al., 2017, Zhang et al., 2014, Zhang et al., 2013). Importantly, inhibition of v-ATPase upon glucose starvation is a prerequisite step in triggering the lysosomal pathway because concanamycin A (ConA), a v-ATPase inhibitor (Drose and Altendorf, 1997), is able to directly promote the formation of the AXIN-based complex even under normal glucose conditions (Zhang et al., 2014). However, it remained unclear how the FBP-unoccupied aldolase leads to inhibition of v-ATPase that allows for the formation of the AXIN-based complex.

## Results

### TRPV1–4 Interact with FBP-Unoccupied Aldolase and Are Required for Lysosomal AMPK Activation in Low Glucose

**Figure 1:**
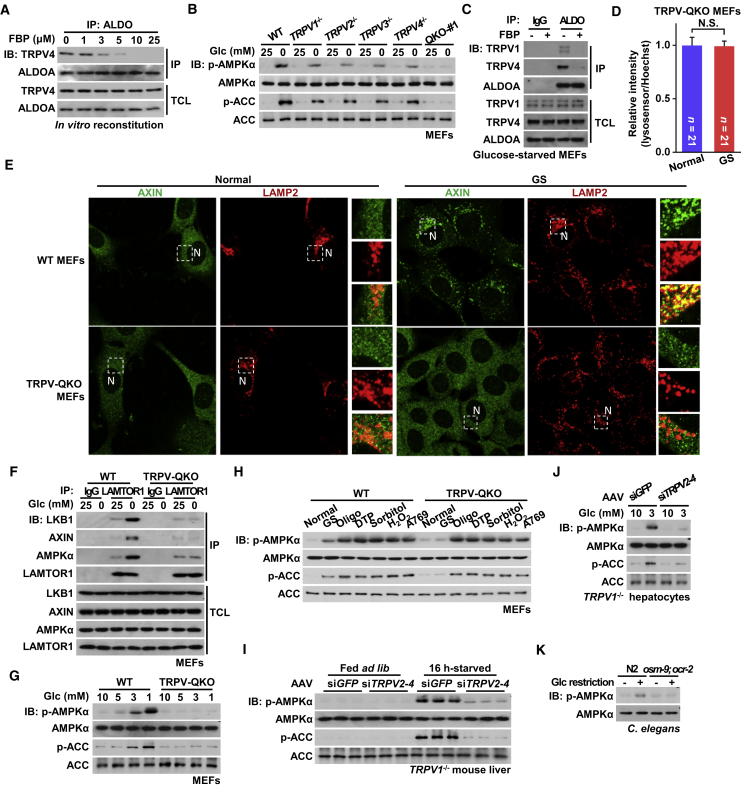
TRPV1–4 Interact with FBP-Unoccupied Aldolase and Are Physically Required for Lysosomal AMPK Activation in Low Glucose (A) FBP at physiologically relevant concentrations abolishes the interaction between endogenous TRPV4 and aldolase. Light organelles were prepared from 2-h glucose-starved MEFs and aliquoted, followed by addition of FBP at concentrations indicated. Aldolases (represented by ALDOA) were immunoprecipitated, followed by immunoblotting using the indicated antibodies. Note that the polyclonal antibody raised in rabbit by His-tagged ALDOA was able to react with all three isozymes of aldolase, as validated in Figure S1G. The antibody against endogenous TRPV4 was validated in Figure S1B. (B) TRPV channels are functionally redundant in mediating AMPK activation under glucose starvation. TRPV1^−⁄−^, TRPV2^−⁄−^, TRPV3^−⁄−^, TRPV4^−⁄−^, TRPV-QKO MEFs, and wild-type (WT) MEFs as control, were incubated in DMEM with or without 25-mM glucose for 2 h, followed by analysis of p-AMPKα and p-ACC. AMPK activation was virtually abolished in TRPV-QKO MEFs in which no TRPV was detectable by the antibodies. (C) FBP dampens the association between endogenous TRPVs and aldolase. Lysates prepared from 2-h glucose-starved MEFs were mixed with 10 μM FBP. Aldolase was immunoprecipitated, followed by immunoblotting using the indicated antibodies. The antibody against endogenous TRPV1 was validated in Figure S2B (left panel). (D) TRPVs are required for inactivation of lysosomal v-ATPase in response to glucose starvation. TRPV-QKO MEFs were pre-loaded with LysoSensor Green DND-189 and Hoechst, and were then cultured in DMEM with 25-mM glucose (normal) or DMEM without glucose (GS) for 2 h. The relative fluorescent intensities of LysoSensor (normalized to the intensity of Hoechst) were then analyzed. Results are mean ± SEM; n value of each group is directly labeled on the bar, and the same below. p value was determined by Student’s t test. Representative images of this experiment are shown in Figure S2D. (E) TRPVs are required for the lysosomal translocation of AXIN. MEFs were glucose starved and the localization of AXIN was determined by immunofluorescent staining. Endogenous AXIN (green, validated previously, (Zhang et al., 2014)) and LAMP2 (red) were stained with goat anti-AXIN antibody and rat anti-LAMP2 antibody, respectively, and were imaged by confocal microscopy. “N” indicates nucleus. (F) TRPV proteins are required for the formation of Ragulator:AXIN:LKB1:AMPK complex. Endogenous LAMTOR1 in regularly cultured (in DMEM containing 25-mM glucose) or glucose-starved TRPV-QKO MEFs was immunoprecipitated, followed by immunoblotting. (G) TRPVs are required for the activation of AMPK in physiologically low glucose. TRPV-QKO MEFs were incubated in DMEM containing 1, 3, 5, or 10 mM glucose for 2 h, followed by analysis of p-AMPKα and p-ACC. (H) TRPV channels are specifically involved in the AMPK activation induced by glucose starvation. TRPV-QKO MEFs, along with its wildtype control, were starved for glucose (GS) or treated with 1 μM oligomycin, 50-μM dinitrophenol (DTP), 500 μM sorbitol, 1 mM H_2_O_2_ or 200 μM A769662, for 2 h, followed by analysis of p-AMPKα and p-ACC. (I and J) TRPVs are required for starvation-induced AMPK activation in mouse liver and primary hepatocytes. TRPV1^−⁄−^ mice were injected with a combination of AAV-carried siRNAs against *TRPV2*, *TRPV3,* and *TRPV4*. After 4 weeks of injection, p-AMPKα and p-ACC levels in livers from 16-h starved mice (I) and in hepatocytes incubated in medium containing 3-mM glucose (resembling the concentration of plasma glucose detected in 16-h starved mice, as shown in Figure S2J) for 12 h (J) were analyzed. (K) Requirement of TRPVs for glucose restriction-induced AMPK activation is conserved in nematodes. Worms (3 days old) of the strain lacking TRPVs (*osm*-*9*; *ocr*-*2*), along with its wild-type control (N2), were cultured on normal NGM agar or NGM agar containing 5 mM 2-DG for 20 h (fed with live OP50 bacteria). The worms were then lyzed, and the levels of p-AMPKα and p-ACC were determined by immunoblotting. Experiments in this figure were performed at three times, except for (I), (J), and (K) twice. See also Figures S1 and S2.

To search for factors that might transmit sensing of the absence of FBP by aldolase to the conformational changes of v-ATPase, we performed mass spectrometry of protein complexes co-immunoprecipitated with ALDOA from light organelle fractions containing lysosomes. Some 114 proteins with cutoff scores >3.69 were obtained (Table S1), which could be divided into four major categories, as summarized in Figure S1A. Among them, there were multiple known interactors of aldolase, including the v-ATPase subunits V1A, V1B2, and V1E1 (Bond and Forgac, 2008, Lu et al., 2007, Lu et al., 2001, Lu et al., 2004), the glycolytic enzymes GAPDH and PFK1 (Ouporov et al., 2001, Ovádi and Srere, 2000), and components of actin and tubulin filaments (Carr and Knull, 1993, Wang et al., 1996). We were intrigued by one candidate, TRPV4, which belongs to the V subfamily of transient receptor potential (TRP) channels, which play a variety of roles in different tissues in response to chemical and physical stimuli (Clapham, 2003; Nilius and Flockerzi, 2014). As with other members of this family, TRPV4 has been shown to be localized both to the plasma membrane and endomembrane systems, including the endoplasmic reticulum (ER) (Wen et al., 2017). We also confirmed that a portion of TRPV4 is localized on ER by performing subcellular fractionation (Figures S1B and S1C). We next carried out co-immunoprecipitation assays to validate the interaction between aldolase and TRPV4 in the presence or absence of FBP. Co-immunoprecipitation was detected between TRPV4 and all the three isozymes of aldolase (Figure S1D). Importantly, these interactions were virtually abolished in the presence of 10 μM (and above) FBP (Figure 1A), which is within the range of FBP concentrations in cells cultured with normal glucose (Zhang et al., 2017). These results demonstrated that aldolase interacts with TRPV4 only in the absence of FBP.

We next tested whether TRPV4 is involved in regulating the lysosomal AMPK activation pathway. However, unlike LAMTOR1 deficiency, which leads to a defect of AXIN/LKB1 docking onto the lysosome for AMPK activation (Zhang et al., 2014), knockout of *TRPV4* in MEFs only partially blocked the activation of AMPK in low glucose, as determined by the phosphorylation of Thr172 on AMPKα and of the primary site on the AMPK downstream target, ACC (Figure 1B), a key marker for determining AMPK activity in intact cells (Gowans et al., 2013). The mammalian TRPV subfamily contains five other structurally conserved members (Nilius and Flockerzi, 2014), suggesting that other family members might be involved in AMPK activation. MEFs expressed mRNAs encoding *TRPV1–4*, with that for *TRPV4* being the most abundant, while *TRPV5* and *TRPV6* mRNAs were not detected (Figure S1E); however, only TRPV1 and TRPV4, and not TRPV2 or TRPV3, were detectable by western blotting. Validation of the TRPV1 and TRPV4 antibodies, using lysates from TRPV1^−⁄−^ or TRPV4^−⁄−^ MEFs, are shown in Figures S1B and S2B. We next tested interactions between TRPV1 or TRPV4 and aldolase, using either ectopically expressed or endogenous proteins. TRPV1–3, like TRPV4, all showed interactions with aldolase in the absence of FBP (Figures 1C, S1F, and S1H–S1J; antibody against endogenous aldolase was validated in Figure S1G), and similar to that of TRPV4, single knockout of *TRPV1*, *TRPV2,* or *TRPV3* only mildly impaired the activation of AMPK upon glucose starvation (Figure 1B). We therefore carried out a quadruple knockout of TRPV1–4 in MEFs (TRPV-QKO, Figures S2A and S2B), and found that this markedly impaired the effects of glucose starvation on the inhibition of v-ATPase, lysosomal translocation of AXIN, formation of the AXIN-based complex, and AMPK activation (Figures 1B, 1D, 1F, S2C, and S2D). Importantly, the requirement of TRPVs covered the entire physiological range of glucose (5 mM and below) for AMPK activation *in vivo* (Figure 1G) (Zhang et al., 2017). Re-introduction of a single TRPV channel by stable expression in TRPV-QKO MEFs restored AMPK activation upon glucose starvation (Figure S2E, and mRNA levels of four re-introduced TRPVs were validated in Figure S2F). These results confirmed that TRPV1–4 are functionally redundant in glucose sensing in MEFs. They appear to be specifically involved in AMPK activation induced by glucose starvation, because treating TRPV-QKO MEFs with oligomycin or dinitrophenol (inhibiting oxidative metabolism), sorbitol (generating osmotic stress), hydrogen peroxide (generating oxidative stress), or A769662 (direct binding to AMPK) (Langendorf and Kemp, 2015, Xiao et al., 2013), all still led to robust AMPK activation (Figure 1H). The importance of TRPV1–4 in AMPK activation was further supported by genetic evidence that starvation-induced AMPK activation was severely impaired in mouse liver or primary hepatocytes from TRPV1^−⁄−^ mice with knockdown of *TRPV2–4* (Figures 1I, 1J, S2H, and S2I, note that in mouse liver, TRPV5 and TRPV6 were not expressed, as validated in Figure S2G) with the plasma glucose levels unchanged compared with wild-type littermates (Figure S2J). Importantly, in *Caenorhabditis elegans* mutants lacking both *osm-9* and *ocr-2*, the mammalian TRPV orthologs (Colbert et al., 1997, Tobin et al., 2002, Zhang et al., 2004), glucose restriction failed to activate AMPK (Figures 1K). Thus, TRPV members represent newly identified factors that play essential roles in glucose-sensing and AMPK activation via the lysosomal pathway in an evolutionarily conserved manner.

### Glucose Starvation Blocks the Ca^2+^ Channel Activity of TRPVs

**Figure 2:**
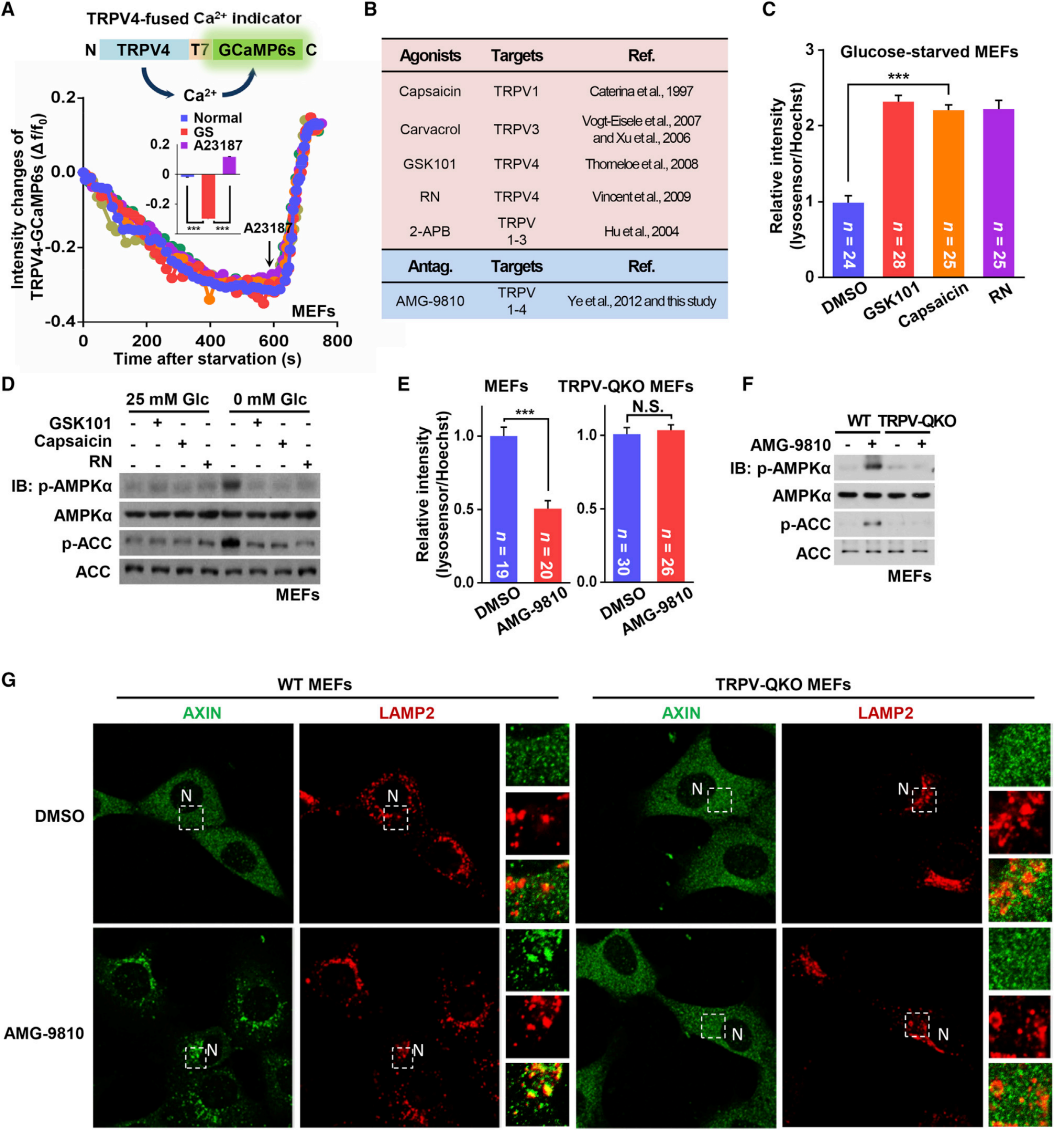
Glucose Starvation Blocks Ca^2+^ Channel Activity of TRPVs (A) Glucose starvation inhibits the activity of TRPV4 channel. A schematic diagram of the Ca^2+^ sensor TRPV4-GCaMP6s is shown on the top, and validation of the sensor is shown in Figures S3A and S3B. MEFs stably expressing TRPV4-GCaMP6s were treated with glucose-free DMEM, then 5 μM A23187 (at 600 s). The fluorescent images of TRPV4-GCaMP6s were taken at a regular interval in MEFs after 2-min incubation with the fresh medium at 37°C, and the activities of TRPV4, expressed as the change of fluorescence intensity of GCaMP6s relative to the resting fluorescence intensity (Δf/f_0_) were plotted. The changes in fluorescence intensities of the indicator (Δf) were calculated by subtracting the resting fluorescence intensity of the indicator (f_0_) from each value of absolute fluorescent intensity of the indicator (f). Data shown are selected traces from the 6 cells from 5 dishes/experiments (lower panel). Statistical results were shown as mean ± SEM; p value by ANOVA. (B) Summary of the features of TRPV agonists and antagonists. Individual agonists and antagonists (antag.) along with their cognate TRPV isoforms are tabulated. (C) TRPV agonists restore the acidification of lysosomes under glucose starvation. MEFs preloaded with LysoSensor Green DND-189 and Hoechst were glucose starved for 2 h, followed by addition of 50 nM GSK101, 100 nM capsaicin, or 0.7 μM RN for another 15 min. The relative fluorescent intensities of LysoSensor (normalized to the intensity of Hoechst) were then analyzed. Results are mean ± SEM; p value by ANOVA. The representative images of this experiment are shown in Figure S3F. (D) TRPV agonists block glucose starvation-induced AMPK activation. MEFs were incubated in DMEM with or without 25-mM glucose for 2 h, then treated with TRPV agonists as in (C), and analyzed by immunoblotting using the indicated antibodies. (E) AMG-9810, an antagonist of TRPV1–4, inhibits acidification of lysosomes in normal glucose. Regularly cultured WT and TRPV-QKO MEFs were treated with 5 μM AMG-9810 for 30 min, and the pH of lysosome was analyzed as in (C). The representative images were shown in Figure S4C. (F) AMG-9810 activates AMPK in a TRPV-dependent manner. Regularly cultured WT and TRPV-QKO MEFs were treated as in (E), followed by analysis of p-AMPKα and p-ACC. (G) AMG-9810 triggers lysosomal translocation of AXIN in normal glucose. Regularly cultured WT and TRPV-QKO MEFs were treated as in (E), and the lysosomal translocation of AXIN was determined by immunofluorescent staining as described in Figure 1E. Experiments in this figure were performed three times. See also Figures S3 and S4.

We next tested whether the channel activity of TRPVs is affected by glucose starvation. We engineered a Ca^2+^ indicator by fusing GCaMP6s (Chen et al., 2013) to the cytoplasmic tail of TRPV4. The channel activity was directly monitored by the green fluorescence elicited by the indicator upon binding Ca^2+^ (the proper subcellular localization of the indicator was validated in Figure S3A; and the intact function of the fused indicator in sensing calcium was validated in Figures S3B and S3C). Indeed, the TRPV4-GCaMP6s fluorescent signal was strongly inhibited within 10 min of glucose starvation (Figure 2A) without any alteration of the cytosolic pH (Figure S3D), by which time AMPK is fully activated (Zhang et al., 2017).

Many agonists for TRPV channels have been identified, including capsaicin (Caterina et al., 1997) for TRPV1, carvacrol (Xu et al., 2006) for TRPV3, and GSK101 (Thorneloe et al., 2008) and RN1747 (Vincent et al., 2009) for TRPV4 (Figure 2B). Consistently, capsaicin, carvacrol, RN1747, or GSK101 led to robust increases in fluorescence of the Ca^2+^-sensitive dye, Fura-2, in glucose-starved MEFs (Figure S3E). The three agonists also increased the acidity in lysosomes in glucose-starved MEFs, as evidenced by the increased signal of the LysoSensor Green DND-189 dye (Figures 2C and S3F) whose signal increases when lysosomal pH is lowered (Cousin and Nicholls, 1997), indicating that they restored the activity of the v-ATPase in glucose-starved cells. Consistently, these agonists blocked the lysosomal translocation of AXIN and AMPK activation upon glucose starvation (Figures 2D, S3G, and S3H). Similar effects were observed using 2-APB (TRPV1–3 agonist [Hu et al., 2004]) in *TRPV1*/3/4 triple KO (TKO) MEFs in which only TRPV2 was expressed (Figures S3I and S3J). No change in AMP:ATP and ADP:ATP ratios was observed in glucose-starved MEFs following treatment with these agonists, unlike phenformin (Figure S3K). These results further confirmed that TRPV1–4 although expressed at different levels, can function redundantly upstream of the v-ATPase in MEFs (Figures 1B, S1E, and S2E). These results demonstrate that it is the v-ATPase activity that is regulated by TRPVs, and which in turn regulates AMPK activation.

We also used TRPV antagonists to test whether blocking the channels would mimic glucose starvation. AMG-9810 is an antagonist originally designed to inhibit TRPV1 (Gavva et al., 2005), but several studies have found that it also inhibits other TRPV family members such as TRPV4 (Ye et al., 2012). Indeed, AMG-9810 dampened the Fura-2 signal in *TRPV1*^*−⁄−*^ MEFs (Figure S4A). AMG-9810 also dampened the Fura-2 signal in TRPV-QKO MEFs rescued by expression of TRPV2, TRPV3, or TRPV4 (Figure S4B). We found that AMG-9810 directly triggered lysosomal complex formation and AMPK activation in MEFs cultured in normal glucose, but not in TRPV-QKO MEFs (Figures 2E, 2F, 2G, S4C, and S4D). As an additional control, no further decrease in TRPV4-GCaMP6s signal was detected in MEFs pre-treated with AMG-9810 (Figure S4E). Importantly, knockout of *LKB1*, but not *CaMKK2*, abolished AMPK activation by AMG-9810 (Figures S4F and S4G). We also confirmed that there is no change in AMP:ATP and ADP:ATP ratios in MEFs treated with AMG-9810 (Figure S4H). Thus, TRPVs, at least 1–4, in their closed state triggers the lysosomal AMPK activation pathway upstream of the v-ATPase:Ragulator complex.

### TRPVs Maintain a Compartmentalized Ca^2+^ Concentration at ER-Lysosome Contact Sites in Normal Glucose

**Figure 3:**
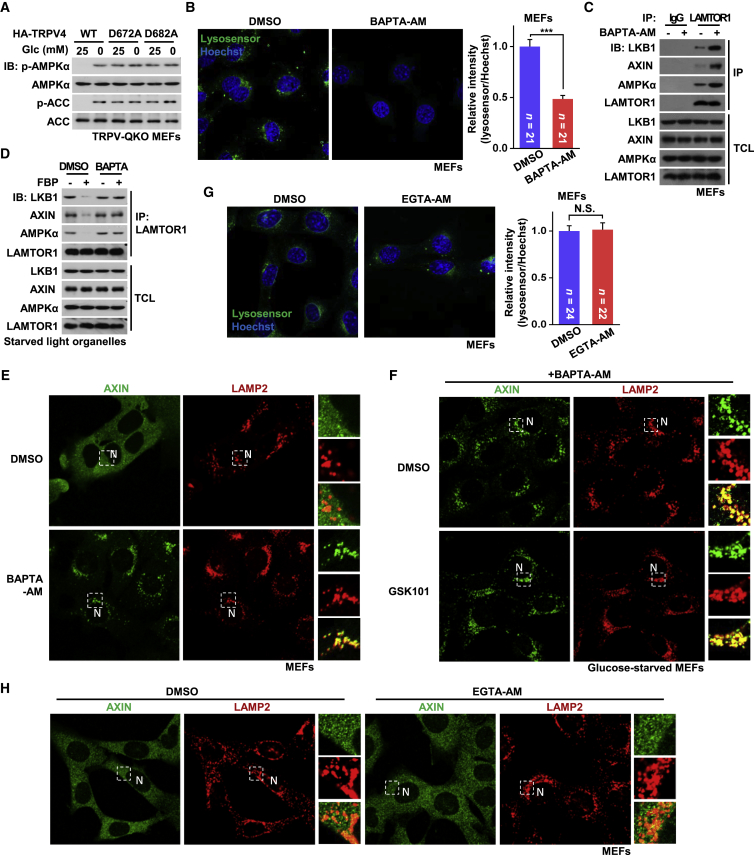
TRPVs Maintain a Local Ca^2+^ in Normal Glucose (A) Ca^2+^ releasing-defective TRPV4 mimics glucose starvation causing constitutive AMPK activation. TRPV-QKO MEFs expressing channel activity-defective HA-tagged TRPV4-D672A, TRPV4-D682A, or WT TRPV4 as a control, were incubated in DMEM with or without glucose for 2 h, followed by analysis of p-AMPKα and p-ACC. (B, C, and E) Depletion of Ca^2+^ by BAPTA-AM, cell-permeable form of the local Ca^2+^ chelator BAPTA, triggers the lysosomal AMPK pathway. Regularly cultured MEFs were treated with 100 μM BAPTA-AM for 30 min, followed by analysis of lysosomal pH (B; results are shown as mean ± SEM; p value by Student’s t test), the complex formation of Ragulator:AXIN:LKB1:AMPK (C), and the lysosomal translocation of AXIN (E). (D) BAPTA blocks FBP from dissociating AXIN-LKB1 from LAMTOR1 on light organelles. Light organelles purified from 2-h-glucose-starved MEFs were incubated with 10 μM FBP in the presence or absence of 500 μM BAPTA. Endogenous LAMTOR1 was then immunoprecipitated, followed by immunoblotting. (F) BAPTA-AM blocks TRPV agonists-prevented lysosomal dissociation of AXIN. MEFs were treated as in Figure 2C, except that 100 μM BAPTA-AM was added together with TRPV agonists. (G) EGTA-AM (cell-permeable EGTA) that is unable to chelate local Ca^2+^, cannot affect lysosomal pH. Regularly cultured MEFs were treated with 100 μM EGTA-AM for 30 min, and the pH of lysosome was analyzed. Results are mean ± SEM; p value by Student’s t test. (H) EGTA-AM fails to trigger the lysosomal translocation of AXIN. Regularly cultured MEFs were treated with 100 μM EGTA-AM for 30 min, and the localization of AXIN was determined by immunofluorescent staining. Experiments were performed three times, except (A) twice. See also Figure S5.

**Figure 4:**
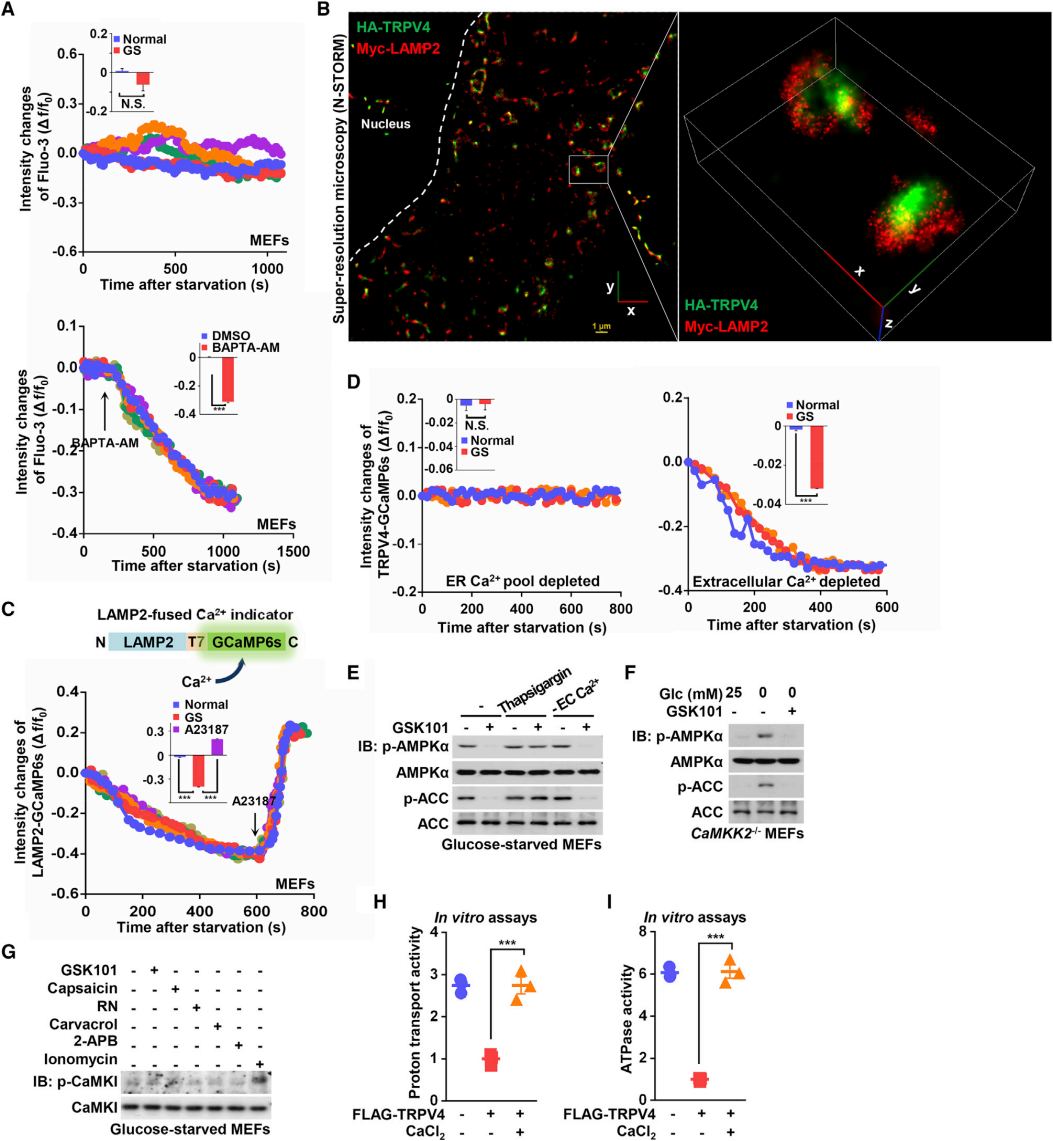
TRPV Releases Ca^2+^ Locally from ER to the Space Formed by the Contact between ER and Lysosome (A) Glucose starvation does not lead to significant change of global Ca^2+^. MEFs were preloaded with Fluo-3-AM and starved for glucose (upper graph, 5 cells), with 100 μM BAPTA-AM added as a control (lower graph, 6 cells). Statistical results were graphed as mean ± SEM; p value by Student’s t test. (B) STORM images of TRPV4 and LAMP2 in MEFs showing that the two markers are apposed. HA-TRPV4 (green) and Myc-LAMP2 (red) expressed in TRPV4^−⁄−^ MEFs (both expressed at close-to-endogenous levels driven by pBOBI vector) were stained with rabbit anti-HA antibody and mouse anti-Myc antibody, respectively. The boxed areas are enlarged on the right side. The proper localization of HA-tagged TRPV4 was validated in Figure S5E. (C) Glucose starvation leads to a decrease of Ca^2+^ concentration in the vicinity of lysosomes. MEFs were starved for glucose, and then treated with 5 μM A23187 (at 600 s). Data shown are selected traces from the 5 cells from 2 dishes/experiments. Statistical results were graphed as mean ± SEM; p value by ANOVA. (D) TRPV4-released Ca^2+^ originates from the ER pool. MEFs were treated with 4 μM thapsigargin for 15 min to deplete the ER Ca^2+^ pool, or incubated in Ca^2+^-free DMEM containing 5 mM EGTA to remove extracellular Ca^2+^ for 30 min, followed by determination of the fluorescent signal of the indicator TRPV4-GCaMP6s. Data shown are selected traces from the 3 cells from 2 dishes/experiments, and were graphed as mean ± SEM; p value by Student’s t test. (E) TRPV4 agonist GSK101 cannot inhibit glucose starvation-induced AMPK activation in MEFs pre-depleted of the ER pool of Ca^2+^. MEFs were treated as in (D), followed by addition of 50 nM GSK101 for another 15 min. Cells were then lyzed, and p-AMPKα, and p-ACC analyzed. (F) GSK101 inhibits glucose starvation-induced AMPK activation in CaMKK2^−/−^ MEFs. Cells were incubated in glucose-free medium for 2 h, followed by addition of 50 nM GSK101 for another 15 min, and p-AMPKα, and p-ACC were analyzed. (G) TRPV agonists are unable to evoke CaMKK2 signaling. 2-h glucose-starved MEFs were treated with 50 nM GSK101, 100 nM capsaicin, 0.7 μM RN, 100 μM carvacrol, 200 μM 2-APB, or 1 μM ionomycin for 15 min and then lyzed. The activity of CaMKK2 was determined by western blotting analysis of the phosphorylation of CaMKI. (H and I) Activity of v-ATPase in glucose-starved lysosomes can be restored by low concentration of Ca^2+^*in vitro*. Lysosomes purified from glucose-starved MEFs were incubated with 0.5 μM CaCl_2_ and 1 μg of FLAG-TRPV4 (expressed and purified in HEK293T cells, followed by elution with FLAG epitope peptide). The activity of v-ATPase was determined by its rate to hydrolyze ATP (I) and to transport protons (H). Results were normalized to the group without CaCl_2_ added, and are graphed as mean ± SEM; n = 3 for each condition, p value by Student’s t test. Experiments in this figure were performed three times except those in (F) and (G) twice. See also Figure S5.

As TRPVs are cation channels, we addressed whether Ca^2+^ released by TRPVs exert effects on the lysosomal AMPK pathway. Since TRPV1–4 redundantly regulate AMPK, we chose TRPV4 as a representative example. Re-introduction into TRPV-QKO MEFs of D672A or D682A mutants of TRPV4, which are defective (also confirmed as in Figures S5A and S5B) in channel function (Owsianik et al., 2006, Voets et al., 2002), led to constitutive AMPK activation (Figure 3A). Addition of BAPTA-AM, a cell-permeable calcium chelator (Tsien, 1980), was also sufficient to trigger AMPK activation through the lysosomal pathway both *in vivo* and *in vitro* (Figures 3B–3E and S5C). BAPTA-AM also blocked the effects of channel agonists on the lysosomal translocation of AXIN (Figure 3F). The calcium chelator EGTA-AM, which binds Ca^2+^ more slowly than BAPTA-AM (Stern, 1992, Tsien, 1980), did not trigger the lysosomal pathway (Figure 3H), suggesting that Ca^2+^ release from TRPVs that suppresses AMPK activation in normal glucose was local and transient. This agrees with results that glucose starvation did not cause changes in bulk Ca^2+^ concentrations, as recorded by cytosol-localized Fluo-3 (Figure 4A).

We then used imaging techniques to determine if TRPV4 has co-localization with the lysosomal marker LAMP2. We indeed observed juxtaposition of TRPV4 with LAMP2, as revealed by immunofluorescent staining using confocal microscopy (Figure S5D). Imaging by structured illumination microscopy (SIM) also showed that 17.17% ± 5.7% of TRPV4 puncta (2,337 of 14,160, counted in 11 cells) were juxtaposed with LAMP2 (Figure S5F, validated in Figure S5E). We also performed Stochastic Optical Reconstruction Microscopy (STORM) to study the organization of TRPV4-lysosomal foci *in vivo* at the nanometer scale, and the results clearly showed that a portion of TRPV4 is located in apposition to LAMP2 (Figure 4B). To confirm the Ca^2+^ levels in the immediate vicinity of lysosomes, GCaMP6s was fused to the cytosol-facing C terminus of the lysosomal marker LAMP2 (LAMP2-GCaMP6s, Figure 4C, validated in Figure S5G). We found that glucose starvation strongly blunted the fluorescent signal, in line with the majority of LAMP2 puncta (1,621 of 1,935 or 87.44% ± 5.6%, counted in 11 cells from SIM images) being juxtaposed with TRPV4. These findings are also consistent with the reported observation that over 95% of lysosomes are in apposition to ER via ER-lysosome contact sites (Rowland et al., 2014). It is therefore reasonable to suggest that the Ca^2+^ ions released by TRPV4 comes from the ER Ca^2+^ pool. Indeed, no changes in TRPV4-GCaMP6s signals were detected in MEFs treated with thapsigargin, a drug that depletes ER calcium stores (Figures 4D, left panel, and S5H). In comparison, depletion of extracellular Ca^2+^ had no effect on TRPV4-GCaMP6s intensity during glucose starvation or under TRPV4-specific agonist GSK101 stimulation (Figures 4D, right panel, and S5I). As an additional control, GSK101 could still increase signals of Fura-2 in MEFs with extracellular Ca^2+^ depleted, while knockout of *TRPV4* rendered GSK101 ineffective (Figure S5J). Moreover, GSK101 failed to inhibit glucose-starvation-induced AMPK activation after the ER pool of Ca^2+^ had been depleted by thapsigargin (Figure 4E).

It is well established that increases in bulk cytosolic Ca^2+^ activate AMPK via CaMKK2, unlike the localized pool of Ca^2+^ released from TRPV channels, which inhibits AMPK activation in normal glucose. We previously showed that the Ca^2+^ ionophore A23187 readily activates AMPK in AXIN^−⁄−^ and LAMTOR1^−⁄−^ MEFs via CaMKK2 (Zhang et al., 2014), distinct from the lysosomal pathway that requires AXIN and LAMTOR1 and is independent of CaMKK2 (Zhang et al., 2017). Similarly, TRPV agonists failed to evoke CaMKK2 signaling, as monitored by phosphorylation of CaMKI, another substrate of CaMKK2 (Figure 4G). In addition, in glucose-starved CaMKK2^−⁄−^ MEFs, activation of AMPK was fully suppressed by the TRPV4 agonist GSK101 (Figure 4F), and AMG-9810 still activated AMPK in CaMKK2^−⁄−^ MEFs (Figure S4G). Furthermore, while EGTA-AM could not ablate the suppression of glucose starvation-induced AMPK activation by the agonist GSK101, it suppressed CaMKK2-dependent AMPK activation by ionomycin (Figures S5K–S5N).

Finally, we attempted to reconstitute v-ATPase regulation by re-introducing TRPV4 and Ca^2+^ to purified lysosomes. Addition of TRPV4 alone inhibited the v-ATPase activity, co-addition of Ca^2+^ relieved the inhibitory effect (Figures 4H and 4I), recapitulating that local Ca^2+^ is required to maintain v-ATPase activity.

### Aldolase Inhibits Ca^2+^ Channel Activity of TRPV4 in Low Glucose or FBP

**Figure 5:**
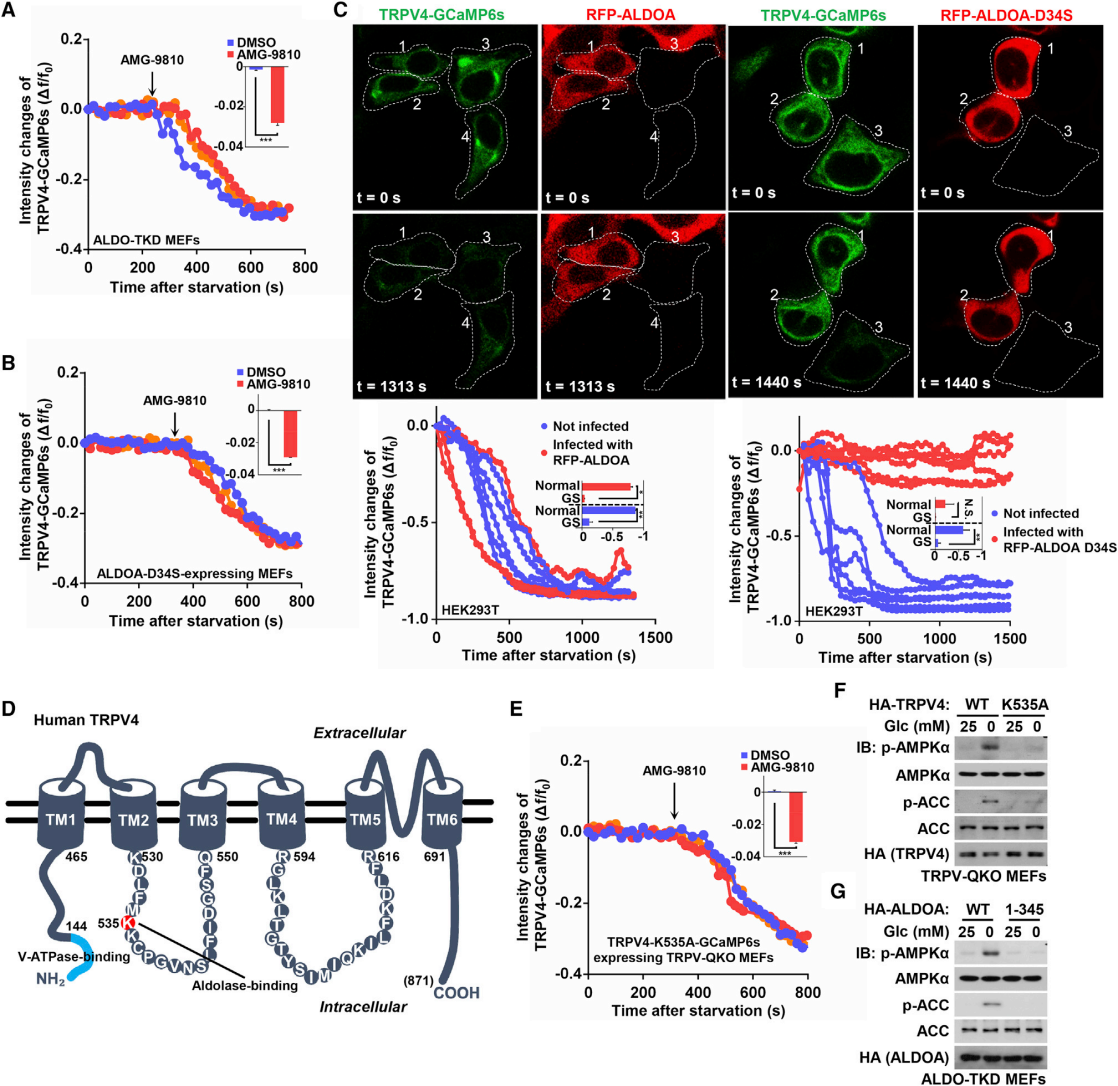
Aldolase Binds to TRPV and Inhibits its Ca^2+^ Channel Activity in Low FBP (A) Aldolases are required for inhibition of TRPV channels. ALDO-TKD MEFs expressing TRPV4-GCaMP6s were incubated in glucose-free DMEM at 37°C. After 2 min in the live-cell incubation chamber, the images were captured by confocal microscopy at a regular interval. After 250 s of starvation, 5 μM AMG-9810 was added to the medium. The changes of TRPV4 activities were analyzed as described in Figure 2A. n = 3 cells from 2 dishes/experiment, and were graphed as mean ± SEM; p value by Student’s t test. (B and C) The FBP-constantly bound aldolase mutant ALDO-D34S fails to block the activity of TRPV4 in low glucose. MEFs (B) and HEK293T cells (C) expressing TRPV4-GCaMP6s were incubated in glucose-free DMEM at 37°C. After 2 min of incubation in the live-cell incubation chamber, the images were captured by confocal microscopy at a regular interval. For MEFs, after 350 s of starvation, 5 μM AMG-9810 was added to the medium, and the relative fluorescent intensities were analyzed and graphed (n = 3 cells from 3 dishes/experiment) in (B). For HEK293T cells expressing HA-ALDOA or its D34S mutant (red, labeled as #1 and #2 in C), representative images at t = 0 s and t = 1313 s (for ALDOA) or 1440 s (for ALDOA-D34S) were shown (upper panel) and the relative fluorescent intensities of GCaMP6s (green) were analyzed and graphed (lower panel, n = 5 cells from 2 dishes/experiment for ALDOA, and n = 7 cells from 3 dishes/experiment for ALDOA-D34S) in (C). Statistical analysis results were shown in mean ± SEM; p value by Student’s t test. (D) Schematic illustration of TRPV4 membrane topology. The K535 residue (in red) of TRPV4 forming the interface for aldolase, and the N-terminal region of aa 1–144 (blue) for binding to v-ATPase (as characterized below) are illustrated. (E) Glucose starvation fails to inhibit the channel activity of TRPV4-K535A that is defective in binding to aldolase. Cells were treated and the activity of TRPV4 was analyzed as in B, except that TRPV4-GCaMP6s-K535A was expressed in TRPV-QKO MEFs (n = 3 cells from 2 dishes/experiment). Statistical analysis data of E were shown as mean ± SEM; p value by Student’s t test. (F) Re-introduction of TRPV4-K535A to TRPV-QKO MEFs suppresses AMPK activation under glucose starvation. TRPV-QKO MEFs stably expressing TRPV4-K535A were regularly cultured or glucose starved for 2 h, followed by analysis of p-AMPKα and p-ACC. (G) The TRPV4-binding-defective, truncated ALDOA (aa 1–345) fails to trigger lysosomal AMPK activation. ALDO-TKD MEFs stably expressing ALDOA 1–345 or WT ALDOA as a control were regularly cultured or glucose starved for 2 h, followed by analysis of p-AMPKα and p-ACC. Experiments were performed for three times. See also Figure S6.

We next determined how low glucose inhibits TRPV channels via aldolase. TRPV4 was tested as a representative. Knockdown of aldolases blocked the effects of glucose starvation in dampening the fluorescent signal obtained with TRPV4-GCaMP6s and LAMP2-GCaMP6s (Figure 5A compare with Figures 2A and S6A), indicating that aldolase is required to block the TRPV4. Importantly, ectopic expression of ALDOA-D34S, which binds FBP constitutively, failed to dampen the signal of TRPV4-GCaMP6s and LAMP2-GCaMP6s (Figures 5B, 5C and S6B), indicating that FBP-occupied aldolase is unable to trigger closure of TRPV4. We conclude that TRPV4 is inhibited by aldolase when it is unoccupied by FBP, i.e., under glucose starvation conditions. Interestingly, signals of TRPV4-GCaMP6s, not only those localized on ER-lysosome contact sites, but also on other parts of the cell, were controlled by aldolase and FBP (see Figure 5C, in which a universally decreased of TRPV4-GCaMP6s signals could be detected). This finding indicates that different pools of TRPV4 can be similarly inhibited by FBP-unoccupied aldolase as aldolase is ubiquitously localized inside cells (apart from those interact with v-ATPase). However, it is important to note that only the portion of TRPV4 localized in the vicinity of lysosome has the chance to modulate v-ATPase on the lysosome. As mentioned earlier, aldolase unoccupied by FBP shows strong interaction with TRPVs (e.g., Figures 1A, 1C, and S1F). We therefore tested whether inhibition of TRPV4 is caused by binding of aldolase. We found that the activity of a K535A mutant of TRPV4, which was unable to bind aldolase (identified by deletion mapping followed by alanine mutagenesis screening, as shown in Figures 5D and S6C–S6F), was still capable of being regulated by its agonist and antagonist, as determined by a TRPV4-GCaMP6s-K535A indicator (Figures 5E and S6G), while AMPK activation was reduced in QKO MEFs in which this mutant was re-expressed (Figure 5F). Similarly, truncated ALDOA (residues 1–345, i.e., lacking the C-terminal 19 amino acids), which was defective in binding to TRPV1 to TRPV4, failed to rescue the AMPK activation when re-introduced into ALDO-TKD MEFs (Figures 5G, S6H, and S6I). These results reinforced the importance of the aldolase-TRPV interaction in blocking channel activity and causing AMPK activation.

### Inactivated TRPV4 Protein after Ca^2+^ Depletion Physically Reconfigures the Association of Aldolase with v-ATPase

**Figure 6:**
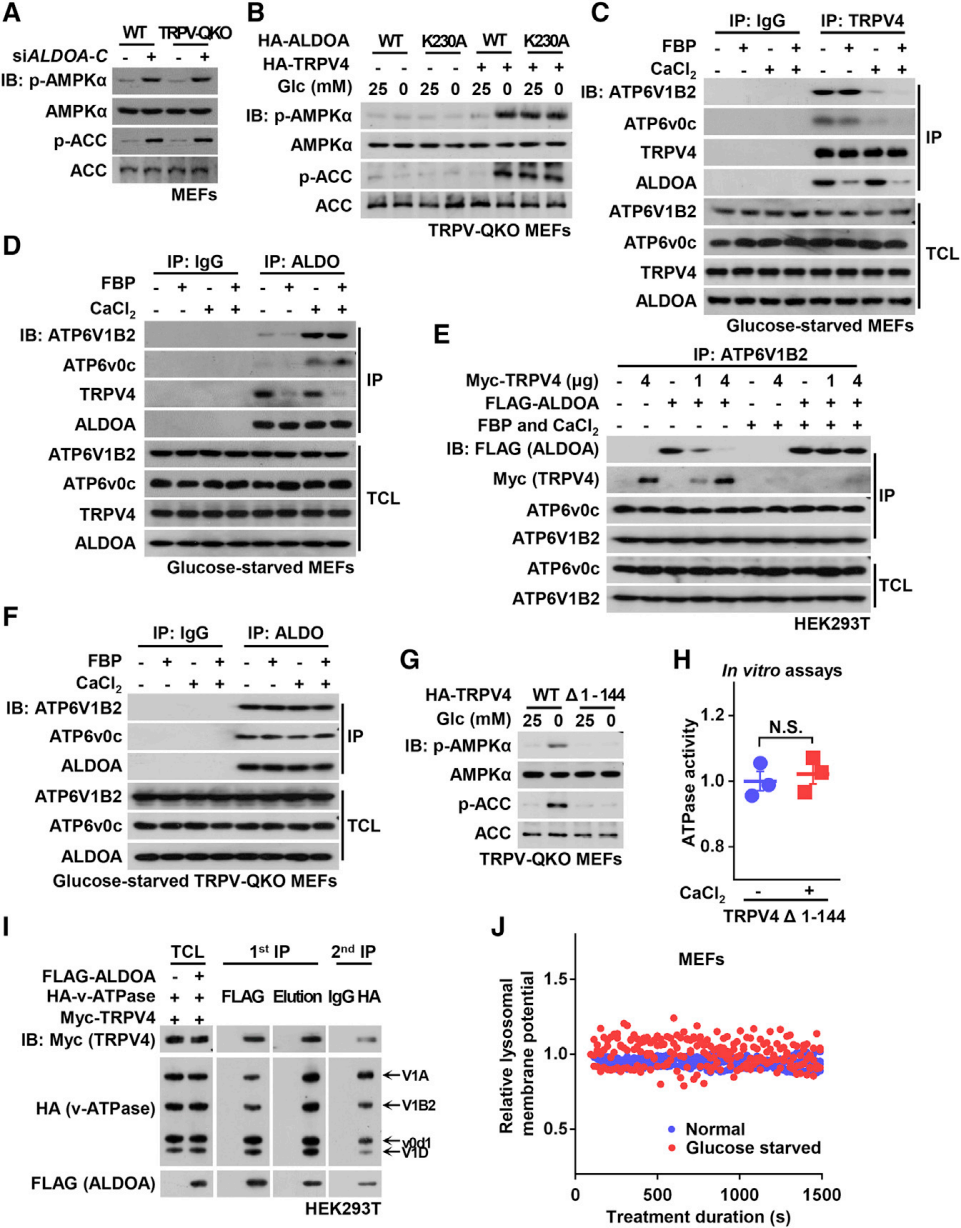
Ca^2+^ Depletion Reconfigures the Association of Aldolase with v-ATPase (A) Knockdown of aldolase bypasses the requirement of TRPV. TRPV-QKO MEFs with knockdown of ALDOA-C were regularly cultured, followed by analysis of the activation of AMPK. (B) Deficiency of TRPV prevents the constitutive activation of AMPK caused by ALDO-K230A. TRPV-QKO MEFs expressing HA-TRPV4 or vector as a control were infected with lentivirus expressing HA-tagged ALDOA or ALDOA-K230A. Cells were regularly cultured, or incubated in glucose-free DMEM for 2 h, followed by analysis of p-AMPKα and p-ACC. (C and D) FBP blocks interaction between aldolase and TRPV, while CaCl_2_ prevents interaction between TRPV with v-ATPase. Lysates from glucose-starved MEFs were mixed with 0.5 μM CaCl_2_ and/or 10 μM FBP. Endogenous TRPV4 (C) and aldolase (D) were separately immunoprecipitated, followed by immunoblotting. (E) TRPV4 competes the binding of aldolase with v-ATPase in the absence of Ca^2+^. HEK293T cells were transfected with different combinations of Myc-TRPV4 and FLAG-ALDOA. Cells were then lyzed, and the protein extracts were mixed with CaCl_2_ and FBP, immunoprecipitated with antibody against endogenous ATP6V1B2, and followed by immunoblotting. (F) Knockout of TRPV renders the interaction of ALDOA-v-ATPase constitutive. Experiments were performed as in (D), except that lysates were purified from glucose-starved TRPV-QKO MEFs. (G) TRPV truncation-mutant defective in binding to v-ATPase fails to trigger lysosomal AMPK activation. TRPV-QKO MEFs stably expressing HA-tagged Δ1-144-TRPV4 mutant (unable to bind to v-ATPase, as determined in Figure S7A), were regularly cultured or glucose starved for 2 h, followed by analysis of p-AMPKα and p-ACC. (H) The mutant Δ1-144-TRPV4 fails to block v-ATPase activity. Lysosomes purified from glucose-starved MEFs were incubated with 0.5 μM CaCl_2_ and 1 μg of Δ1–144 mutant of TRPV4. The rate of v-ATPase to hydrolyze ATP was determined. Results were normalized to the group without CaCl_2_ added, and are graphed as mean ± SEM; n = 3 for each condition, p value by Student’s t test. (I) Two-step coIP shows the coexistence of aldolase, TRPV and v-ATPase (represented by its V1A, V1B2, v0d1, and V1D subunits) in the same complex under glucose starvation. Complex formations between FLAG-ALDOA, Myc-TRPV4 and HA-v-ATPase in glucose-starved HEK293T cells were monitored by two-step coIP followed by immunoblotting. (J) FRET-imaging showing that the lysosomal membrane potential was unchanged under glucose starvation. MEFs were cultured in the DMEM containing glucose or not. The lysosomal membrane potential was determined by the relative cFRET intensity generated from DiBAC_4_(3)-PE-conjugated Rhodamine pair. n = 4 (normal) and n = 3 (glucose starved) cells from 2 dishes/experiment. Experiments in (G) and (J) were performed twice and others three times. See also Figure S7.

We next analyzed how the interplay between aldolase and TRPV affects the v-ATPase and AMPK activation. It is well established that aldolase directly interacts with various subunits of the v-ATPase, and is required for its activity (Lu et al., 2007, Lu et al., 2004). Indeed, knockdown of aldolase directly activates AMPK in both WT and TRPV-QKO MEFs (Figure 6A). It thus appears that aldolase unoccupied by FBP is somehow dissociated from the v-ATPase, driving the v-ATPase to undergo changes that lead to AMPK activation instead. However, when we re-introduced the K230A mutant of aldolase, which cannot form the initial Schiff base with the substrate FBP and thus causes constitutive AMPK activation (Morris and Tolan, 1993, Zhang et al., 2017), AMPK was not activated in the TRPV-QKO MEFs (Figure 6B). Therefore, the absence of FBP from aldolase is not sufficient to cause AMPK activation, but it also requires the physical presence of inactivated TRPVs, such as TRPV4, for re-configuring the aldolase-v-ATPase interaction, eliciting the ultimate conformational change of v-ATPase for AMPK activation. Indeed, we found that TRPV4 interacts with v-ATPase in glucose-starved cells (Figure 6C). Addition of both FBP and CaCl_2_ (mimicking the effect of normal glucose) to lysates prepared from glucose-starved cells strengthened the association between ALDOA and v-ATPase, along with attenuation of the TRPV4:v-ATPase interaction (Figures 6C and 6D). These results imply that the association of aldolase or TRPV4 with v-ATPase may be mutually exclusive. Consistent with this, increased expression of TRPV4 disrupted the interaction between ALDOA and v-ATPase, accompanied by an increase of association between TRPV4 and the v-ATPase, but this was not observed in normal glucose (Figure 6E). Importantly, knockout of TRPV1–4 led to a constant interaction between ALDOA and v-ATPase even in the absence of FBP and CaCl_2_ (Figure 6F). Moreover, the truncation-mutant TRPV4-Δ1-144, which is defective in binding to the v-ATPase, failed to mediate AMPK activation upon glucose starvation, and did not inhibit the activity of v-ATPase based on *in vitro* reconstitution experiments (Figures 6G, 6H, and S7A). These observations, together with those showing the requirement of TRPV activity in lysosomal AMPK activation (Figure 2), point to the same conclusion, i.e. that TRPV1–4 are required both physically and functionally for lysosomal AMPK activation in response to low glucose.

We next dissected the roles of FBP and Ca^2+^ in the interaction between aldolase and v-ATPase by adding them singly to lysates from starved cells. FBP decreased the interaction between ALDOA and TRPV4 (Figures 6C and 6D). By contrast, incubation with CaCl_2_ prevented interaction between TRPV4 and v-ATPase, restoring the interaction between ALDOA and v-ATPase (Figures 6C and 6D), consistent with Ca^2+^ being able to activate v-ATPase *in vitro*, as shown in Figures 4H and 4I. Importantly, ALDOA interacts with TRPV4 as long as FBP is low, regardless of Ca^2+^ concentrations (Figures 6C and 6D). This indicates that the binding of FBP-unoccupied aldolase to TRPV4, prior to the change in Ca^2+^, is the priming step that triggers the glucose-sensing cascade. Subsequently, the reduced level of Ca^2+^ enhances the interaction of TRPV4 with v-ATPase, re-configuring the aldolase:v-ATPase complex into a ternary aldolase:TRPV4:v-ATPase “sandwich”. The existence of an aldolase:TRPV4:v-ATPase complex was further confirmed by a two-step co-immunoprecipitation assay, showing that all three components were detected in the final immunoprecipitates generated from glucose-starved cells (Figure 6I).

The v-ATPase is an electrogenic pump, and its activity in proton transport is maintained both by ATP hydrolysis (directly providing energy for proton transport against an electrochemical gradient) and flux of counterions (dissipating the electrical potential across the lysosomal membrane generated by proton accumulation) (Nishi and Forgac, 2002). We thus determined whether the lysosomal counterion flux was suppressed under glucose starvation conditions, by directly measuring the lysosomal membrane potential at different time points of glucose starvation through an *in vivo* FRET-based method (Koivusalo et al., 2011). The result showed that the lysosomal membrane potential hardly changed under glucose starvation (Figure 6J, validated in Figure S7B). This suggests that the inhibition of v-ATPase during glucose deprivation is not caused by restraint on proton accumulation, or a defect in counterion conductance. Combining the observation that the V1 and v0 domains of v-ATPase were still present in purified lysosomes from cells starved of glucose for 4 h (Figure S7C), we conclude that aldolase and the v-ATPase are not dissociated in low glucose, as has been reported in the yeast system (Lu et al., 2004).

### Inhibition of TRPV1–4 Increases Physical Fitness in Aged Mice

**Figure 7:**
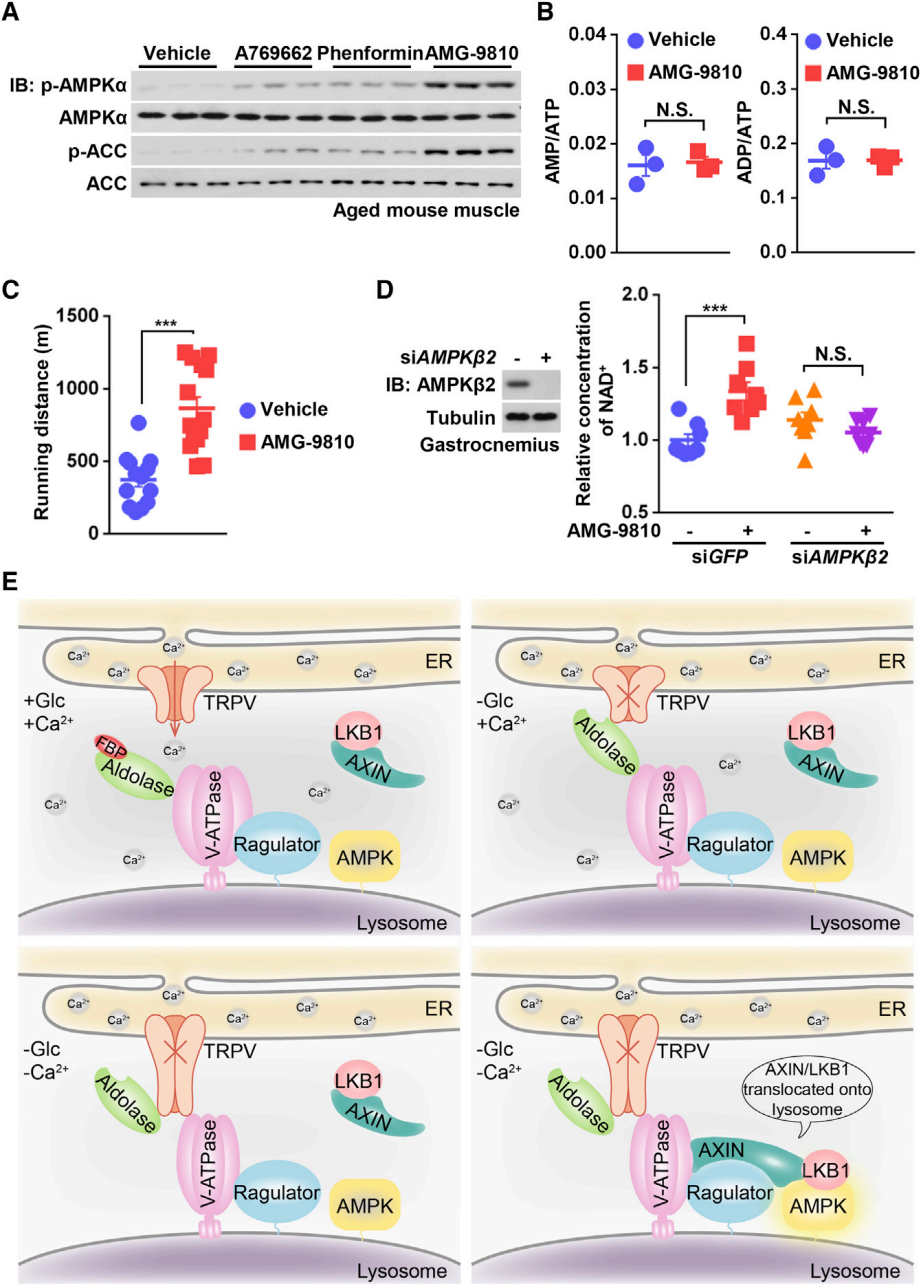
Models for the Roles of TRPV in Glucose Sensing and AMPK Activation (A) Inhibition of TRPV efficiently activates AMPK in muscle from aged mice. Mice at 1.5-year-old were intraperitoneally injected with vehicle (10% (w/v) Kolliphor), 30 mg/kg of A769662, 150 mg/kg of phenformin, or 20 mg/kg of TRPV inhibitor AMG-9810 (all formulated in the vehicle). 1 h after injection, muscles were excised and homogenized, followed by analysis of p-AMPKα and p-ACC levels. (B) AMG-9810 has no effect on AMP:ATP and ADP:ATP ratios in muscle. Mice (1.5-year-old) were treated as in (A), and adenylate nucleotide ratios in muscle tissues were measured by CE-MS. Results are mean ± SD; p value by Student’s t test, n = 3. (C) Inhibition of TRPV robustly increases the running capacity of aged mice. Mice (1.5-year-old) were intraperitoneally injected with 20 mg/kg AMG-9810 daily for two months. The distance (in meters) on the treadmill before exhaustion was graphed. Results are mean ± SEM; p value by Student’s t test, n = 13 for vehicle group and n = 14 for AMG-9810 group. (D) Inhibition of TRPV increases the NAD^+^ levels in an AMPK-dependent manner. Mice (1.5-year-old) were injected with AAV-carried siRNAs against *AMPKβ2*. After 4 weeks of injection, mice were intraperitoneally injected with 20 mg/kg AMG-9810 daily for another three weeks, and the muscular NAD^+^ levels were measured by HPLC-MS. Results are mean ± SEM; p value by ANOVA, n = 8 for each group. (E) Simplified models depicting how absence of glucose/FBP is sensed and relayed from aldolase to TRPVs (TRPV1–4 characterized in MEFs), to v-ATPase, and then to the formation of the AXIN-based lysosomal complex for AMPK activation. When glucose (Glc) supply is abundant, the FBP-occupied aldolase is associated with v-ATPase as an integral component for maintaining its activity, and the TRPV channels release Ca^2+^ locally to the ER-lysosome contact site (upper left panel). In low glucose, FBP-unoccupied aldolase interacts with and inhibits the juxtaposed TRPV on the ER (upper right panel). After the local Ca^2+^ dissipates, TRPV becomes accessible to v-ATPase, thereby re-configuring aldolase:v-ATPase to “sandwich-like” aldolase:TRPV:v-ATPase (lower left panel). Subsequently, v-ATPase is inhibited, and allows AXIN/LKB1 to bind (Zhang et al., 2014), leading to the formation of the AXIN-based AMPK-activating complex for AMPK activation by LKB1 (lower right panel). Experiments in this figure were performed twice. See also Figure S7.

Finally, we tried to explore potential pharmacological effects of TRPVs as regulators of AMPK. The age-related metabolic decline and reduced fitness result in decreased exercise capacity, which has been recognized as a strong predictor of morbidity and mortality in humans (Paffenbarger et al., 1993, Sandvik et al., 1993). Activation of AMPK is well known as a way to improve physical fitness by promoting fatigue resistance and the malleability of muscle (Bujak et al., 2015, Narkar et al., 2008, Steinberg and Jørgensen, 2007), and in fact has been proposed as a promising way to retard aging-associated syndromes (Burkewitz et al., 2014). Unfortunately, AMPK in aged muscle is difficult to be activated by exercise or even pharmacological drugs such as AICAR (Ljubicic and Hood, 2009, Park et al., 2017, Reznick et al., 2007). We thus tested the effects of AMG-9810, an inhibitor to TRPV1–4 that activates AMPK, in 1.5-year-old mice. It was found that administration of the drug activated AMPK in the mice, without altering the adenylate ratios (Figures 7A and 7B). Importantly, AMG-9810 caused much greater AMPK activation in aged muscles compared with A-769662 and phenformin, two classical activators of AMPK (Figure 7A). Strikingly, AMG-9810, after two months of administration, doubled the running capacity of the aged mice (Figure 7C). As NAD^+^ has been shown to be increased by AMPK (Cantó et al., 2009), and is regarded as a symbol of physical fitness including running capacity (Zhang et al., 2016), we measured the levels of NAD^+^, and found that AMG-9810 indeed elevated levels of NAD^+^ (Figure S7D). Importantly, this effect was impaired when *AMPKβ2*, a muscle-specific isoform of AMPK, was knocked down (Figure 7D). Taken together, these results indicate that inhibition of TRPV1–4 provide a way to overcome the difficulty to activate AMPK in aged animals.

## Discussion

In the current study, we have unveiled a missing link in glucose sensing, specifically between the lack of FBP-binding to aldolase and the formation of the lysosomal AMPK-activation complex. When glucose supply is abundant, FBP-occupied aldolase binds to the v-ATPase and acts as an integral component to maintain its activity. In low glucose, FBP-unoccupied aldolase interacts with juxtaposed TRPV1–4 channels to suppress their channel activity. As a result, the local Ca^2+^ concentration decreases at the ER-lysosome contact site, which in turn renders TRPV1–4 accessible to the v-ATPase, generating a ternary complex between aldolase, TRPV and the v-ATPase. The v-ATPase is thus inhibited, promoting the translocation of the AXIN:LKB1 complex to the lysosome and leading to AMPK activation (Figure 7E). A conceivable advantage of inserting another step between aldolase and the v-ATPase is that TRPV channels, along with fluctuations in their released calcium ions, act as buffers or dampers that prevent the activity of v-ATPase from oscillating too rapidly. Even when bound to actin filaments that inhibit the enzyme (Hu et al., 2016), aldolase exhibits a high rate in converting FBP to phosphotrioses, undergoes rapid cycles between FBP-bound and unbound states. This might lead to severe oscillations in v-ATPase activity if the latter was directly controlled by the availability of FBP to aldolase. The localized release of Ca^2+^, which would be retrieved by various nearby Ca^2+^ transporters and pumps or be lost by diffusion into the cytosolic space nearby, is tightly clamped and thus remains at a relatively constant level, preventing TRPV1–4 from interacting with v-ATPase (Mulier et al., 2017, Nita et al., 2016). However, as glucose availability drops, a greater percentage of aldolase would become unoccupied by FBP and more TRPV1–4 channels would be inhibited, leading to a decrease of the local Ca^2+^ concentration. There might be a threshold below which the local Ca^2+^ concentration must drop to allow the v-ATPase to interact with TRPV1–4, thus triggering interaction of AXIN:LKB1 with the lysosome and AMPK activation. Therefore, the involvement of TRPV/Ca^2+^ may buffer oscillations in FBP, like an electric capacitor that filters out fluctuations when attached to an electronic circuit, and provides a more graded regulation of v-ATPase (Figure S7E). With the involvement of TRPV1–4 channels, our work has successfully reconstituted the effects of glucose availability on the v-ATPase, thus resolving a long-standing question in this field (M. Kane, 2012). Of note, a recent study showed that combined starvation of glucose and serum might even increases acidity of newly generated lysosomes before increases of autophagic fluxes (McGuire and Forgac, 2018).

Our study may also suggest a new type of TRPV1–4 activation. Traditionally, TRPV1–4 are known to be stimulated by physical and chemical signals. Here, we found that the TRPV1–4 are constitutively active, by default, so long as glucose is present and no aldolase is in contact with the channels. It is also noteworthy that different concentrations of TRPV agonists might yield differing results in the context of AMPK activation. For example, it has been reported that capsaicin results in AMPK activation in a Ca^2+^- and CaMKK2-dependent manner (Kim et al., 2013). However, the concentrations of capsaicin used in those studies (>10 μM) were much higher than that used here (100 nM), and may have caused a bulk, global increase in Ca^2+^ concentration that might also be blocked by EGTA-AM (Ching et al., 2012, Ying et al., 2013). Thus, excessively high concentrations of capsaicin may cause activation of AMPK via a mechanism different from that studied here.

Another interesting issue is the reciprocal regulation of AMPK and mTORC1, as glucose-sensing and amino acid-sensing on the lysosome share use of the same v-ATPase:Ragulator complex (Zhang et al., 2014). One unanswered question is why amino acid starvation does not activate AMPK, while glucose starvation activates AMPK and concomitantly inhibits mTORC1. We show here that amino acid starvation does not affect the activity of TRPV4 (Figure S7F). Furthermore, TRPV agonists cannot restore lysosomal localization or activity of mTORC1 under conditions of amino acid starvation, although they can restore v-ATPase activity and hence suppress AMPK activation in low glucose (Figures S7G and S7H). It therefore appears that distinct accessory factors affect the v-ATPase:Ragulator complex in different ways. Moreover, our finding of TRPV as an upstream modulator of AMPK may provide new avenues for identifying drugs that treat related metabolic diseases, and improve physical fitness in aged people.

### Limitations of Study

In this study, we have shown that in MEFs, as well as in the mouse liver, TRPV channels function redundantly upstream of the v-ATPase and relay sensing of low glucose/FBP by aldolase to AMPK activation. Since MEFs and mouse liver are found to express only TRPV1–4, it therefore remains to be determined if the remaining two members (TRPV5 and TRPV6) play the same role as do the members 1–4 in different tissues. It is also worthy investigating whether there are other factors augmenting the tethering between the ER and lysosome and participating in AMPK regulation.

Through utilizing the GCaMP6s-TRPV4 indicator and different dyes for Ca^2+^ imaging in different genetic backgrounds, we demonstrated that TRPVs could be inhibited by the FBP-unoccupied aldolase inside cells. Limitation of current patch clamping techniques has hindered a direct recording of the inhibition of TRPVs by the FBP-unoccupied aldolase in low glucose. In addition, as the concentrations of Ca^2+^at the ER-lysosome contact fluctuate along with the variation of cellular glucose levels, it is formally possible that its decrease or increase may have accompanying roles in modulating other proteins and hence physiological roles beyond the regulation of AMPK activity.

## STAR★Methods

### Key Resources Table

**Table:** 

REAGENT or RESOURCE	SOURCE	IDENTIFIER
**Antibodies**		

Rabbit polyclonal antibody against LAMTOR1	Zhang et al., 2014	N/A
Rabbit polyclonal antibody against mouse aldolase	This paper	N/A
Rabbit anti-phospho-AMPKα-T172 antibody	Cell Signaling Technology	cat. #2535; RRID: AB_331250
Rabbit anti-AMPKα antibody	Cell Signaling Technology	cat. #2532; RRID: AB_330331
Rabbit anti-AMPKβ2 antibody	Cell Signaling Technology	cat. #4148; RRID: AB_560862
Rabbit anti-phospho-ACC-Ser79 antibody	Cell Signaling Technology	cat. #3661; RRID: AB_330337
Rabbit anti-ACC antibody	Cell Signaling Technology	cat. #3662; RRID: AB_2219400
Rabbit anti-LKB1 antibody	Cell Signaling Technology	cat. #3047; RRID: AB_2198327
Rabbit anti-AXIN1 antibody	Cell Signaling Technology	cat. #2074; RRID: AB_2062419
Rabbit anti-ALDOA antibody	Cell Signaling Technology	cat. #8060; RRID: AB_2797635
Rabbit anti-β-tubulin antibody	Cell Signaling Technology	cat. #2128; RRID: AB_823664
Mouse anti-Myc-tag antibody	Cell Signaling Technology	cat. #2276; RRID: AB_331783
Mouse anti-phospho-p70S6K-T389 antibody	Cell Signaling Technology	cat. #9234; RRID: AB_2269803
Mouse anti-p70S6K antibody	Cell Signaling Technology	cat. #9202; RRID: AB_331676
Rabbit anti-mTOR antibody	Cell Signaling Technology	cat. #2983; RRID: AB_2105622
Rabbit anti-T7 tag antibody	Cell Signaling Technology	cat. #13246; RRID: AB_2798161
Rabbit anti-HA tag antibody	Cell Signaling Technology	cat. #3724; RRID: AB_1549585
Rabbit anti-PDI antibody	Cell Signaling Technology	cat. #3501; RRID: AB_2156433
Rabbit anti-clathrin antibody	Cell Signaling Technology	cat. #4796; RRID: AB_10828486
Rabbit anti-N-cadherin antibody	Cell Signaling Technology	cat. #13116; RRID: AB_2687616
HRP-conjugated mouse anti-rabbit IgG antibody	Cell Signaling Technology	cat. #5127; RRID: AB_10892860
Rabbit anti-TRPV1 antibody	Alomone Labs	cat. ACC-030; RRID: AB_2313819
Rabbit anti-TRPV4 antibody	Alomone Labs	cat. ACC-034; RRID: AB_2040264
Rabbit anti-calreticulin antibody	Proteintech	cat. 27298–1-AP;
Rabbit anti-ATP6V1B2 antibody	Abcam	cat. ab73404; RRID: AB_1924799
Rabbit anti-ATP6V1B1 + ATP6V1B2 antibody	Abcam	cat. ab200839;
Rabbit anti-CaMKI antibody	Abcam	cat. ab68234; RRID: AB_1140889
Rabbit anti-phospho-CaMKI-T177 antibody	Abcam	cat. ab62215; RRID: AB_940775
Mouse anti-ATP6v0d1 antibody	Abcam	cat. ab56441; RRID: AB_940402
Rat anti-LAMP2 antibody	Abcam	cat. ab13524; RRID: AB_369111
Rabbit anti-ATP6v0c antibody	Novus Biologicals	cat. NBP1-59654; RRID: AB_11004830
Anti-flag® M2 affinity gel	Sigma	cat. A2220; RRID: AB_10063035
Atto 488 goat anti-rabbit IgG antibody	Sigma	cat. 18772; RRID: AB_1137637
Goat anti-AXIN antibody	Santa Cruz Biotechnology	cat. sc-8567; RRID: AB_2227789
Goat anti-TRPV4 antibody	Santa Cruz Biotechnology	cat. sc-47527; RRID: AB_2256617
Mouse anti-HA antibody	Santa Cruz Biotechnology	cat. sc-7392; RRID: AB_627809
Mouse anti-goat IgG-HRP antibody	Santa Cruz Biotechnology	cat. sc-2354; RRID: AB_628490
HRP-conjugated goat anti-mouse IgG antibody	Jackson ImmunoResearch	cat. 115-035-003; RRID: AB_10015289
HRP-conjugated goat anti-rabbit IgG antibody	Jackson ImmunoResearch	cat. 111-035-003; RRID: AB_2313567
Alexa Fluor 405 goat anti-rabbit IgG antibody	Molecular Probes	cat. A31556; RRID: AB_221605
Alexa Fluor 488 donkey anti-goat IgG antibody	Molecular Probes	cat. A11055; RRID: AB_2534102
Alexa Fluor 488 donkey anti-mouse IgG antibody	Molecular Probes	cat. A21202; RRID: AB_141607
Alexa Fluor 488 donkey anti-rabbit IgG antibody	Molecular Probes	cat. A21206; RRID: AB_2535792
Alexa Fluor 568 donkey anti-mouse IgG antibody	Molecular Probes	cat. A10037; RRID: AB_2534013
Alexa Fluor 568 donkey anti-goat IgG antibody	Molecular Probes	cat. A11057; RRID: AB_142581
Alexa Fluor 594 donkey anti-rat IgG antibody	Molecular Probes	cat. A21209; RRID: AB_2535795
Alexa Fluor 594 donkey anti-rabbit IgG antibody	Molecular Probes	cat. A21207; RRID: AB_141637
Alexa Fluor 594 donkey anti-goat IgG antibody	Molecular Probes	cat. A11058; RRID: AB_2534105
Alexa Fluor 568 goat anti-rat IgG antibody	Molecular Probes	cat. A11077; RRID: AB_2534121
Alexa-Fluor 647 donkey anti-mouse IgG antibody	Molecular Probes	cat. A31571; RRID: AB_162542

**Bacterial and Virus Strains**		

OP50	the Caenorhabditis Genetics Center (University of Minnesota)	N/A
BL21	Invitrogen	cat. C609601
Cre Recombinase Adenovirus	Vector Biolabs	cat. 1045

**Chemicals, Peptides, and Recombinant Proteins**		

HEPES	Gibco	cat. 21063
Polyethylenimine	Polysciences	cat. #23966
Glucose	Sigma	cat. G7021
CaCl_2_	Sigma	cat. C5670
MnCl_2_	Sigma	cat. 63535
phosphoenolpyruvate	Sigma	cat. P7002
NADH	Sigma	cat. N8129
pyruvate kinase	Sigma	cat. P9136
lactate dehydrogenase	Sigma	cat. SAE0049
FITC-dextran	Sigma	cat. FD10S
Lysosome Isolation Kit	Sigma	cat. LYSISO1
Endoplasmic Reticulum Isolation Kit	Sigma	cat. ER0100
Capsaicin	Sigma	cat. 360376
GSK101	Sigma	cat. G0798
RN-1747	Sigma	cat. R1033
Carvacrol	Sigma	cat. W224502
Diazoxide	Sigma	cat. D9035
BAPTA-AM	Sigma	cat. O8001
BAPTA	Sigma	cat. A4926
Ionomycin	Sigma	cat. I3909
Oligomycin A	Sigma	cat. 75351
2,4-dinitrophenol	Sigma	cat. 34334
H_2_O_2_	Sigma	cat. 323381
Sorbitol	Sigma	cat. S6021
Phenformin	Sigma	cat. P7045
Octyl β-D-glucopyranoside	Sigma	cat. O8001
Digitonin	Sigma	cat. D141
Saponin	Sigma	cat. S7900
Triton X-100	Sigma	cat. T9284
Concanamycin A	Sigma	cat. C9705
DTT	Sigma	cat. 43815
MEA	Sigma	cat. 30070
Glucose oxidase	Sigma	cat. G2133
Catalase	Sigma	cat. C40
Imidazole	Sigma	cat. I5513
IPTG	Sigma	cat. I6758
L-glutathione reduced	Sigma	cat. G6013
RPMI 1640 Amino Acids Solution	Sigma	cat. R7131
Thapsigargin	Sigma	cat. T9033
2-DG	Sigma	cat. D8375
Kolliphor P188	Sigma	cat. K4894
Acetic acid solution	Sigma	cat. 45754
Ammonium hydroxide solution	Sigma	cat. 338818
Formaldehyde solution	Sigma	cat. F8775
FLAG® Peptide	Sigma	cat. F3290
Nickel Affinity Gel	Sigma	cat. P6611
GSK205	Calbiochem	cat. 616522
AMG-9810	Santa Cruz Biotechnology	cat. sc-201477
EGTA-AM	Santa Cruz Biotechnology	cat. sc-203937
FBP	Santa Cruz Biotechnology	cat. sc-214805
2-APB	Tocris	cat. 1224
A769662	Tocris	cat. 3336
Fura-2-AM	Molecular Probes	cat. F14185
Fura-2	Molecular Probes	cat. F6799
CDFA-SE	Molecular Probes	cat. V12883
Pluronic F-127	Molecular Probes	cat. P3000MP
Fluo-3-AM	Molecular Probes	cat. F14218
Hoechst	Molecular Probes	cat. 33342
LysoSensor Green DND-189	Molecular Probes	cat. L7535
SNARF-5F	Molecular Probes	cat. S23923
CaEGTA stock solution	Molecular Probes	cat. C3008MP
ProLong Diamond Antifade Mountant	Molecular Probes	cat. P36970
ProLong™ Live Antifade Reagent	Molecular Probes	cat. P36975
Ammonium acetate	Millipore	cat. 5330040050
Acetonitrile	Millipore	cat. 1000292500
RPMI 1640 Medium w/o amino acids	US Biological	cat. R8999
TRIzol	Invitrogen	cat. 15596
DEPC-treated water	Invitrogen	cat. AM9922
RQ1 RNase-free DNase	Promega	cat. M6101
GoTaq 1-step RT-qPCR enzyme mix	Promega	cat. A6020
RiboLock RNase inhibitor	Thermo Fisher	cat. E00381
NeutrAvidin Agarose	Thermo Fisher	cat. 29201
EZ-Link™ Sulfo-NHS-SS-Biotin	Thermo Fisher	cat. 21331
M-MLV reverse transcriptase	Takara	cat. 2641A
Doxycycline	Selleckchem	cat. S4163
A-769662	Selleckchem	cat. S2697
Protease inhibitor cocktail	Roche	cat. 04693116001
Dulbecco’s modified Eagle’s medium	Gibco	cat. 11965
Lipofectamine 2000	Invitrogen	cat. 11668-027
Glucose-free DMEM	Gibco	cat. 11966
DMEM without phenol red	Gibco	cat. 21063
MEM Amino Acids Solution	Gibco	cat. 11130-077
MEM Vitamin Solution	Gibco	cat. 11120052
Liver Perfusion Media	Gibco	cat. 17701
Liver Digest Buffer	Gibco	cat. 17703
William’s medium E	Gibco	cat. 32551
Glutamax	Gibco	cat. 35050
Sodium pyruvate	Gibco	cat. 11360
Glutathione Sepharose 4 Fast Flow Gel	GE Healthcare	cat. 17–5132
ACC tide (HMRSSMSGLHLVRRR)	GenScript	N/A
Internal standards 1	Human Metabolome Technologies	cat. H3304-1002
Internal standards 3	Human Metabolome Technologies	cat. H3304-1104

**Critical Commercial Assays**		

GoScript Reverse Transcription System	Promega	Cat. #A5001
GoTaq qPCR Master Mix	Promega	Cat. #A6002

**Deposited Data**		

Mendeley Dataset (Blot quantification data)	This paper	https://data.mendeley.com/datasets/p3zgm58ksw/draft?a=10c923e7-0295-4f9c-ad63-e7e811c789da

**Experimental Models: Cell Lines**		

Human: HEK293T cells	ATCC	CRL3216; RRID: CVCL_0063
Human: AD293 (Adeno-X 293) cells	Clontech	cat. 632271
Mouse: primary hepatocytes from C57BL/6J male mice	The Jackson Laboratory	Stock No: 000664 Black. 6

**Experimental Models: Organisms/Strains**		

Mouse: *LAMTOR1*^F/F^	Zhang et al., 2014	N/A
Mouse: TRPV1^-/-^	The Jackson Laboratory	Dr. David Julius; RRID: MGI:4417977
Mouse: CaMKK2^-/-^	The Jackson Laboratory	Dr. Talal Chatila; RRID: MGI:4941485
Mouse: *LKB1*^F/F^	Frederick National Laboratory for Cancer Research	Dr. Ron DePinho; RRID: MGI:5659884
Caenorhabditis elegans: var. Bristol	the Caenorhabditis Genetics Center (University of Minnesota)	N2; RRID: WB-STRAIN:N2_(ancestral)
Caenorhabditis elegans: *osm-9(ky10)*; *ocr-2(ak47)*; *ocr-1(ak46)*	the Caenorhabditis Genetics Center (University of Minnesota)	FG125; RRID: WB-STRAIN:FG125

**Oligonucleotides**		

siRNA targeting sequence: *mAldoa* (#1): 5’-CCAAGTGGCGCTGTGTGCT-3′	Zhang et al., 2017	N/A
siRNA targeting sequence: *mAldob* (#1): 5’-GCTCTCTGAGCAGATCCAT-3′	Zhang et al., 2017	N/A
siRNA targeting sequence: *mAldoc* (#1): 5’ -GAGTCTAGAGCTTATGTCT-3′	Zhang et al., 2017	N/A
siRNA targeting sequence: *mTrpv2*: 5’-GGTGCTTCAGGGTGGAGGAAG-3’	This paper	N/A
siRNA targeting sequence: *mTrpv3*: 5’-GGAGAACGTCTCCAAAGAAAG-3’	This paper	N/A
siRNA targeting sequence: *mTrpv4*: 5’-GACATCCCTGCACATTGCCAT-3’	This paper	N/A
siRNA targeting sequence: *mAMPKβ2*: 5’-CTCATCTGCAATCAAATGC-3’	This paper	N/A
sgRNA targeting sequence: *mTrpv1*: 5’-GGAGTCGCACCCGGCTTTTT-3’	http://crispr.mit.edu	N/A
sgRNA targeting sequence: *mTrpv1*: 5’-CAGGAGCATCTTCGACGCTG-3’	http://crispr.mit.edu	N/A
sgRNA targeting sequence: *mTrpv2*: 5’-CGGTCACGGTCAAACCGATT-3’	http://crispr.mit.edu	N/A
sgRNA targeting sequence: *mTrpv2*: 5’-GGTACTTGCTGGTCCGGCGC-3’	http://crispr.mit.edu	N/A
sgRNA targeting sequence: *mTrpv3*: 5’-AGTACAACAGGGTTCCCGCC-3’	http://crispr.mit.edu	N/A
sgRNA targeting sequence: *mTrpv3*: 5’-ATCTTCGCGGCTGTGTCCGA-3’	http://crispr.mit.edu	N/A
sgRNA targeting sequence: *mTrpv4*: 5’-TGTCGTTGCGCCCG TTGCTT-3’	http://crispr.mit.edu	N/A
sgRNA targeting sequence: *mTrpv4*: 5’-GTAAGTGCCGTAGTCGAACA-3’	http://crispr.mit.edu	N/A
Primer: 5’-CCGGCTTTTTGGGAAGGGT-3’ and 5’-GAGACAGGTAGGTCCATCCAC-3’ for *mTrpv1*	PrimerBank https://pga.mgh.harvard.edu/primerbank/	N/A
Primer: 5’-GGACCCAAATCGGTTTGACC-3’ and 5’-GCGCAGGTACTCTAGCAGTC-3’ for *mTrpv2*	PrimerBank https://pga.mgh.harvard.edu/primerbank/	N/A
Primer: 5’-ACGGTCACCAAGACCTCTC-3’ and 5’-GACTGTTGGGATTGGATGGGG-3’ for *mTrpv3*	PrimerBank https://pga.mgh.harvard.edu/primerbank/	N/A
Primer: 5’-AAACCTGCGTATGAAGTTCCAG-3’ and 5’-CCGTAGTCGAACAAGGAATCCA -3’ for *mTrpv4*	PrimerBank https://pga.mgh.harvard.edu/primerbank/	N/A
Primer: 5’-TGCTG CTATAATGCTGATGGAG-3’ and 5’- GCACGGACTAGGTTCACATTCT -3’ for *mTrpv5*	PrimerBank https://pga.mgh.harvard.edu/primerbank/	N/A
Primer: 5’-GACCAGACACCTGTAAAGGAAC-3’ and 5’-AGACACAGCACATGGTAAAGC-3’ for *mTrpv6*	PrimerBank https://pga.mgh.harvard.edu/primerbank/	N/A

**Recombinant DNA**		

CMV-GCaMP6s	Addgene	plasmid #40753
LentiCRISPR v2	Addgene	plasmid #52961
pLL3.7 vector	Adegene	plasmid #11795
pcDNA3.3 vector	Thermo Fisher	cat. K830001
pBOBI vector	Miyoshi et al., 1998	N/A
pLVX-IRES vector	Takara	cat. 631849
AAV2 inverted terminal repeat (ITR) vectors pseudo-typed with AAV9 capsid	Bish et al., 2008	N/A
pET-28a	Novagen	cat. 70777
pGEX4T-1	GE Healthcare	cat. 28-9545-49

**Software and Algorithms**		

StepOne software version 2.3	Applied Biosystems	https://www.thermofisher.com
Zen 2012	Zeiss	https://www.zeiss.com
DeltaVision OMX system	GE Healthcare	https://www.gelifesciences.com
NIS Elements software with STORM package version 4.30 build 1053	Nikon	https://www.nikon.com
Qualitative Analysis B.06.00	Agilent	https://www.agilent.com
SPSS Statistics 17.0	IBM	https://www.ibm.com
GraphPad Prism 6	Graphpad	https://www.graphpad.com
Imaris 7.4.0	Bitplane	https://www.bitplane.com
ImageJ	National Institutes of Health	https://imagej.nih.gov/ij/

### Contact for Reagent and Resource Sharing

Requests for reagents and resources should be directed to and will be fulfilled by the Lead Contact, Sheng-Cai Lin (linsc@xmu.edu.cn).

### Experimental Model and Subject Details

#### Mouse Studies

Protocols for all animal experiments were approved by the Institutional Animal Care and the Animal Committee of Xiamen University. Mice were housed with free access to water and standard diet (65% carbohydrate, 11% fat, 24% protein). The light was on from 8 a.m. to 8 p.m. Male littermate controls were used throughout the study.

#### CRISPR Knockout of TRPV1-4

The genes (*mTrpv1*, *mTrpv2*, *mTrpv3*, and *mTrpv4*) were deleted from MEFs using the CRISPR-Cas9 system. Nucleotides were annealed to their complements containing the cloning tag aaac, and inserted into the back-to-back *Bsm*BI restriction sites of lentiCRISPRv2 vector. The four constructs were then separately subjected to lentivirus packaging using HEK293T cells in which cells were transfected with 2-3 μg of DNA in Lipofectamine 2000 transfection reagent per well of a 6-well plate. At 30 hr post transfection, the four kinds of virus were collected and then added in 1:1:1:1 ratio to MEFs (cultured to 15% confluence) for another 72 hr-infection. When cells were approaching to confluency, they were single-cell sorted into 96-well dishes. Clones were expanded and evaluated for knockout status by sequencing.

#### *Caenorhabditis elegans* Studies

Worms were maintained on nematode growth medium (NGM) plate with OP50 as standard food (Brenner, 1974). All worms were cultured at 20°C. The *osm*-*9*(ky10); *ocr*-*2*(ak47) strain was obtained by crossing *osm*-*9*(ky10); *ocr*-*2*(ak47); *ocr*-*1*(ak46) strain (FG125) with N2, and outcrossed 6 times to N2 prior to the experiment. Glucose restriction was performed as described previously (Schulz et al., 2007). Plates used for the treatment of glucose restriction were prepared from the same batch of NGM agar as the control plates.

### Method Details

#### Treadmill Endurance Test

The treadmill endurance test was performed as described previously (Park et al., 2017), with minor modifications. Briefly, test was performed during dark cycle. Mice were trained on Rodent Treadmill NG (UGO basile, cat. 47300) at 10 m/min for 5 min for 2 days. For the treadmill endurance test, the treadmill was set at a 15° incline, and the speed of treadmill was set to increase in a ramp-mode (10 m/min for 10 min followed by an increase to final speed of 18 m/min within 15 min). The test was terminated when mice reached exhaustion, which was defined as staying immobilized after 30 s of electric shocks (2 Hz, 0.5 mA).

#### Packaging and Injection of Adeno-associated Virus

Adeno-associated virus (AAV) was packaged in HEK293T cells following the protocol from Grieger et al. (2006). Briefly, cells used for in-house viral production were maintained in 150-mm dishes. Some 7 μg of pAAV-RC2/9 (AAV2 inverted terminal repeat (ITR) vectors pseudo-typed with AAV9 capsid) plasmid, 21 μg of pAAV-helper plasmid, and 7 μg of pAAV2 plasmid (carrying siRNAs against mouse *TRPV2* to *TRPV4*) were added to 4 mL of DMEM without phenol red (Gibco, cat. 21063), followed by mixing with 175 μL of polyethylenimine (PEI) solution (1 mg/mL, pH 7.5). The mixture was then incubated at room temperature for 20 min, and then added to the dishes. 60 hr post transfection, cells were harvested by scraping and centrifugation. The viral particles were purified from the pellet by an Optiprep gradient as described previously (Grieger et al., 2006). Purified AAV was titered by real-time qPCR (RT-qPCR), and then stored at - 80°C.

AAV was delivered to 6 week old male TRPV1^-/-^ mice intravenously via lateral tail vein injection. For each mouse, 1× 10^11^ particles of virus, adjusted to 200 μL final volume (with PBS, pH7.4), was injected. Four weeks post injection, liver tissues were dissected and analysis for AMPK activation.

#### Plasmids

Point mutations of human ALDOA, mouse TRPV1 and human TRPV4 were performed by a PCR-based site-directed mutagenesis method using PrimeSTAR HS polymerase (Takara). Expression plasmids for various proteins were constructed in the pcDNA3.3 vector for transient transfection, in pBOBI for lentivirus packaging (stable expression), or in pLVX-IRES for doxycycline-inducible expression. PCR products were verified by sequencing (Invitrogen, China). The lentivirus-based vector pLL3.7 was used for expression of siRNA in HEK293T and mouse embryonic fibroblasts (MEFs), and the AAV-based vector pAAV2 for mouse liver.

#### Cell Culture, Transient Transfection and Lentivirus Infection

HEK293T, AD293 (Adeno-X 293) cells and MEFs were maintained in Dulbecco’s modified Eagle’s medium supplemented with 10% fetal bovine serum (FBS), 100 IU penicillin, 100 mg/mL streptomycin at 37°C in a humidified incubator containing 5% CO_2_. PEI at a final concentration of 10 μM was used to transfect HEK293T cells. Total DNA for each plate was adjusted to the same amount by using relevant empty vector. Transfected cells were harvested at 24 hr after transfection. Lentivirus for infection of MEFs was packaged in HEK293T cells by transfection using Lipofectamine 2000. At 30 hr post transfection, medium was collected and added to the cells. The cells were incubated for another 24 hr. Adenovirus was propagated in AD293 cells and purified by cesium chloride density gradient ultracentrifugation. *LAMTOR1*^F/F^, and CaMKK2^-/-^ MEFs were established by introducing SV40 T antigen into primary cultured embryonic cells from a litter of corresponding mice. LAMTOR1^-/-^ MEFs were generated by infecting *LAMTOR1*^F/F^ MEFs with adenoviruses expressing the Cre recombinase for 12 hr. The infected cells were then incubated in the fresh DMEM for another 8 to 10 hr before further treatments. Cells were verified to be free of mycoplasma contamination and authenticated by STR sequencing. ALDO-TKD MEFs were generated and validated as described previously (Zhang et al., 2017). In brief, MEFs carrying doxycycline-inducible expression of ALDOA-C (infected with lentivirus packaged with pLVX-IRES-ALDOA, pLVX-IRES-ALDOB and pLVX-IRES-ALDOC) were cultured in medium containing doxycycline (Dox, 100 ng/mL), and were infected with lentivirus expressing siRNA against *ALDOA*, *ALDOB* and *ALDOC* sequentially, or *GFP* siRNA as a control, followed by incubation in doxycycline-free medium for another 12 hr. For glucose starvation, cells were rinsed twice with PBS, and then incubated in glucose-free DMEM supplemented with 10% FBS and 1 mM sodium pyruvate for desired periods of time at 37°C. For depletion of extracellular Ca^2+^, cells were incubated in a solution containing all components (except CaCl_2_) of DMEM without phenol red (Gibco cat. 21063) and supplemented with sodium pyruvate and 5 mM EGTA for desired time periods at 37°C.

#### Isolation and Culture of Primary Hepatocytes

Primary hepatocytes were isolated from mice with a modified two-step perfusion method using Liver Perfusion Media and Liver Digest Buffer. Cells were plated in collagen-coated 6-well plates in William’s medium E plus 10% FBS, 100 IU penicillin and 100 mg/mL streptomycin. After 4 hr of attachment, the medium was replaced with fresh William’s medium E with 1% BSA for further use.

#### Immunoprecipitation and Immunoblotting

Endogenous LAMTOR1, TRPV4, and aldolase were immunoprecipitated and analyzed as described previously (Zhang et al., 2014) with minor modifications. Briefly, 10 × 15 cm dishes of MEFs (grown to 80% confluence) for IP of LAMTOR1; 8 × 15 cm dishes of MEFs (grown to 80% confluence) for IP of TRPV4 and aldolase for each gel lane were collected and lysed with 750 μL/dish of ice cold ODG buffer [50 mM Tris-HCl, pH 8.0, 50 mM NaCl, 1 mM EDTA (not included in assays involving Ca^2+^ addition), 2% ODG, 5 mM β-mercaptoethanol with protease inhibitor cocktail], followed by sonication and centrifugation at 4°C for 15 min. Cell lysates were incubated with respective antibodies overnight. Overnight protein aggregates were pre-cleared by centrifugation at 20,000 *g* for 10 min, and protein A/G beads (1:250, balanced with ODG buffer) were then added into the lysate/antibody mixture for another 3 hr at 4 °C. The beads were spun and washed with 100 times volume of ODG buffer for 3 times at 4 °C and then mixed with an equal volume of 2× SDS sample buffer and boiled for 10 min before immunoblotting.

For IP of ectopically expressed TRPV or aldolase, FLAG-tagged TRPV and aldolase or HA-tagged aldolase and TRPV were co-transfected into a 10 cm-dish of HEK293T cells. After 24 hr of transfection, cells were collected and lysed in 600 μL of ice cold ODG buffer, followed by sonication and centrifugation at 4°C for 15 min. ANTI-FLAG® M2 Affinity Gel (1:100, balanced in ODG buffer) was added into the lysates and mixed for 4 hr at 4 °C. The beads were washed with 200 times volume of ODG buffer for 3 times at 4 °C. The FLAG tagged TRPV and aldolase were then eluted with 30 μL of FLAG® Peptide (400 μg/mL final concentration) for another 45 min at 4 °C. Some 30 μL of eluent was then collected, mixed with 7.5 μL of 5× SDS buffer, and boiled for 10 min before immunoblotting. In particular, samples containing TRPV2 were not boiled to avoid the formation of insoluble aggregates that would fail to run into the SDS-PAGE.

To analyze the levels of p-AMPKα and p-ACC in MEFs, cells grown to 70-80% confluence in a well of a 6-well dish were lysed with 250 μl of ice cold lysis buffer (20 mM Tris-HCl, pH 7.5, 150 mM NaCl, 1 mM EDTA, 1 mM EGTA, 1% Triton X-100, 2.5 mM sodium pyrophosphate, 1 mM β-glycerolphosphate, with protease inhibitor cocktail). The lysates were then centrifuged at 20,000 *g* for 10 min at 4°C and an equal volume of 2× SDS sample buffer was added into the supernatant. To analyze the levels of p-AMPKα and p-ACC in liver, freshly excised tissue was added with ice-cold lysis buffer (10 μL/mg liver weight), followed by homogenization and centrifugation as described above. The lysates were then mixed with 2× SDS sample buffer and subjected to immunoblotting. To analyze the levels of p-AMPKα and p-ACC in nematodes, about 150 nematodes cultured on the NGM agar plate were collected for each sample. Worms were washed with ice-cold M9 buffer (22.1 mM KH_2_PO_4_, 46.9 mM Na_2_HPO_4_, 85.5 mM NaCl, and 1 mM MgSO_4_) and were lyzed with 150 μL of ice-cold lysis buffer. The lysates were then mixed with 5× SDS sample buffer, followed by homogenization and centrifugation as described above, and then subjected to immunoblotting. Levels of total proteins and phosphorylated proteins were analyzed on separate gels, and representative immunoblots were shown. The band intensities on developed films were quantified using Image J software (National Institutes of Health Freeware).

#### Quantification of TRPV1-6 Expression Levels by Real-time PCR

To isolate total RNA, a 10 cm-dish of MEFs or 10 mg of mouse liver were lysed in 1 mL of TRIzol reagent, followed with addition of 270 μL of chloroform and mixed vigorously. After centrifugation at 12,000 *g* for 15 min at 4°C, 550 μL of upper aqueous layer was transferred to a clean tube. RNA was then precipitated by adding 670 μL of isopropanol, followed with centrifugation at 12,000 *g* for 10 min at 4°C. The pellet was washed with 75% ethanol for 3 times by centrifugation at 7,500 *g* for 5 min, and was dissolved with 30 μL of DEPC-treated water. The concentration of RNA was then determined by a NanoDrop 2000 spectrophotometer (Thermo Scientific). Some 5 μg of RNA was digested with RQ1 DNase (supplemented with RiboLock RNase inhibitor) for 30 min at 37°C, and at 68°C for another 10 min. The digested RNA was mixed with random primers at 70°C for 10 min, and chilled on ice immediately. dNTP and M-MLV reverse transcriptase was then added to the mixture, followed by incubation at 30°C for 10 min, 42°C for 1 hr, and 70°C for 15 min.

The annealing temperature of each pair of primers was first optimized according to the amplification curves, melting curves, and bands on agarose gel of serial pilot reactions (in which a serial annealing temperature was set according to the estimated annealing temperature of each primer pair). Here, all annealing temperatures were set at 60°C. The standard curve used for quantifying the absolute copy number of each TRPV was then generated by using serial dilutions of pcDNA3.3 plasmids containing cDNA of each TRPV (as a template), during which the optimal threshold was set automatically. The correlation coefficient (R^2^) of each standard curve was calculated. Here, all R^2^ values were larger than 0.99. The absolute copy number of each TRPV was then determined according to the standard curves generated from the same assay plate. In experiments described above, GoTaq 1-step RT-qPCR enzyme mix was used for amplifying targets; the StepOnePlus system and StepOne software version 2.3 (Applied Biosystems) were used for performing PCR assays and data processing.

#### Confocal Microscopy

For fixed cell samples, the slides were fixed for 20 min with 4% formaldehyde in phosphate-buffered saline (PBS) at room temperature. The slides were rinsed twice with PBS and cells were permeabilized with 0.05% Triton X-100 in PBS (except for staining AXIN with 0.1% Triton X-100) for 5 min at 4°C. After rinsing twice with PBS, the slides were incubated with primary antibody diluted in PBS overnight at 4°C. Here, goat anti-AXIN, rabbit anti-ATP6V1B1 + ATP6V1B2, anti-mTOR, rabbit anti-T7 tag, mouse anti-ATP6v0d1 and rat anti-LAMP2 were used, as described in the corresponding legend to specific figures. The cells were then rinsed three times with 1 mL of PBS, and then incubated with secondary antibody for 8 hr at room temperature in the dark. The Alexa Fluor 405 goat anti-rabbit IgG, Alexa Fluor 488 donkey anti-goat IgG, Alexa Fluor 488 donkey anti-mouse IgG, Alexa Fluor 488 donkey anti-rabbit IgG, Alexa Fluor 568 donkey anti-mouse IgG, Alexa Fluor 568 donkey anti-goat IgG, Alexa Fluor 594 donkey anti-rat IgG, Alexa Fluor 594 donkey anti-rabbit IgG, and Alexa Fluor 594 donkey anti-goat IgG antibodies were used, as described in the corresponding legends. Cells were washed for another 4 times with 1 mL of PBS, and then mounted on slides using ProLong Diamond Antifade Mountant. Confocal microscopic images were taken on a Zeiss Laser Scanning Microscope (LSM) 780 with a 63× 1.4 NA oil objective.

For detecting the pH of lysosomes, MEFs were grown on 35 mm of glass-bottom dishes, and were cultured to 60–80% confluence. Cells were treated with 1 μM (final concentration) LysoSensor™ Green DND-189 for 1 hr, then washed twice with PBS and incubated in fresh, desired medium for another 30 min. In the meantime, 2 μg/mL (final concentration) Hoechst was added into the medium for staining nucleus before taking images. For detecting the cytosolic pH, MEFs were incubated with 10 μM (final concentration) SNARF-5F in serum-free DMEM for 0.5 hr, washed twice with PBS and then switched to glucose-free DMEM containing 10% FBS. For recording the signals of Fluo-3 in real time, cells were loaded with 5 μM (final concentration) Fluo-3-AM for 30 min, then washed twice with PBS and incubated in fresh, desired medium for another 30 min. Before image taking, ProLong™ Live Antifade Reagent was added to the medium. MEFs expressing GCaMP6s-fused protein were directly imaged after adding ProLong™ Live Antifade Reagent to the medium. During imaging, live cells were kept at 37°C, 5% CO_2_ in a humidified incubation chamber (ZEISS, Incubator PM S1). Images were taken on a Zeiss LSM 780 with a 63× 1.4 NA oil objective at regular intervals. The objective lens was automatically focused at every 30 s.

In experiments described above, fluorescent dye Alexa Fluor 405 and Hoechst were excited with a Diode laser using a 405-nm laser line; Lysosensor, Fluo-3, GCaMP6S, SNARF-5F and Alexa 488 with an Ar gas laser (laser module LGK 7812) using a 488-nm laser line; Alexa 568 with a DP Solid State laser (YLK6120T) using a 561-nm laser line; Alexa 594 with a HeNe gas laser (LGK 7512 PF) using a 594-nm laser line; and Alexa 647 with a HeNe gas laser (LGK 7628-1F) using a 633-nm laser line. The parameters, including ‘PMT voltage’, ‘Offset’, ‘Pinhole’ and ‘Gain’, were kept unchanged between each picture taken. The resolution of image is 1,024 × 1,024 pixels.

For imaging endogenous ER-localized TRPV4, the Semi-intact IF protocol (Du et al., 2016) was used. MEFs were grown on a 35 mm dish (In Vitro Scientific, cat. D35-20-10-N) to 50-60% confluence. Cells were rinsed with PBS once, and treated with Buffer I (25 mM HEPES, pH 7.2, 125 mM potassium acetate, 5 mM magnesium acetate, 1 mM DTT, 1 mg/L glucose and 25 μg/mL digitonin) for 2 min on ice, and then Buffer II (25 mM HEPES, pH 7.2, 125 mM potassium acetate, 5 mM magnesium acetate, 1 mM DTT and 1 mg/L glucose) for another 15 min on ice. The cells were then treated with 4% formaldehyde in PBS at room temperature for 10 min. The slides were rinsed twice with PBS and cells were then permeabilized with 0.05% Triton X-100 in PBS for 5 min at 4°C. After rinsing twice with PBS, the slides were blocked in Block Buffer (10% FBS in PBS, with 0.1% saponin) for 30 min. The slides were washed twice with PBS and incubated with primary antibodies (goat anti-TRPV4 and rat anti-LAMP2) diluted in Block Buffer overnight at 4°C. The cells were then rinsed three times with PBS, and then incubated with secondary antibodies (Alexa Fluor 488 donkey anti-goat IgG and Alexa Fluor 594 donkey anti-rat IgG) for another 8 hr at 4°C in the dark, followed by washing for four times with PBS and then mounted on slides using ProLong Diamond Antifade Mountant. The slides were imaged on a Zeiss Laser Scanning Microscope (LSM) 880 using an AiryScan detector. The fluorescent dye Alexa Fluor 488 was excited with a Diode laser using a 488-nm laser line, and Alexa Fluor 640 with a Diode laser using a 640-nm laser line. Images were processed in AiryScan mode on Zen 2012 software. The resolution of images is 2,312 × 2,312 pixels.

#### 3D-SIM Imaging

MEFs stably expressing HA-TRPV4 were grown in a 35-mm dish (In Vitro Scientific, cat. D35-20-10-N) to 50-60% confluence, and were treated following the Semi-intact IF protocol as described above, except that the cells were incubated in rabbit anti-HA tag and rat anti-LAMP2 primary antibodies, and then with Alexa Fluor 488 donkey anti-rabbit IgG and Alexa Fluor 568 goat anti-rat IgG secondary antibodies. Images were acquired using a UPlanSApochromat 100× 1.4NA, oil immersion objective lens (Olympus) and a back-illuminated Cascade II 512 × 512 electron-multiplying charge-coupled device (EMCCD) camera (Photometrics) on an OMX version 2 system (Applied Precision) equipped with 405-, 488-, 561-, and 647 nm solid-state lasers. Immersion oil of refractive index 1.1515 was used, after being empirically determined to give the most symmetric point spread function. Samples were illuminated by a coherent scrambled laser light source that had passed through a diffraction grating to generate the structured illumination by interference of light orders in the image plane to create a 3D sinusoidal pattern, with lateral stripes approximately 0.2 μm apart. The pattern was shifted laterally through five phases and through three angular rotations of 60° for each Z-section, separated by 0.125 μm, and the range of z-stack was 3 μm. The power of each laser was adjusted to achieve optimal intensities of between 2,000 and 4,000 counts in a raw image of 16-bit dynamic range, at the lowest possible laser power to minimize photo bleaching. Each frame acquisition was separated by a 300-ms pause. Raw images were processed and reconstructed as described previously (Schermelleh et al., 2008). The channels were then aligned in x, y, and rotationally using predetermined shifts as measured using a target lens and the Softworx alignment tool (Applied Precision). The images were further processed in OMX system (Applied Precision).

#### STORM Imaging

MEFs stably expressing HA-TRPV4 and Myc-LAMP2 were cultured in the Lab-Tek™II chambered #1.5 German Coverglass System (NUNC, 155409, 8 Chamber) to 50% confluence, and were treated following the Semi-intact IF protocol as described above, except that the cells were incubated in rabbit anti-HA tag primary antibody and mouse anti-Myc tag primary antibody, and then with the Atto 488 goat anti-rabbit IgG and Alexa-Fluor 647 donkey anti-mouse IgG secondary antibodies. The slides were then fixed with 4% formaldehyde for another 10 min, and washed twice with PBS. The STORM Imaging Buffer with MEA was then prepared according to the manufacturer’s instructions. Briefly, 7 μL of GLOX (14 mg of glucose oxidase, 50 μL catalyze (17 mg/mL), 200 μL Buffer A (10 mM Tris, pH 8.0, and 50 mM NaCl), vortexed to dissolve and cooled on ice) and 70 μL 1 M MEA (77 mg MEA dissolved in 1.0 mL of 0.25 N HCl) were added to 620 μL Buffer B (50 mM Tris, pH 8.0, 10 mM NaCl and 10% (m/v) glucose) in a 1.5 mL Eppendorf tube, followed by gentle vortex. The mixture was then added to each well, and images were taken on an N-STORM (Nikon). The imaging was performed using an inverted microscope system (Ti-E Perfect Focus; Nikon) equipped with a monolithic laser combiner (MLC400, Agilent Technologies) containing solid-state lasers of wavelengths 405 nm, 488 nm, and 561 nm at 50 mW (maximum fiber output power) and a 647-nm laser at 125 mW. After locating a suitable field, a diffraction-limited TIRF image was acquired for reference, followed by a STORM acquisition. The 647-nm laser was then sequentially fired at 100% power to excite all possible fluorophore molecules and photoswitch them into a nonemitting dark state, and then the 488-nm laser. The emitted wavelengths from Alexa Fluor 647 and Atto 488 fluorophores were then sequentially collected by the plan-Apochromat 100×/1.49 TIRF objective (Nikon), filtered by an emission filter set (Nikon TIRF Cube consisting of a TRF89902-EM filter set, Chroma Technology), and detected on an electron-multiplying charge-coupled device camera (Ixon DU-897, Andor Technology). During imaging, 20,000 sequential frames of each channel were acquired. The image acquisition, lateral drift correction, and data processing were performed by using NIS Elements software with STORM package (version 4.30 build 1053, Nikon) as described previously (Dempsey et al., 2011, Jones et al., 2011).

#### Intracellular Calcium Measurement by Fura-2-AM

Briefly, MEFs were cultured to 80-90% confluence in a black, clear-bottom 96-well assay plate (Corning, cat. 3603). Cells were cultured in DMEM without phenol red supplemented with sodium pyruvate, then loaded with 5 μM (final concentration, pre-mixed with the Pluronic F-127 stock solution at a ratio of 1:1) Fura-2-AM for 1 hr. The unloaded Fura-2-AM was then removed by two times of PBS rinsing, and the cells were further incubated in DMEM without phenol red (supplemented with sodium pyruvate) for 0.5 hr. The average concentrations of intracellular calcium, [Ca^2+^]i, were then determined according to the fluorescent intensities of Fura-2 excited at 340 nm (F_λ1_, represents Ca^2+^ bound-state) and 380 nm (F_λ2_, represents Ca^2+^ unbound-state), respectively (recorded by a SpectraMax M5 microplate reader from Molecular Device):

$$\left[{\text{Ca}}^{2+}\right]\text{i}=K_{\text{d}}\text{Q}\frac{\left(R-R_{\text{min}}\right)}{\left(R_{\text{max}}-R\right)}\text{,}$$

where *R* represents the ratio of 510-nm emission intensity, i.e., F_λ1_/F_λ2_, of Fura-2, and the values of *R*_min_, *R*_max_ Q and *K*_d_ (for Fura-2) were obtained by a *in situ* calibration assay (Petr and Wurster, 1997).

This assay was also used for correcting background fluorescence of the Fura-2 indicator. Briefly, a series of solutions containing different concentrations of calcium ions were first prepared by diluting the CaEGTA stock solution at 37°C, pH 7.2, mimicking the *in vivo* situation. The solution, along with 10 μM ionomycin, was then added to the Fura-2-loaded MEFs. Following the measurement of the F_λ1_ and F_λ2_ of Fura-2, cells were incubated for an additional 20 min with 6 mM MnCl_2_ to quench the fluorescence of the indicator. The remaining fluorescence was considered as background fluorescence, which was subtracted from the total fluorescence, afterwards. The difference between the total fluorescence and the background fluorescence was considered as the actual cytosolic fluorescence. The corrected values of F_λ1_ were increased proportionally to the increase of Ca^2+^ concentrations in CaEGTA solutions, while the corrected values of F_λ2_ were changed independent of Ca^2+^ concentrations. The value of *R*_min_, was generated from the corrected F_λ1_/F_λ2_ ratio at zero free Ca^2+^, and *R*_max_ from 27.96 μM (saturating) Ca^2+^. Q value was determined by the ratio of F_λ2_ at zero free Ca^2+^, to F_λ2_ at 27.96 μM free Ca^2+^. A series values of log $\left[\left(R-R_{\min}\right)/\left(R_{\max}-R\right)\times \text{Q}\right]$, generated from each solution, were then plotted with the log value of its free Ca^2+^ concentration. After that, a straight line was yielded, and a linear regression equation: y = 1.2956x + 8.7237 (R^2^ = 0.935), was generated. The log of *K*_d_ was determined according to the x-intercept of this straight line. We determined that the *K*_d_ for Fura-2 is 184.7872 nM.

#### Purification of Lysosomes

Lysosomes were purified by Lysosome Isolation Kit according to the manufacturer’s instructions, with minor modifications. Briefly, MEFs from sixty 10-cm dishes (60-80% confluence) were collected by directly scrapping at room temperature, followed by centrifugation for 5 min at 500 *g* at 37°C. Cells were resuspend in 7 mL of 1× Extraction Buffer containing protease inhibitor cocktail at room temperature, and were dounced in a 7-mL Dounce homogenizer (Sigma, cat. P0610) for 120 strokes on ice followed by centrifuging for 10 min at 1,000 *g*, 4°C, yielding post-nuclear supernatants (PNS). The PNS were then centrifuged for 20 min at 20,000 *g* and the pellet was suspended by 1× Extraction Buffer by gentle pipetting, generating Crude Lysosomal Fraction (CLF). The volume of CLF was adjusted to 2.4 mL and then equally divided into six 1.5 mL Eppendorf tubes (400 μl per tube). 253 μL of OptiPrep and 137 μL of 1× OptiPrep Dilution Buffer were added to each CLF, and mixed by gentle pipetting. The mixture is defined as the Diluted OptiPrep Fraction (DOF). Each DOF (0.8 mL) was loaded to an 11 × 60 mm centrifuge tube at the top of 27% (0.4 mL) and 22.5% (0.5 mL) OptiPrep solution cushions, and then overlaid with 16% (1 mL), 12% (0.9 mL) and 8% (0.3 mL) OptiPrep solutions. The tube was then centrifuged on a SW60 Ti rotor (Beckman) at 150,000 *g* for 4 hr at 4°C, and the fraction at the top of 12% OptiPrep solution was collected as the crude lysosome fraction. The fraction was diluted with two volumes of PBS, followed by centrifugation at 20,000 *g* for 20 min. The supernatant was then aspirated, and the sediment was the lysosome fraction.

#### Measurement of v-ATPase Activity *In Vitro*

For each assay, lysosomes purified from two 10-cm dishes of MEFs were used. ATP hydrolysis activity was measured using a coupled spectrophotometric method as described previously (Shao and Forgac, 2004) with some modifications. Briefly, lysosomes were suspended in ATPase assay buffer (50 mM NaCl, 30 mM KCl, 20 mM HEPES-NaOH, pH 7.0, 10% glycerol, 1 mM MgCl_2_, 1.5 mM phosphoenolpyruvate, 0.35 mM NADH, 20 U/mL pyruvate kinase, and 10 U/mL lactate dehydrogenase) with 5 μM ConA (for calculating the v-ATPase-specific ATP hydrolysis activity) or DMSO, and warmed at 37°C for 10 min. The assay was initiated by the addition of 5 mM ATP, and the OD_341_ was continuously recorded by a SpectraMax M5 microplate reader.

ATP-dependent proton transport activity was measured by the initial rate of ATP-dependent fluorescent quenching of FITC-dextran, as described previously (Liberman et al., 2014, Trombetta et al., 2003). Briefly, lysosomes were loaded with FITC-dextran by incubating cells in DMEM supplemented with 2 mg/mL FITC-dextran (final concentration) on ice for 5 min, then transferred to a 37°C incubator for 30 min. Cells were washed with DMEM for three times and incubated with DMEM for another 30 min at 37°C to allow transport of FITC-dextran to lysosomes. Cells were collected and lysosomes were purified as described above. The lysosomes were resuspended in assay buffer (125 mM KCl, 1 mM EDTA, 20 mM HEPES, pH 7.5, with KOH) and were balanced on ice for 1 hr, then mixed with 5 μM ConA (for calculating the v-ATPase-specific proton transport activity) or DMSO, then warmed at 37°C for 10 min. Fluorescence of FITC was recorded with excitation at 490 nm and emission at 520 nm using a SpectraMax M5 microplate reader. The initial slope of fluorescence quenching was measured after addition of 5 mM Mg-ATP (final concentration).

#### Isolation of Light Organelles

Light organelles were isolated as described previously (Steinberg et al., 2010, Zoncu et al., 2011). Briefly, cells were scraped and spun down at 200 g at room temperature, and then resuspended in 750 μL per 15-cm dish of fractionation buffer (140 mM KCl, 1 mM EGTA, 5 mM MgCl_2_, 50 mM sucrose, 20 mM HEPES, pH 7.4, supplemented with 2.5 mM ATP, amino acids and protease inhibitor cocktail) at room temperature, and were mechanically broken by spraying 6 times through a 22G needle, yielding PNS after spinning at 2,000 *g* for 5 min. The PNS was then spun at max speed for 15 min in a tabletop centrifuge. The pellets are light organelles and supernatants are the cytosol.

#### *In Vitro* Reconstitution for Lysosomal Binding Assays

Light organelles then re-suspended with 100 μL fractionation buffer containing 10 μM FBP were incubated at 37°C in a thermomixer at 400 rpm for 15 min. Some 500 μL of cytosol was then added to the mixture, followed by incubation at 37°C for another 25 min. The mixtures were lysed with 800 μL ODG buffer, followed by IP with antibody against LAMTOR1.

#### Purification of ER

Method for isolating pure ER was optimized by combining the traditional microsome-based density gradient isolation method (Endoplasmic Reticulum Isolation Kit developed by Sigma-Aldrich) with the cell surface biotinylation reaction method (developed and optimized by Pierce). Briefly, MEFs from 40 10-cm dishes (80% confluence) were quickly washed with ice-cold PBS (10 mL each dish) twice, followed by incubating with 250 μg/mL of sulfo-NHS-SS-biotin (freshly dissolved in ice-cold PBS, 10 mL each dish) for 30 min with gentle agitate on an orbital shaker at 4°C. Some 500 μL of 1 M Tris (pH 8.0 at 4°C) was then added to each dish to quench the biotinylation reaction. Cells were collected afterwards by directly scrapping, followed by centrifugation at 600 *g* for 5 min, and then washed with 40 mL of ice-cold PBS twice. Cells were then re-suspended in 10 mL of 1× Hypotonic Extraction Buffer and then incubated at 4°C for 30 min, with gentle mixing in the middle. Cells were then centrifuged at 600 *g* at 4°C for 5 min, and the pellet was re-suspended with 6 mL of 1× Isotonic Extraction Buffer, followed by mixing in a 7-mL Dounce homogenizer for 10 strokes. The homogenate was centrifuged at 1,000 *g* for 10 min at 4°C, and the supernatant (PNS) was further centrifuged at 12,000 *g* for 15 min at 4°C, yielding the supernatant as the post-mitochondrial fraction (PMF). The PMF was loaded in two 11 × 60 mm centrifuge tubes and then centrifuged on an SW60 Ti rotor (Beckman) at 100,000 *g* for 1 hr at 4°C. The pellet was re-suspended with 0.5 mL of 1× Isotonic Extraction Buffer, and was mixed in a 2-mL Dounce homogenizer for 20 strokes, yielding the microsomal suspension. The suspension was mixed with 0.25 mL of OptiPrep, and was carefully layered on the top of 1 mL of 30% OptiPrep solution (by mixing 0.5 mL of OptiPrep with 0.5 mL of 1× Isotonic Extraction Buffer) in an 11 × 60 mm centrifuge tube. Some 2 mL of 15% OptiPrep solution (by mixing 0.5 mL of OptiPrep with 1.5 mL of 1× Isotonic Extraction Buffer) was then carefully layered on the top of the sample. The tube was then centrifuged on an SW60 Ti rotor at 150,000 *g* for 3 hr at 4°C. The top 0.6 mL of 15% OptiPrep solution was discarded, and the following 200 μL of fraction was collected as the crude ER fraction. The fraction was then incubated with 100 μL of NeutrAvidin Agarose (pre-balanced by 1× Isotonic Extraction Buffer) for another 2 hr. The supernatant was then mixed with 50 μL of 5× SDS sample buffer for immunoblotting analysis.

#### Protein Production

Full length cDNAs encoding calmodulin and CaMKK2 were cloned into pET-28a (Novagen) and pGEX4T-1 (GE Healthcare) vectors, respectively, and transformed into the *E. coli* strain BL21 (DE3). The transformed cells were induced with 0.1 mM IPTG at an optical density of 1.0 at 600 nm. After incubating for another 4 hr at 30°C, the cells were collected. For His-tagged calmodulin, cells were homogenized in a His binding buffer (50 mM sodium phosphate, pH 7.4, 150 mM NaCl, 1% Triton X-100, 5% glycerol, and 10 mM imidazole), and for GST-tagged CaMKK2, with a GST binding buffer (PBS supplemented with 10 mM β-mercaptoethanol and 1% Triton X-100). The homogenates were then sonicated, and were subjected to ultracentrifugation at 150,000 *g* for 30 min, followed by purification of calmodulin with Nickel Affinity Gel or CaMKK2 with Glutathione Sepharose 4 Fast Flow Gel. The Nickel Affinity Gel was then washed with 100 times the volume of His wash buffer (50 mM sodium phosphate, pH 7.4, 150 mM NaCl, and 20 mM imidazole), and the Glutathione Sepharose gel with 100 times the volume of PBS. Calmodulin was eluted from the resin by His elution buffer (50 mM sodium phosphate, pH 7.4, 150 mM NaCl, and 250 mM imidazole), and CaMKK2 was eluted by GST elution buffer (50 mM Tris–HCl, pH 8.0, and 10 mM reduced glutathione). Proteins were concentrated to approximately 3 mg/mL by ultrafiltration (Millipore, UFC905096 for CaMKK2, and UFC901096 for calmodulin), then subjected to a gel filtration column (GE Healthcare, Superdex-200) balanced with an AMPK kinase assay buffer containing 50 mM MOPS, pH 7.0, 100 mM NaCl, 0.1 mM EDTA, and 10 mM MgCl_2_. Heterotrimeric AMPK was expressed in the *E. coli* strain BL21 (DE3) as described previously (Neumann et al., 2003) and purified through a gel filtration column as described above.

#### Kinetic Analysis of CaMKK2 Activity

The enzymatic activity of CaMKK2 was expressed by the activity of AMPK pre-phosphorylated by CaMKK2. The assay was performed as described previously (Chen et al., 2009) with minor modifications. Briefly, GST-tagged CaMKK2 was incubated with Glutathione Sepharose 4 Fast Flow Gel (5 μg of protein/μL gel) at 4°C for 1 hr, followed by washing in an AMPK kinase assay buffer twice. Some 1 μL of immobilized CaMKK2 was then incubated with 5 μg of AMPK, 10 μg of calmodulin in AMPK kinase assay buffer supplemented with 5 mM ATP and desired concentrations of CaCl_2_ (total volume: 60 μL) on a thermomixer at 30°C for 30 min. CaMKK2 was then removed by centrifugation at 2,000 *g*, 4°C for 30 s, resulting in the supernatant that contained phosphorylated AMPK. Activity of the resultant AMPK was determined by incubating 50 μL of the supernatant with 200 μL of 30°C-pre-warmed AMPK kinase assay buffer supplemented with 0.2 mM NADH, 1.0 mM phosphoenolpyruvate, 20 U/mL lactate dehydrogenase, 15 U/mL pyruvate kinase, 5 mM ATP and 200 μM ACC tide (HMRSSMSGLHLVRRR, synthesized by GenScript, China) at 30°C. The OD_341_ was continuously recorded by a SpectraMax M5 microplate reader.

#### Analysis of AMP/ATP and ADP/ATP Ratios

To analyze AMP/ATP and ADP/ATP ratios in mammalian cell lines, mouse liver and muscle, CE (capillary electrophoresis)-MS was performed. Sample preparation for CE-MS was carried out as described previously (Zhao et al., 2014, Zhao et al., 2015), with some modifications. In general, each measurement requires cells collected from a 10-cm dish (60-70% confluence) or 20 mg of freeze-clamped mouse liver. Cells/tissues were rinsed with 20 mL of 5% mannitol solution (dissolved in water) and instantly frozen in liquid nitrogen. Cells/tissues were then lyzed with 1 mL of methanol containing internal standards 1 (IS1 used to standardize the metabolite intensity and to adjust the migration time), and cells were scrapped off from the dish. Lysate was then mixed with 1 mL of chloroform and 400 μL of water by 20 s of vortexing. After centrifugation at 15,000 *g* for another 15 min at 4°C, 450 μL of aqueous phase was collected and was filtrated through a 5 kDa cutoff filter (Millipore, cat. UFC3LCCNB-HMT) by centrifuging at 10,000 *g* for 3 hr at 4°C. The filtered aqueous phase was then freeze-dried in a vacuum concentrator (Labconco, CentriVap Benchtop Centrifugal Vacuum Concentrator, equipped with a CentriVap -84°C Cold Trap and a Scroll Vacuum Pump) and then dissolved in water containing internal standards 3 (IS3 to adjust the migration time). 20 μL of re-dissolved solution was then loaded into an injection vial with a conical insert for CE-TOF MS (Agilent Technologies 7100, equipped with 6224 mass spectrometer) analysis. Data were processed using Qualitative Analysis B.06.00. Levels of AMP, ADP, and ATP were measured using full scan mode with m/z 346.0558, 426.0221, and 505.9885, respectively. Note that a portion of ADP and ATP could lose one phosphate group during in-source-fragmentation thus leaving same m/z ratios as AMP and ADP, and should be corrected according to their different retention time in capillary. Therefore, the total amount of ADP is the sum of the latter peak of the m/z 346.0558 spectrogram and the former peak of the m/z 426.0221 spectrogram, and *ditto* for ATP.

#### Measurement of NAD^+^

To measure NAD^+^ levels in muscle, some 50 mg of fleshly excised gastrocnemius muscle was immediately frozen in liquid nitrogen and homogenized in 1 mL of ice-cold methanol. Some 1 mL of chloroform and 400 μL of water were then added to the homogenate sequentially, followed by 20 s of vortexing. After centrifugation at 15,000 *g* for 15 min at 4°C, 350 μL of aqueous phase was collected, lyophilized in a vacuum concentrator, and then dissolved in 500 μL of 20% methanol. The remaining solution was dried at a fume hood at room temperature for three days to obtain the dry weight of protein (for calculating the relative concentrations of NAD^+^). Measurement of NAD^+^ level was based on (Katsyuba et al., 2018) using a QTRAP MS (SCIEX, QTRAP 6500 plus) interfaced with a UPLC system (Waters, ACQUITY UPLC system) with minor modifications. In brief, 2 μL of each sample was loaded onto a SeQuant ZIC-pHILIC column (5 μm, 100 × 2.1 mm, Merck). Mobile phase buffer A was 15 mM ammonium acetate in water (pH adjusted to 9.7 with ammonium hydroxide), and mobile phase buffer B 90% acetonitrile. During the analysis, the column was maintained at 40°C and sample at 8°C. The gradients were as follows: t = 0-2 min, 95% B; t = 15-18 min, 45% B; t = 18-22 min, 95% B, with a constant flow rate at 0.2 mL/min. The QTRAP mass spectrometer was run in negative mode with multiple reactions monitoring mode (MRM), and declustering potentials and collision energies were optimized using analytical standards of NAD^+^. The following transition was used for monitoring NAD^+^: 662.0/540.1. The retention time of NAD^+^ was 10.3 min.

#### Determination of Lysosomal Membrane Potential

To measure FRET in MEFs, cells were first loaded with Rhodamine-PE (Rh-PE, FRET acceptor) followed by addition of the membrane-permeant oxonol dye DiBAC_4_(3) (FRET donor) to the culture medium. The emission intensities of the donor, acceptor and FRET channels were separately measured according to the images taken by a Zeiss LSM 780. The bleed-through parameters of donor and acceptor were then calculated according to the values of cFRET as described previously (Koivusalo et al., 2011). Briefly, Rh-PE was freshly prepared by adding 25 μL of 1 mg/mL Rh-PE solution in CHCl_3_/MeOH (1:1) dropwise into 4 mL of 0.5 mg/mL fatty acid-free BSA in PBS accompanied with vigorously vortexing for 1-2 min. MEFs grown in 35-mm glass bottom dishes were washed with ice-cold PBS, and incubated with the Rh-PE-BSA complex for 10 min on ice to allow for the entry of this complex into the cell. Cells were then washed with medium, and incubated at 37°C for another 90 min. A 300 nM DiBAC_4_(3) was added to the medium and the cells were balanced for 5 min at 37°C, then washed and imaged in the live-cell incubation chamber at a regular interval. Different fields of the donor, acceptor and FRET channels were first acquired, and the filter set parameters (listed as excitation/emission filters, all in nanometers ± bandwidth) used for FRET calculation were as follows: donor DiBAC_4_(3) at 475 ± 60/510 ± 40, acceptor Rh-PE at 543 ± 22/580 ± 25, and FRET at 475 ± 60/580 ± 25. For the determination of the donor and acceptor bleed-through coefficients, samples loaded with donor or acceptor only were imaged. Cells loaded with donor alone, showed negligible cFRET signal. Similarly, cFRET was virtually absent when only the acceptor was present. In MEFs that had been loaded with both the donor and the acceptor, the cFRET signal was detectable and recorded, and was calculated using the method as described previously (Koivusalo et al., 2011). All parameters, including exposure times and camera gains, were kept constant across each experiment. Images were background-subtracted before analysis.

### Quantification and Statistical Analysis

1-way or 2-way ANOVA with post hoc analysis was used to compare values among different experimental groups. For experiments with only two groups, a two-tailed Student’s t test was used as specified in the figure legends. For ANOVA, the homogeneity of variance was tested by Levene’s test. If the results are similar, the Tukey's test was preceded, and if not, the Games-Howell’s test was preceded. Similar procedures were followed when Student's t test was performed. No samples or animals were excluded from the analysis. Tests were performed with SPSS Statistics 17.0 program or Prism 6, and p < 0.05 was considered statistically significant. Statistical significance is shown as ^∗^ p < 0.05, ^∗∗^ p < 0.01, ^∗∗∗^ p < 0.001; N.S., not significant.

### Data and Software Availability

All software and data used are freely available either online through various servers (see Key Resources Table). Source data for blot quantification can be found in Mendeley Data: https://data.mendeley.com/datasets/p3zgm58ksw/draft?a=10c923e7-0295-4f9c-ad63-e7e811c789da

## Supplemental Information

Document S1. Figures S1–S7 and Table S1

Document S2. Article plus Supplemental Information
