# Transient Receptor Potential V Channels Are Essential for Glucose Sensing by Aldolase and AMPK

**DOI:** 10.1016/j.cmet.2025.01.003

**Published:** 2025-02-04

**Authors:** Mengqi Li, Chen-Song Zhang, Yue Zong, Jin-Wei Feng, Teng Ma, Meiqin Hu, Zhizhong Lin, Xiaotong Li, Changchuan Xie, Yaying Wu, Dong Jiang, Ying Li, Cixiong Zhang, Xiao Tian, Wen Wang, Yanyan Yang, Jie Chen, Jiwen Cui, Yu-Qing Wu, Xin Chen, Qing-Feng Liu, Jianfeng Wu, Shu-Yong Lin, Zhiyun Ye, Ying Liu, Hai-Long Piao, Li Yu, Zhuan Zhou, Xiao-Song Xie, D. Grahame Hardie, Sheng-Cai Lin

## Main text

(Cell Metabolism *30*, 508–524.e1–e12; September 3, 2019)

In the originally published article, the immunofluorescent staining images in Figures 1E and 2G were incorrect. The error does not affect the presented data, analysis, or conclusion reported in the paper. We deeply regret the mistake and apologize for any inconvenience or confusion it may have caused.

**Figure 1E:**
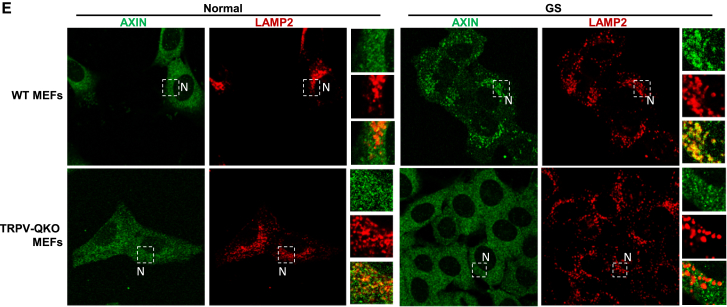
TRPV1–4 Interact with FBP-Unoccupied Aldolase and Are Physically Required for Lysosomal AMPK Activation in Low Glucose

**Figure 2G:**
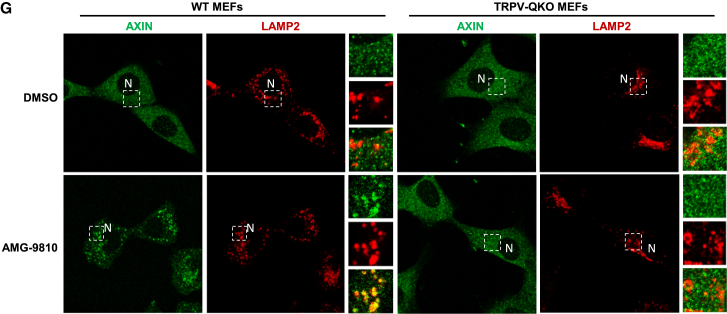
Glucose Starvation Blocks Ca^2+^ Channel Activity of TRPVs

