# Supplementary material for: Transient Receptor Potential V Channels Are Essential for Glucose Sensing by Aldolase and AMPK
Source: Cell Metab. 2019 Sep 3;30(3):508–524.e12. doi: 10.1016/j.cmet.2019.05.018 (PMC6720459; doi:10.1016/j.cmet.2019.05.018)
Supplement: Document S1. Figures S1–S7 and Table S1 [file mmc1.pdf]

Cell Metabolism, Volume *30*

Supplemental Information

Transient Receptor Potential V Channels Are Essential for Glucose Sensing by Aldolase and AMPK

Mengqi Li, Chen-Song Zhang, Yue Zong, Jin-Wei Feng, Teng Ma, Meiqin Hu, Zhizhong Lin, Xiaotong Li, Changchuan Xie, Yaying Wu, Dong Jiang, Ying Li, Cixiong Zhang, Xiao Tian, Wen Wang, Yanyan Yang, Jie Chen, Jiwen Cui, Yu-Qing Wu, Xin Chen, Qing-Feng Liu, Jianfeng Wu, Shu-Yong Lin, Zhiyun Ye, Ying Liu, Hai-Long Piao, Li Yu, Zhuan Zhou, Xiao-Song Xie, D. Grahame Hardie, and Sheng-Cai Lin

Figure S1

A

Gene Name	Score	Peptide	
Tub	2861.18	2164	Cytoskeleton
Act	1148.55	990	
Myh9	904.16	630	
Fln	503.34	259	
Gapdh	237.73	162	Metabolic enzymes
Phgdh	151.49	86	
Pfkm, Pfkp	11.13	7	
Tpi1	10.52	6	
Atp6v1a	37.04	19	v-ATPase subunits
Atp6v1e1	7.78	5	
Atp6v0d1	5.33	3	
Atp6v1b2	4.74	3	
Irgm1	160.29	81	Novel candidates
Mta1	27.04	19	
Nlrx1	4.53	3	
Trpv4	4.01	2	

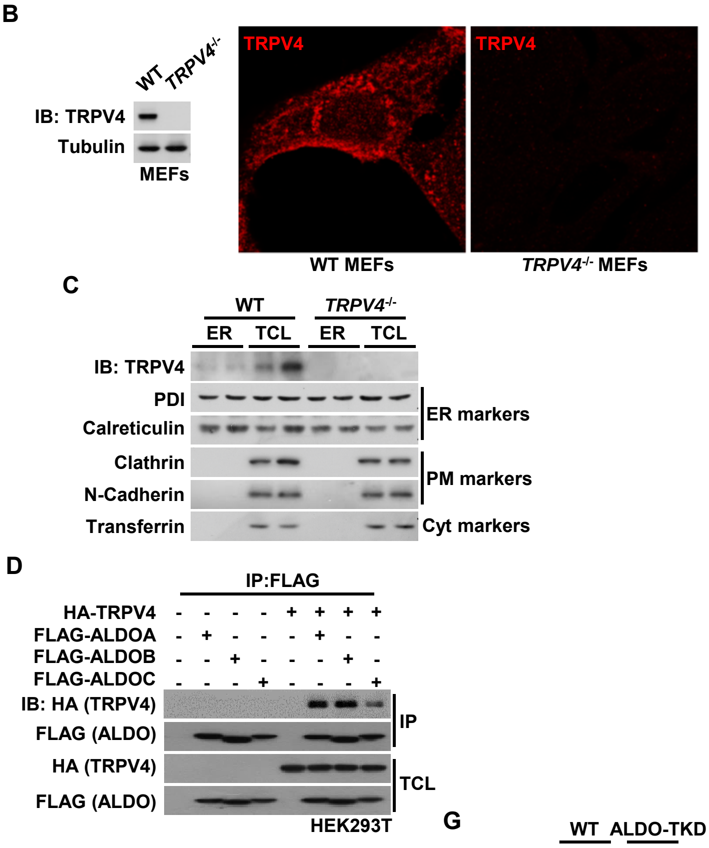

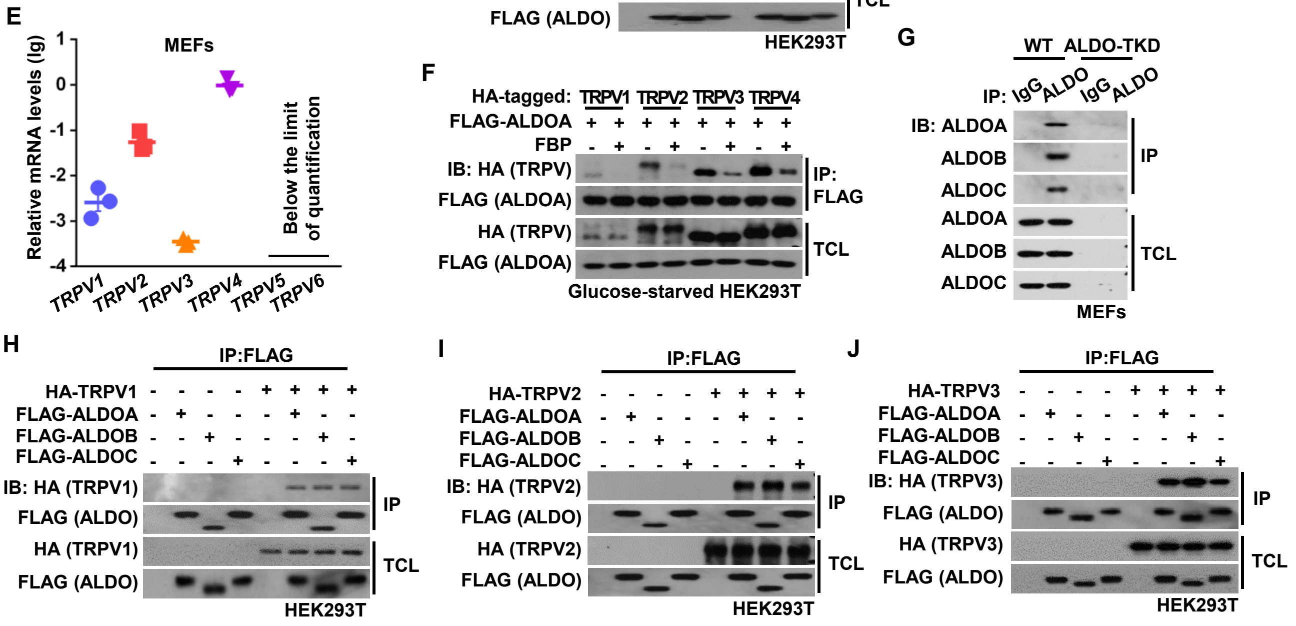

Figure S1. TRPVs are Novel Interactors of Aldolase. Related to Figure 1

(A) Summary of mass spectrometry results using HA-tagged ALDOA as bait.

(B) Validation of TRPV4 antibody used for immunoblotting and immunofluorescent staining. In left panel, WT and *TRPV4*$^{-/-}$ MEFs were lyzed and followed by immunoblotting with antibodies indicated. In right panel, the goat anti-TRPV4 antibody was used, followed by Alexa Fluor 568 donkey anti-goat IgG. Note that when TRPV4 was knocked out, i.e., in *TRPV4*$^{-/-}$ MEFs, no specific band (left panel) or staining signal (right panel) was detected.

(C) A portion of TRPV is localized on ER. The ER fractions (purified as described in STAR Methods), along with total cell lysates of MEFs were subjected to immunoblotting using the indicated antibodies.

(D, H, I, and J) Interactions between ectopically expressed TRPV1-4 and ALDOA-C. HEK293T cells were transfected with FLAG-tagged ALDOA, ALDOB and ALDOC, together with HA-tagged TRPV4 (D), TRPV1 (H), TRPV2 (I) or TRPV3 (J). Immunoprecipitation was performed using ANTI-FLAG® M2 Affinity Gel, eluted with FLAG® Peptide, and followed by immunoblotting with antibodies indicated.

(E) TRPV1-4, but not TRPV5 and TRPV6, are expressed in MEFs. MEFs (3 dishes per group) were lyzed in TRIzol reagent and total RNA was extracted. The mRNA levels of TRPV1-6 were then analyzed by real-time PCR.

(F) FBP dampens the association between TRPV and aldolase. HEK293T cells were transfected with FLAG-tagged ALDOA and HA-tagged TRPV1-4. The transfected cells were starved in glucose-free DMEM for 2 hr, and lyzed, followed by addition of 200 μM FBP. IP was performed using ANTI-FLAG® M2 Affinity Gel, eluted with FLAG® Peptide, and the immunoprecipitates were analyzed by immunoblotting with antibodies indicated.

(G) Validation of the rabbit polyclonal antibody raised for immunoprecipitating endogenous aldolase. Bacterially expressed and purified full-length mouse ALDOA was used for immunization in rabbits. MEFs were lyzed, and the endogenous aldolase was immunoprecipitated with the raised polyclonal antibody, using IgG as a control, followed by immunoblotting with the antibodies indicated. The result shows that this antibody is able to react with all three isozymes of aldolase.

Experiments in (C) and (E) were performed twice and others three times.

Figure S2

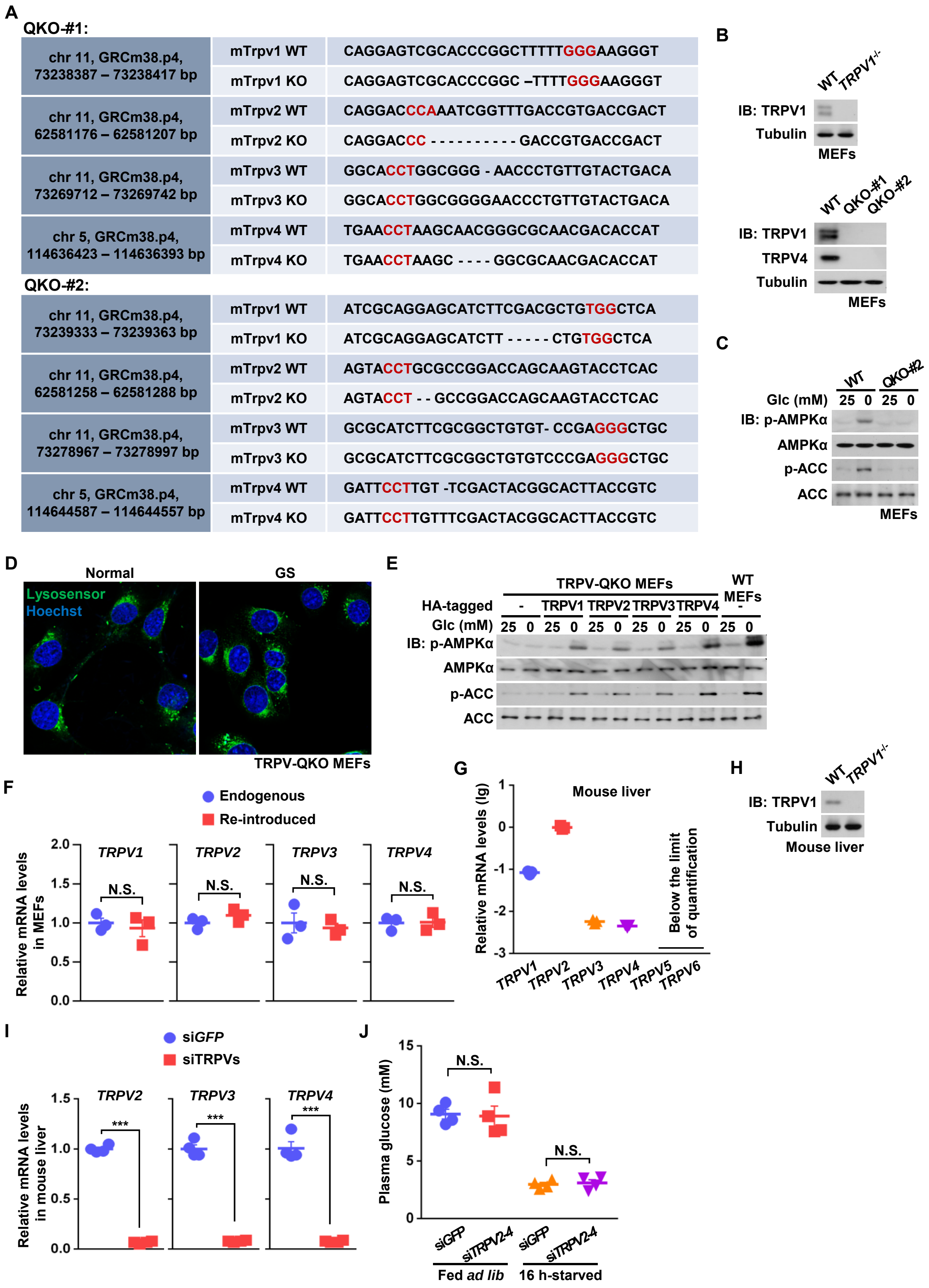
A
QKO-#1:
chr 11, GRCm38.p4, 73238387 – 73238417 bp
mTrpv1 WT CAGGAGTCGCACCCGGCTTTTTGGGAAGGGT
mTrpv1 KO CAGGAGTCGCACCCGGC –TTTTGGGAAGGGT
chr 11, GRCm38.p4, 62581176 – 62581207 bp
mTrpv2 WT CAGGACCCAAATCGGTTTGACCGTGACCGACT
mTrpv2 KO CAGGACCC - - - - - - - - - - - GACCGTGACCGACT
chr 11, GRCm38.p4, 73269712 – 73269742 bp
mTrpv3 WT GGCACCTGGCGGG - AACCCTGTTGTACTGACA
mTrpv3 KO GGCACCTGGCGGGGAACCCTGTTGTACTGACA
chr 5, GRCm38.p4, 114636423 – 114636393 bp
mTrpv4 WT TGAACCTAAGCAACGGGCGCAACGACACCAT
mTrpv4 KO TGAACCTAAGC - - - - GGCGCAACGACACCAT
QKO-#2:
chr 11, GRCm38.p4, 73239333 – 73239363 bp
mTrpv1 WT ATCGCAGGAGCATCTTCGACGCTGTGGCTCA
mTrpv1 KO ATCGCAGGAGCATCTT - - - - - CTGTGGCTCA
chr 11, GRCm38.p4, 62581258 – 62581288 bp
mTrpv2 WT AGTACCTGCGCCGGACCAGCAAGTACCTCAC
mTrpv2 KO AGTACCT - - GCCGGACCAGCAAGTACCTCAC
chr 11, GRCm38.p4, 73278967 – 73278997 bp
mTrpv3 WT GCGCATCTTCGCGGCTGTGT- CCGAGGGCTGC
mTrpv3 KO GCGCATCTTCGCGGCTGTGTCCCGAGGGCTGC
chr 5, GRCm38.p4, 114644587 – 114644557 bp
mTrpv4 WT GATTCCTTGT -TCGACTACGGCACTTACCGTC
mTrpv4 KO GATTCCTTGTTTCGACTACGGCACTTACCGTC
B
WT TRPV1-/-
IB: TRPV1
Tubulin
MEFs
WT QKO-#1 QKO-#2
IB: TRPV1
TRPV4
Tubulin
MEFs
C
WT QKO#2
Glc (mM) 25 0 25 0
IB: p-AMPKα
AMPKα
p-ACC
ACC
MEFs
D
Normal
GS
Lysosensor
Hoechst
TRPV-QKO MEFs
E
TRPV-QKO MEFs
WT MEFs
HA-tagged - TRPV1 TRPV2 TRPV3 TRPV4 -
Glc (mM) 25 0 25 0 25 0 25 0 25 0 25 0
IB: p-AMPKα
AMPKα
p-ACC
ACC
F
Endogenous
Re-introduced
Relative mRNA levels in MEFs
TRPV1
TRPV2
TRPV3
TRPV4
N.S.
G
Mouse liver
Relative mRNA levels (lg)
Below the limit of quantification
TRPV1 TRPV2 TRPV3 TRPV4 TRPV5 TRPV6
H
WT TRPV1-/-
IB: TRPV1
Tubulin
Mouse liver
I
siGFP
siTRPVs
Relative mRNA levels in mouse liver
TRPV2
TRPV3
TRPV4
***
J
Plasma glucose (mM)
N.S.
siGFP siTRPV2-4 siGFP siTRPV2-4
Fed ad lib
16 h-starved

Figure S2. TRPVs are required for lysosomal AMPK activation in low glucose. Related to Figure 1

(A) Strategies to generate MEFs with knockout of TRPV1-4 alleles. Two distinct sets of sgRNAs, whose sequences are listed in "STAR Methods", were applied to generate TRPV-QKO MEFs. Two clones (#1 and #2) were established.

(B) Immunoblotting analysis of TRPV1 and TRPV4 protein levels in *TRPV1*$^{-/-}$ (upper panel) and TRPV-QKO (lower panel) MEFs.

(C) Knockout of TRPV blocks AMPK activation under glucose starvation. Experiment was performed as in Figure 1B (rightmost two lanes), except that clone #2 of TRPV-QKO MEFs was used.

(D) Representative images of the experiment shown in Figure 1D. The acidity of lysosomes was determined by the relative fluorescent intensities of LysoSensor (normalized to the intensity of Hoechst).

(E) Re-introduction of a single TRPV member into TRPV-QKO MEFs sufficiently recovers glucose starvation-induced AMPK activation. TRPV-QKO MEFs were infected with lentivirus expressing HA-tagged TRPV1, TRPV2, TRPV3 or TRPV4 (all expressed at close-to-endogenous levels driven by pBOBI vector, as validated in (F). Cells were treated as in Figure 1B, followed by analysis of p-AMPKα and p-ACC.

(F) The expression levels of HA-tagged (re-introduced) TRPVs in TRPV-QKO MEFs were close to their endogenous levels. TRPV-QKO MEFs with HA-tagged TRPV1-4 stably expressed and WT MEFs as a control (3 dishes per group) were lyzed in TRIzol reagent and total RNA was extracted. The mRNA levels of each TRPV were then analyzed by real-time PCR. Results are mean ± SEM. Significance was determined by Student's t-test.

(G) TRPV1-4, but not TRPV5 and TRPV6, are expressed in mouse liver. Some 10 mg of mouse liver (3 mice per group) was lyzed in TRIzol reagent and total RNA was extracted. The mRNA levels of *TRPV1-6* were then analyzed by real-time PCR.

(H) Validation of TRPV1 expression in *TRPV1*$^{-/-}$ mouse liver. Tissues were lyzed, and the protein levels of TRPV1 were determined by immunoblotting.

(I) Validation of knockdown efficiency of TRPV2-4 in mouse liver. Some 10 mg of mouse liver (3 mice per group) was lyzed in 1 mL of TRIzol reagent and total RNA was extracted. The mRNA levels of *TRPV2-4* were then analyzed by real-time PCR. Results are mean ± SEM. Significance was determined by Student′s t-test.

(J) TRPV-deficient mice show comparable plasma glucose with littermate control. Blood glucose was measured after 16-hr starvation (4 mice per group). Results are mean ± SEM. Significance was determined by ANOVA.

Experiments in (G), (I), and (J) were performed twice and others three times.

Figure S3

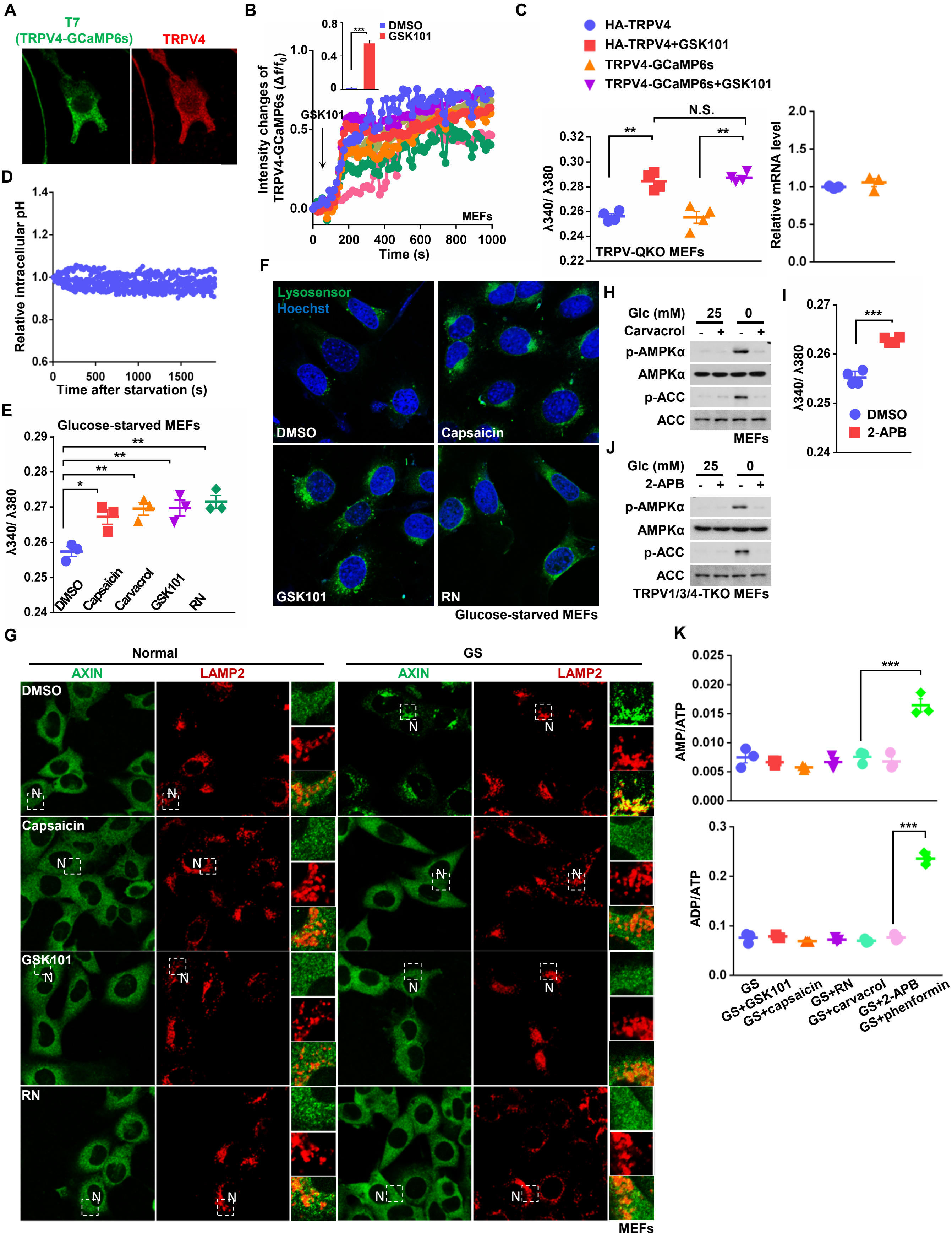
A
T7 (TRPV4-GCaMP6s)
TRPV4
B
DMSO
GSK101
GSK101
Intensity changes of TRPV4-GCaMP6s (Δf/f0)
MEFs
Time (s)
C
HA-TRPV4
HA-TRPV4+GSK101
TRPV4-GCaMP6s
TRPV4-GCaMP6s+GSK101
N.S.
λ340/ λ380
TRPV-QKO MEFs
Relative mRNA level
D
Relative intracellular pH
Time after starvation (s)
E
Glucose-starved MEFs
λ340/ λ380
DMSO
Capsaicin
Carvacrol
GSK101
RN
F
Lysosensor
Hoechst
DMSO
Capsaicin
GSK101
RN
Glucose-starved MEFs
H
Glc (mM)
Carvacrol
p-AMPKα
AMPKα
p-ACC
ACC
MEFs
I
λ340/ λ380
DMSO
2-APB
J
Glc (mM)
2-APB
p-AMPKα
AMPKα
p-ACC
ACC
TRPV1/3/4-TKO MEFs
G
Normal
GS
AXIN
LAMP2
DMSO
Capsaicin
GSK101
RN
MEFs
K
AMP/ATP
ADP/ATP
GS
GS+GSK101
GS+capsaicin
GS+RN
GS+carvacrol
GS+2-APB
GS+phenformin

Figure S3. Knockout of TRPV1-4 Blocks the Lysosomal AMPK Pathway. Related to Figure 2

(A) Validation of cellular localization of TRPV4-GCaMP6s. MEFs were infected with lentivirus carrying TRPV4-GCaMP6s Ca^{2+} indicator. The localization of the indicator was determined by immunofluorescent staining in MEFs stained with rabbit antibody against T7 tag (green, for TRPV4-GCaMP6s), and goat antibody against endogenous TRPV4 (red). Cells were imaged by confocal microscopy after incubating with Alexa Fluor 488 donkey anti-rabbit IgG and Alexa Fluor 594 donkey anti-goat IgG.

(B) Verification of the ability of GSK101 to induce release of calcium. Regularly cultured MEFs were balanced in the live-cell incubation chamber. After 100s of incubation, 50 nM GSK101 was added. The relative fluorescent intensities of GCaMP6s were monitored and plotted as in Figure 2A. Data shown are selected traces from the 7 cells from 2 dishes/experiments. Statistical analysis results were shown as mean ± SEM; p value by Student's t-test.

(C) Verification that the TRPV4-GCaMP6s indicator retains an intact channel activity. MEFs stably expressing HA-TRPV4 and TRPV4-GCaMP6s (at a comparable level, as validated on the right panel, results are mean ± SEM. n = 3) were pre-loaded with Fura-2-AM and treated with 50 nM GSK101 for 15 min. The fluorescent intensity of Fura-2 (ratios of the emission intensities of this dye excited at 340 over those at 380 nm, as described in STAR Methods) was shown on the left panel. Results are mean ± SEM. n = 4. Significance was determined by ANOVA.

(D) Glucose starvation does not alter the global cytosolic pH in MEFs. Cells pre-loaded with SNARF™-5F MEFs were treated with glucose-free DMEM. The ratios of emission intensities measured at 580 ± 10 nm and 640 ± 25 nm were recorded at a regular interval after 2 min incubation with the fresh medium at 37 °C in the live-cell incubation chamber. Data shown are selected traces of 8 cells from 3 dishes/experiments.

(E) Agonists for individual TRPVs elicit fluorescent signals of the Fura-2 calcium indicator. MEFs pre-loaded with Fura-2-AM (see "STAR Methods" for details of loading) were incubated in glucose-free DMEM for 2 hr, followed by addition of 100 nM capsaicin, 50 nM GSK101, 100 μM carvacrol or 0.7 μM RN-1747 (RN) for another 15 min. The fluorescent intensities of Fura-2 were then recorded and analyzed as in (C). Results are mean ± SEM; n = 3, p value by ANOVA.

(F) Representative images of the experiment shown in Figure 2C.

(G) Addition of TRPV agonists impaired the lysosomal translocation of AXIN. MEFs were regularly cultured or glucose-starved for 2 hr, and the localization of AXIN was determined by immunofluorescent staining as described in Figure 1E.

(H and J) Addition of carvacrol or 2-APB blocks glucose starvation-induced AMPK activation. MEFs were incubated in glucose-free DMEM for 2 hr, followed by addition of 100 μM carvacrol (H) or 200 μM 2-APB (J) for another 15 min. The levels

p-AMPKα and p-ACC were then determined.

(I) 2-APB sufficiently elicits fluorescent signal of Fura-2 in TRPV1/3/4-TKO MEFs in which TRPV2 is the only expressed TRPV channel. The 2-h glucose-starved TRPV1/3/4-TKO MEFs loaded with Fura-2-AM were incubated with 200 μM 2-APB for 15 min. The fluorescent intensity of Fura-2 was recorded and analyzed as in (C). Results are mean ± SEM. Significance was determined by Student's t-test.

(K) TRPV agonists did not alter the intracellular adenylate ratios. 2-hr glucose-starved MEFs were treated with TRPV agonists for 15 min or phenformin as a positive control for 3 hr, and adenine nucleotide ratios were measured by CE-MS. Results are mean ± SD; p value by ANOVA, n = 3.

Experiments in this figure were performed three times except for (K) twice.

Figure S4

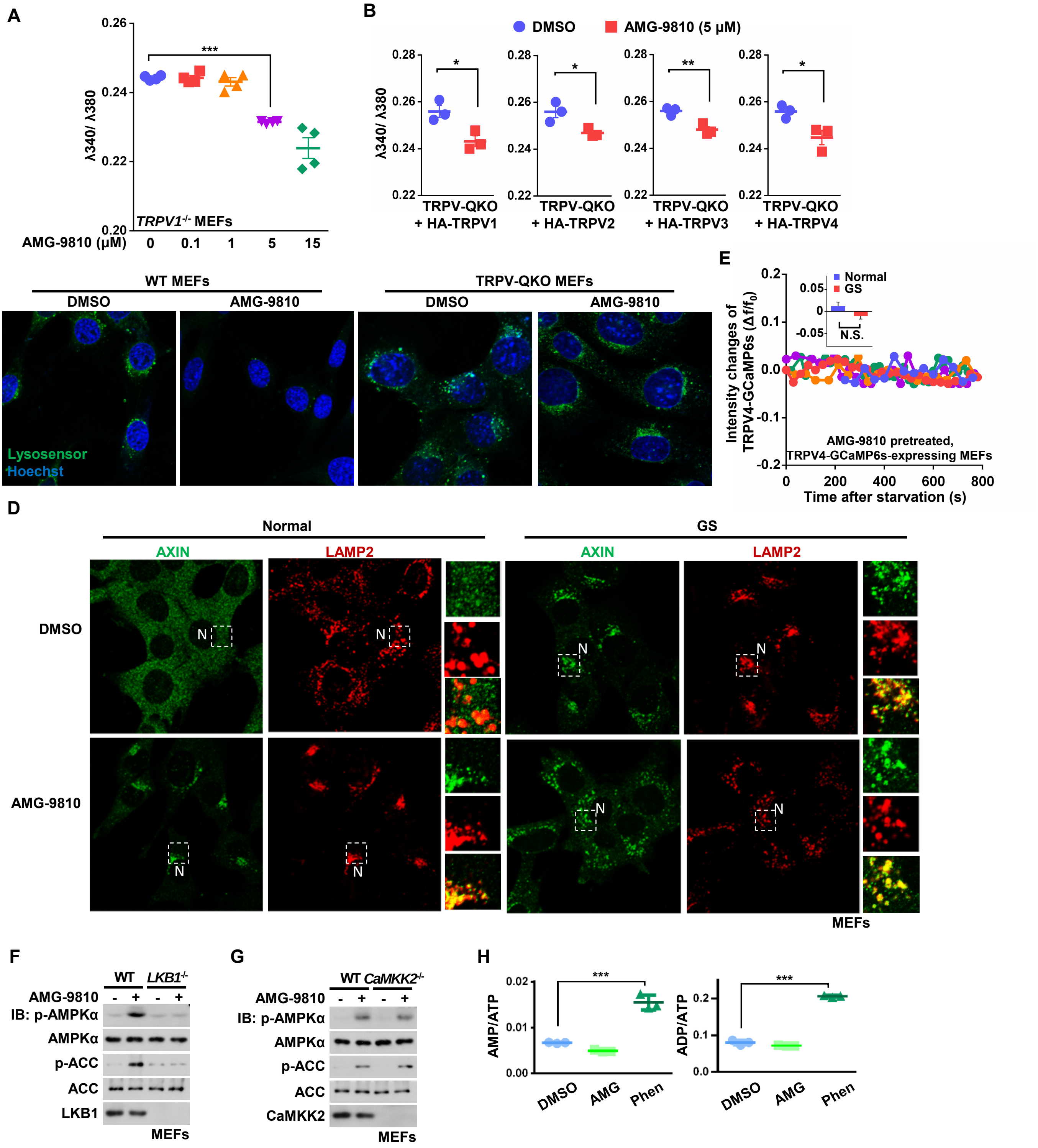
A
λ340/ λ380
***
TRPV1-/- MEFs
AMG-9810 (μM) 0 0.1 1 5 15
B
DMSO
AMG-9810 (5 μM)
TRPV-QKO + HA-TRPV1
TRPV-QKO + HA-TRPV2
TRPV-QKO + HA-TRPV3
TRPV-QKO + HA-TRPV4
C
WT MEFs
TRPV-QKO MEFs
DMSO
AMG-9810
Lysosensor
Hoechst
E
Normal
GS
N.S.
Intensity changes of TRPV4-GCaMP6s (Δf/f0)
AMG-9810 pretreated, TRPV4-GCaMP6s-expressing MEFs
Time after starvation (s)
D
Normal
GS
AXIN
LAMP2
DMSO
AMG-9810
MEFs
F
WT LKB1-/-
AMG-9810
IB: p-AMPKα
AMPKα
p-ACC
ACC
LKB1
MEFs
G
WT CaMKK2-/-
AMG-9810
IB: p-AMPKα
AMPKα
p-ACC
ACC
CaMKK2
MEFs
H
AMP/ATP
ADP/ATP
DMSO
AMG
Phen

Figure S4. Inhibition of TRPV by AMG-9810 Leads to AMPK Activation Even in Normal Glucose. Related to Figure 2

(A and B) AMG-9810 efficiently inhibits TRPV1-4 channels expressed in MEFs. *TRPV1*$^{-/-}$ MEFs (A) or TRPV-QKO MEFs infected with lentivirus expressing HA-tagged TRPV1, TRPV2, TRPV3 or TRPV4 (B, validated in Figure S2F) were loaded with Fura-2-AM. Cells were treated with AMG-9810 at indicated concentration for 30 min. The fluorescent intensity of Fura-2 was then recorded and analyzed as in Figure S3C. Results are mean ± SEM, n = 4 (A) or n = 3 (B); p value by ANOVA (A) or Student's t-test (B).

(C) Representative images of the experiment shown in Figure 2E.

(D) AMG-9810 triggers lysosomal translocation of AXIN. Regularly cultured MEFs were treated with 5 μM AMG-9810 for 30 min, and the localization of AXIN was determined by immunofluorescent staining as described in Figure 1E

(E) AMG-9810 blocks the inhibition of TRPV by glucose starvation. TRPV4-GCaMP6s expressing MEFs were pre-treated with 5 μM AMG-9810 for 30 min and the medium was then switched to glucose-free DMEM. The relative fluorescent intensities of GCaMP6s were monitored and plotted as in Figure 2A. Data shown are selected traces of 5 cells from 5 dishes/experiments. Statistical analysis results were graphed as mean ± SEM; p value by Student's t-test.

(F and G) AMG-9810-induced AMPK activation is dependent on LKB1 and LAMTOR1, but not on CaMKK2. *LKB1*$^{-/-}$ (F), *CaMKK2*$^{-/-}$ (G), along with wild-type (WT) MEFs as control, were treated with 5 μM AMG-9810 for 30 min, followed by analysis of p-AMPKα and p-ACC.

(H) AMG-9810 has no effect on AMP:ATP and ADP:ATP ratios in MEFs. Cells were treated with 5 μM AMG-9810 for 30 min or phenformin (Phen) as a positive control for 2 hr, and adenine nucleotide ratios were measured by CE-MS. Results are mean ± SD; p value by ANOVA, n = 3.

Experiments were performed three times.

Figure S5

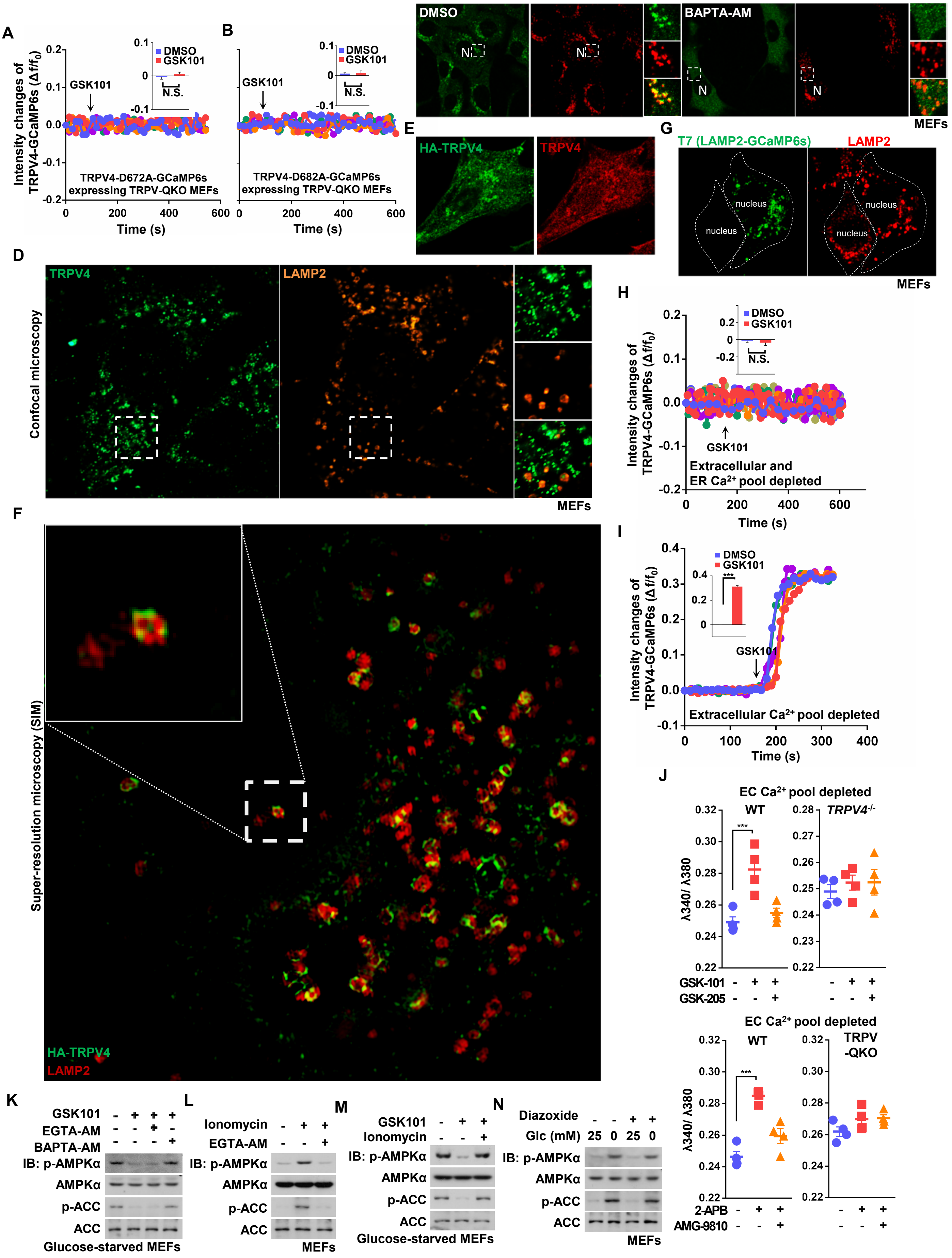

A
Intensity changes of TRPV4-GCaMP6s (Δf/f0)
GSK101
DMSO
GSK101
N.S.
TRPV4-D672A-GCaMP6s expressing TRPV-QKO MEFs
Time (s)
B
GSK101
DMSO
GSK101
N.S.
TRPV4-D682A-GCaMP6s expressing TRPV-QKO MEFs
Time (s)
C
mTOR
LAMP2
DMSO
BAPTA-AM
N
MEFs
E
HA-TRPV4
TRPV4
G
T7 (LAMP2-GCaMP6s)
LAMP2
nucleus
MEFs
D
TRPV4
LAMP2
Confocal microscopy
MEFs
H
Intensity changes of TRPV4-GCaMP6s (Δf/f0)
DMSO
GSK101
N.S.
GSK101
Extracellular and ER Ca2+ pool depleted
Time (s)
F
Super-resolution microscopy (SIM)
HA-TRPV4
LAMP2
I
Intensity changes of TRPV4-GCaMP6s (Δf/f0)
DMSO
GSK101
***
GSK101
Extracellular Ca2+ pool depleted
Time (s)
J
EC Ca2+ pool depleted
WT
TRPV4-/-
λ340/ λ380
GSK-101
GSK-205
EC Ca2+ pool depleted
WT
TRPV -QKO
λ340/ λ380
2-APB
AMG-9810
K
GSK101
EGTA-AM
BAPTA-AM
IB: p-AMPKα
AMPKα
p-ACC
ACC
Glucose-starved MEFs
L
Ionomycin
EGTA-AM
IB: p-AMPKα
AMPKα
p-ACC
ACC
MEFs
M
GSK101
Ionomycin
IB: p-AMPKα
AMPKα
p-ACC
ACC
Glucose-starved MEFs
N
Diazoxide
Glc (mM) 25 0 25 0
IB: p-AMPKα
AMPKα
p-ACC
ACC
MEFs

Figure S5. Depletion of Local Ca^{2+} by BAPTA Triggers Lysosomal AMPK Activation. Related to Figures 3 and 4

(A and B) Validation that the TRPV4 mutants are defective in releasing calcium. MEFs expressing TRPV4-D672A-GCaMP6s (A) and TRPV4-D682A-GCaMP6s (B) were treated as in Figure S3B except that 50 nM GSK101 was added at 100 s. Data shown were selected traces of 5 cells from 5 dishes/experiments (A) and of 6 cells from 6 dishes/experiments (B), respectively. Statistical results were shown as mean ± SEM; p value by Student's t-test.

(C) Effects of BAPTA-AM on lysosomal dissociation of mTOR. MEFs were treated with 100 μM BAPTA-AM for 30 min, and stained with rabbit anti-mTOR antibody (green) and rat anti-LAMP2 antibody (red), respectively, and then imaged by confocal microscopy after incubating cells with Alexa Fluor 488 donkey anti-rabbit IgG and Alexa Fluor 594 donkey anti-rat IgG.

(D) TRPV4 is localized in close vicinity of the lysosome. MEFs were stained with goat anti-TRPV4 antibody (green) and rat anti-LAMP2 antibody (orange), respectively. Images were taken by confocal microscopy with AiryScan detector after incubating cells with Alexa Fluor 488 donkey anti-goat IgG and Alexa Fluor 594 donkey anti-rat IgG.

(E) Validation for the localization of HA-tagged TRPV. *TRPV4*$^{-/-}$ MEFs reintroduced with HA-tagged TRPV4 (expressed at a close-to-endogenous level) was stained using rabbit antibody against HA tag (green). Endogenous TRPV4 was stained using goat antibody against TRPV4 (red). The secondary antibodies Alexa Fluor 488 donkey anti-rabbit IgG and Alexa Fluor 594 donkey anti-goat IgG were used.

(F) Representative SIM images of HA-tagged TRPV4 (green) and LAMP2 (red). The localization of HA-TRPV4 (re-introduced into *TRPV4*$^{-/-}$ MEFs at a close-to-endogenous level) was determined by immunofluorescent staining using rabbit antibody against HA tag, and endogenous LAMP2 was stained using rat antibody against LAMP2. The secondary antibodies Alexa Fluor 488 donkey anti-rabbit IgG and Alexa Fluor 568 goat anti-rat IgG were used.

(G) Validation of the Ca^{2+} indicator LAMP2-GCaMP6s. The localization of LAMP2-GCaMP6s was determined by immunofluorescent staining in MEFs stained with antibody against T7 tag (green, for LAMP2-GCaMP6s), and endogenous LAMP2 (red) using antibody against LAMP2, and were imaged by confocal microscopy after incubating cells with Alexa Fluor 488 donkey anti-rabbit IgG and Alexa Fluor 594 donkey anti-rat IgG.

(H) TRPV4-released Ca^{2+} originates from the ER and extracellular pool. MEFs were incubated in Ca^{2+}-free DMEM containing 5 mM EGTA to remove extracellular Ca^{2+} for 30 min, and with 4 μM thapsigargin for 15 min to deplete the ER Ca^{2+} pool at the same time, then 50 nM GSK101 (at 160 s), followed by determination of the fluorescent signal of the indicator TRPV4-GCaMP6s. The changes of fluorescent intensities were calculated and plotted as schematically represented in Figure 2A.

Data shown are selected traces from the 7 cells from 2 dishes/experiments. Statistical results were shown as mean ± SEM; p value by Student's t test.

(I) TRPV4 can still releases Ca^{2+} when extracellular Ca^{2+} pool is depleted. MEFs were incubated in Ca^{2+}-free DMEM containing 5 mM EGTA to remove extracellular Ca^{2+} for 30 min, then 50 nM GSK101 (at 160 s), followed by determination of the fluorescent signal of the indicator TRPV4-GCaMP6s. The changes of fluorescent intensities were calculated and plotted as schematically represented in Figure 2A. Data shown are selected traces from the 5 cells from 2 dishes/experiments. Statistics were graphed as mean ± SEM; p value by Student's t test.

(J) TRPV1-4 channels expressed in MEFs releases Ca^{2+} from the ER Ca^{2+} pool. *TRPV4*$^{-/-}$ MEFs (upper panel), TRPV-QKO MEFs (lower panel) and wild-type (WT) MEFs as control were incubated in Ca^{2+}-free DMEM containing 5 mM EGTA to remove extracellular Ca^{2+} for 30 min, then 50 nM GSK101 (upper panel) or 200 μM 2-APB (lower panel), followed by determination of the fluorescent intensities of Fura-2 as in Figure S4A. Some 10 μM GSK205 (upper panel) or 5 μM AMG-9810 (lower panel) was added at the beginning of extracellular Ca^{2+} removal as an additional control. Results are mean ± SEM, n = 4; p value by ANOVA.

(K) EGTA-AM that chelates bulk Ca^{2+} has no effect on TRPV-mediated AMPK activation. Glucose-starved MEFs were pre-treated with 100 μM EGTA-AM, or BAPTA-AM as a control, for 30 min, followed by addition of 50 nM GSK101 for another 15 min, and the levels of p-AMPKα and p-ACC were analyzed.

(L) EGTA-AM blocks bulk Ca^{2+}-induced, CaMKK2-dependent, AMPK activation. MEFs were pre-treated 100 μM EGTA-AM for 30 min, followed by addition of 1 μM ionomycin for another 5 min, and the levels of p-AMPKα and p-ACC were analyzed.

(M) Ionomycin exerts an effect on AMPK activation, which is independent of the effects of GSK101. MEFs were starved at glucose-free DMEM for 2 hr, and treated with 50 nM GSK101 and/or 1 μM ionomycin for 15 min, followed by analysis of AMPK activation by immunoblotting.

(N) K_{ATP} channel is not involved in glucose starvation-induced AMPK activation. MEFs were starved in glucose-free DMEM containing 3 μM diazoxide or not for 2 hr, followed by analysis of AMPK activation by immunoblotting.

Experiments in (A), (B), (J), (K), (L), (M), and (N) were performed twice, and the rest three times.

Figure S6

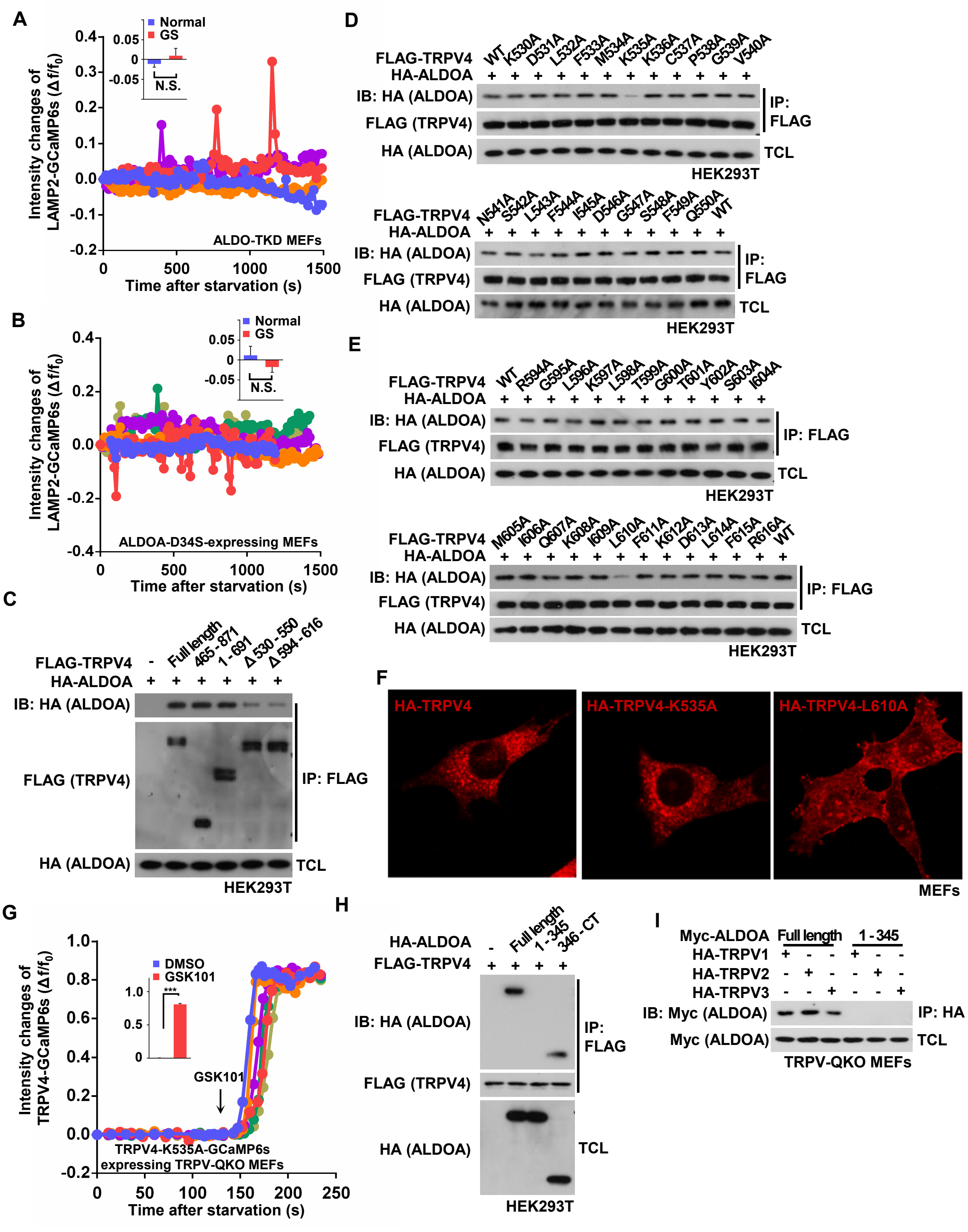

A
B
C
D
E
F
G
H
I
ALDO-TKD MEFs
ALDOA-D34S-expressing MEFs
Time after starvation (s)
Intensity changes of LAMP2-GCaMP6s (Δf/f0)
Normal
GS
N.S.
FLAG-TRPV4
HA-ALDOA
IB: HA (ALDOA)
FLAG (TRPV4)
HA (ALDOA)
IP: FLAG
TCL
HEK293T
Full length
465-871
1-691
Δ530-550
Δ594-616
HA-TRPV4
HA-TRPV4-K535A
HA-TRPV4-L610A
MEFs
Intensity changes of TRPV4-GCaMP6s (Δf/f0)
DMSO
GSK101
TRPV4-K535A-GCaMP6s expressing TRPV-QKO MEFs
1-345
346-CT
Myc-ALDOA
HA-TRPV1
HA-TRPV2
HA-TRPV3
IB: Myc (ALDOA)
Myc (ALDOA)
IP: HA
TRPV-QKO MEFs

Figure S6. TRPV4 is Inhibited upon Aldolase Binding in Low Glucose/FBP. Related to Figure 5

(A) Glucose starvation-induced decrease of Ca^{2+} concentration in the vicinity of lysosomes is not seen in ALDO-TKD MEFs. Experiments were performed as in Figure 4C, except that the ALDO-TKD MEFs expressing LAMP2-GCaMP6s were used. n = 4 cells from 1 dish/experiment. Statistical data were graphed as mean ± SEM; p value by Student's t-test.

(B) Glucose starvation-induced decrease of Ca^{2+} concentration in the vicinity of lysosome is blocked in ALDOA-D34S-expressing MEFs. Experiments were performed as in (A), except that MEFs expressing ALDOA-D34S were used (n = 6 cells from 3 dishes/experiment). Statistical analysis data were graphed as mean ± SEM; p value by Student's t-test.

(C to E) Domain mapping (C) and alanine mutagenesis screening assays (D and E) for determining the site on TRPV4 responsible for aldolase-binding. HA-ALDOA was cotransfected with different FLAG-tagged TRPV4 or its mutants into HEK293T cells. IP was performed using ANTI-FLAG® M2 Affinity Gel, eluted with FLAG® Peptide, and followed by immunoblotting with antibodies indicated.

(F) The L610A mutant of TRPV4 was not properly localized. The intracellular localization of HA-tagged TRPV4, TRPV4-K535A and TRPV4-L610A was determined by immunofluorescent staining in MEFs using rabbit antibody against HA tag, followed with Alexa Fluor 594 donkey anti-rabbit IgG.

(G) GSK101 can still stimulate the TRPV4-K535A mutant, acting on site(s) separable from aldolase. MEFs expressing TRPV4-K535A-GCaMP6s were treated as in Figure S3B except that 50 nM GSK101 was added at 130 s. Data shown are selected traces of 4 cells from 4 dishes/experiments. Statistical analysis data were graphed as mean ± SEM; p value by Student's t test.

(H) Domain mapping for the region of ALDOA responsible for binding to TRPV4. Full length HA-tagged ALDOA or its mutants were cotransfected with FLAG-tagged TRPV4 into HEK293T cells. IP was performed as in (C), and followed by immunoblotting with antibodies indicated.

(I) The ALDOA truncation mutant (aa 1-345) fails to interact with TRPV1-3 as well as TRPV4. TRPV-QKO MEFs re-introduce with HA-tagged TRPV1-3 (used in Figure S2F) were infected with lentivirus expressing full length Myc-tagged ALDOA or ALDOA-1-345. IP was performed using antibodies against HA, and followed by immunoblotting with antibodies indicated.

Experiments in this figure were performed three times except for (C), (H), and (I) twice.

Figure S7

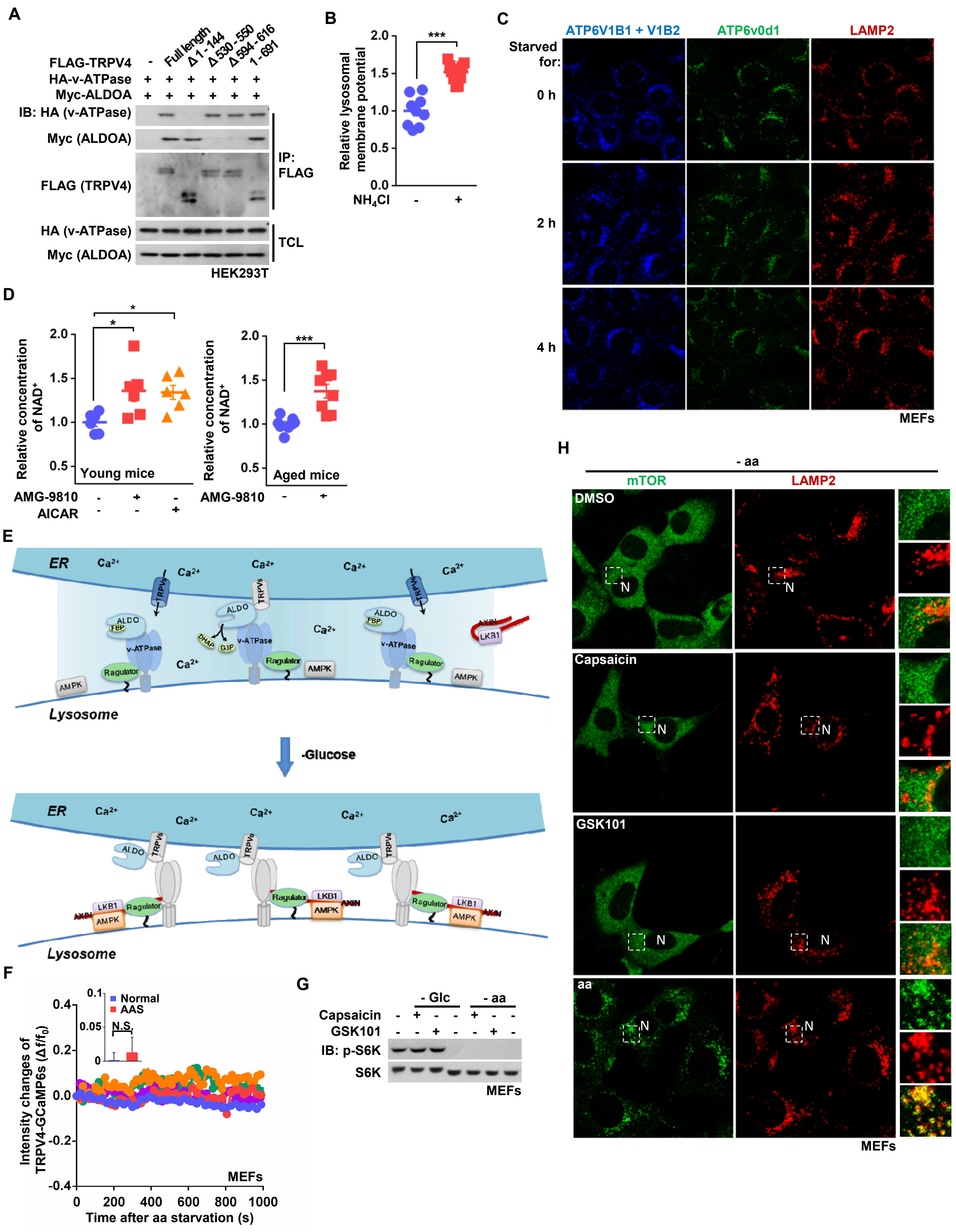
A
FLAG-TRPV4
HA-v-ATPase
Myc-ALDOA
Full length
Δ1-144
Δ530-550
Δ594-616
1-691
IB: HA (v-ATPase)
Myc (ALDOA)
FLAG (TRPV4)
IP: FLAG
HA (v-ATPase)
Myc (ALDOA)
TCL
HEK293T
B
Relative lysosomal membrane potential
NH4Cl
***
C
ATP6V1B1 + V1B2
ATP6v0d1
LAMP2
Starved for:
0 h
2 h
4 h
MEFs
D
Relative concentration of NAD+
Young mice
Aged mice
AMG-9810
AICAR
E
ER
Lysosome
TRPVs
ALDO
FBP
v-ATPase
Ragulator
AMPK
DHAP
G3P
AXIN
LKB1
-Glucose
F
Intensity changes of TRPV4-GCaMP6s (Δf/f0)
Normal
AAS
N.S.
MEFs
Time after aa starvation (s)
G
- Glc
- aa
Capsaicin
GSK101
IB: p-S6K
S6K
MEFs
H
- aa
mTOR
LAMP2
DMSO
Capsaicin
GSK101
aa
N
MEFs

Figure S7. TRPV4 Reconfigures the Association of Aldolase with v-ATPase in the Absence of Ca^{2+}. Related to Figures 6 and 7

(A) Domain mapping for binding site of TRPV4 for v-ATPase. Myc-ALDOA and HA-v-ATPase (V1A as a representative subunit) were cotransfected with FLAG-tagged full-length TRPV4 or its different deletion mutants into HEK293T cells. IP was performed using ANTI-FLAG® M2 Affinity Gel, eluted with FLAG® Peptide, and followed by immunoblotting with antibodies indicated.

(B) Validation of the FRET-based method for measuring lysosomal membrane potential. Experiment was performed as in Figure 6J, except that MEFs were treated with 20 mM NH_4Cl for 5 min, which directly elevates the lysosomal membrane potential (Koivusalo et al., 2011). Results are mean ± SEM; n = 9 (without NH_4Cl added) and n = 11 (with NH_4Cl added) cells, p value by Student's t test.

(C) The integrity of v-ATPase is not affected by glucose starvation (as long as 4 hr). The rabbit anti-ATP6V1B1+V1B2, mouse anti-ATP6v0d1 and rat anti-LAMP2 antibodies were used to co-stain ATP6V1B1+V1B2 (blue), ATP6v0d1 (green), and LAMP2 (red) in MEFs incubated in glucose-free medium for 0 hr, 2 hr, and 4 hr. The secondary antibodies Alexa Fluor 405 goat anti-rabbit IgG, Alexa Fluor 568 donkey anti-mouse IgG, and Alexa Fluor 647 donkey anti-rat IgG were used. The images were acquired by confocal microscopy, and the representative images are shown.

(D) Inhibition of TRPV increases the NAD^+ levels in muscle. Mice at 4 weeks old (left panel) or 1.5-year old (right) were intraperitoneally injected with vehicle (10% (w/v) Kolliphor), 20 mg/kg of AMG-9810 formulated in the vehicle, or 500 mg/kg of AICAR as a control (only in left panel). Twelve hours after injection, muscles were excised and homogenized, followed by analysis of NAD^+ levels by HPLC-MS. Results are mean ± SEM; p value by ANOVA (left panel) or Student's t-test (right panel), n = 6 for groups in (left panel) and n = 14 for groups in (right panel).

(E) Schematic diagrams depicting the analogous roles of the proteins themselves and the released Ca^{2+} of TRPVs (TRPV1-4 characterized in this study) to a capacitor in regulating aldolase:v-ATPase association in sensing glucose/FBP. In abundant glucose, aldolase (ALDO) is occupied with FBP, and at times becomes unoccupied after converting FBP to phosphotrioses hence undergoing oscillation between FBP-bound (away from TRPVs) and unbound (interacting with and inhibiting TRPVs) states. Under this situation, most of TRPV channels are active and maintain the local Ca^{2+} concentration sufficient to prevent TRPVs from interacting with v-ATPase (upper panel). When glucose availability drops, the number of unoccupied aldolase molecules increases, blocking more TRPV channels. The local Ca^{2+} concentration would then drop to a critically low level, allowing TRPV to interact with v-ATPase, which triggers translocation of AXIN:LKB1 to the lysosome and leads to AMPK activation (lower panel).

(F) Amino acid starvation (AAS) does not inhibit the TRPV4 channel. MEFs expressing TRPV4-GCaMP6s were incubated in RPMI-1640 medium containing

amino acids or not. After balancing for 2 min in the live-cell incubation chamber at 37 °C, the images were captured by confocal microscopy at a regular interval (15 s), and the relative fluorescent intensities were analyzed and graphed (n = 5 cells from 3 dishes/experiment). Statistical analysis results were shown as mean ± SEM; p value by Student's t-test.

(G and H) TRPV agonists cannot restore the mTOR's activity and lysosomal localization under amino acid (aa) starvation. MEFs were starved in aa-free RPMI-1640 medium for 30 min, followed by addition of 50 nM GSK101, 100 nM capsaicin, or aa for another 15 min. The activity of mTOR was then analyzes by immunoblotting of phosphorylated S6K-Ser389 levels (G), and the lysosomal localization of mTOR by immunofluorescent staining as described in Figure S5C (H).

Experiments in this figure were performed three times except for those in (A), (B), and (D) twice.

Table S1. List of 114 interacting proteins of ALDOA identified by mass spectrometry. Related to Figure 1

Gene name	Score	Peptide hits
Tubb	1635.86	1303
Tuba	1134.97	811
Myh9	904.16	630
Actb	376.65	339
Fn1	364.07	204
Mybb1a	314.39	186
Acta	312.74	270
Flna	297.67	152
Actc1	280.24	232
Fdft1	238.92	12
Gapdh	237.73	162
Lrpprc	218.41	127
G3bp1	212.72	115
Pkm	212.72	115
Flnb	205.67	107
Hnrnpk	169.5	87
Acadvl	169.28	88
Irgm1	160.29	81
Phgdh	151.49	86
H2afx	141.74	102
Actg2	140.48	127
Dync1h1	128.93	59
Ppib	126.52	78
Idh2	121.63	62
Sec22b	114.29	71
Tbc1d10a	112.25	60
Smc2	105.82	56
Glud1	104.58	57
Hk2	104.29	52
Sec31a	103.96	52
Tln1	98.71	57
Hist1h3b	97.19	74
Cltc	94.79	52
Gnas	91.46	47
Tubg	90.35	50
Myl6b	88.34	61
Tomm70a	80.83	45
Rps16	75.05	40
Mcm5	74.93	39
Rcc1	74.04	48
Rpl13a	69.69	40
Gpd2	68.22	38
Lima1	67.29	33
Acad9	66.41	37
Eno1	65.5	36
Srpr	63.35	34
Hsd17b12	63.19	35
Ap1b1	61.59	33
Nop2	59.73	33
Ddx50	59.43	33
Cul4a	57.07	33
Cpsf2	56.91	33
Arhgef7	52.55	28
Prdx1	50.78	29
Igf2bp1	50.57	27
Stag2	49.69	27
Nsdhl	48.65	25

Utp3	48.11	26
Gaa	47.53	23
Inf2	46.59	24
Rpl27a	46.38	25
Hk1	46.38	25
Pdha1	45.29	23
Atp6v1c1	44.95	24
Alyref	44.81	22
Dbn1	41.55	22
Capn2	41.08	23
Prps1	40.59	21
Capza2	38.44	22
Hyou1	37.53	20
Atp6v1a	37.04	19
Smc1a	36.87	21
Ascc2	35.48	18
Srprb	34.71	20
Tbc1d5	33.71	18
Orc1	30.74	16
Vps41	28.91	16
Mta1	27.04	19
Dcakd	26.96	16
Myl12b	26.44	17
Mdc1	26.39	14
Rps20	25.72	15
Sptan1	24.73	12
Psme4	22.53	12
Ints7	21.97	12
Ebna1bp2	20.42	12
Pc	18.66	12
Rbm14	17.14	10
Nhp2l1	15.92	9
Aldh16a1	14.48	8
Atp6v1h	14.43	7
Morf4l1	13.54	8
Ablim1	13.52	8
Golga2	11.75	7
Dennd2a	11.34	6
Tpi1	10.52	6
Chaf1b	10.25	5
Myl6b	8	5
Rif1	7.99	4
Atp6v1e1	7.78	5
Ugdh	7.69	4
Tbc1d15	7.43	4
Rock2	6.45	4
Foxk1	5.93	3
Osbpl3	5.75	3
Calcoco1	5.67	3
Pfkm	5.64	3
Pfkp	5.49	4
Atp6v0d1	5.33	3
Atp6v1b2	4.74	3
Nlrx1	4.53	3
Trpv4	4.01	2
Tango6	4.01	2
Ifi205a	3.69	3
